# A Comparative Genomic Study in Schizophrenic and in Bipolar Disorder Patients, Based on Microarray Expression Profiling Meta-Analysis

**DOI:** 10.1155/2013/685917

**Published:** 2013-03-10

**Authors:** Marianthi Logotheti, Olga Papadodima, Nikolaos Venizelos, Aristotelis Chatziioannou, Fragiskos Kolisis

**Affiliations:** ^1^Neuropsychiatric Research Laboratory, Department of Clinical Medicine, Örebro University, 701 82 Örebro, Sweden; ^2^Metabolic Engineering and Bioinformatics Program, Institute of Biology, Medicinal Chemistry and Biotechnology, National Hellenic Research Foundation, 48 Vassileos Constantinou Avenue, 11635 Athens, Greece; ^3^Laboratory of Biotechnology, School of Chemical Engineering, National Technical University of Athens, 15780 Athens, Greece

## Abstract

Schizophrenia affecting almost 1% and bipolar disorder affecting almost 3%–5% of the global population constitute two severe mental disorders. The catecholaminergic and the serotonergic pathways have been proved to play an important role in the development of schizophrenia, bipolar disorder, and other related psychiatric disorders. The aim of the study was to perform and interpret the results of a comparative genomic profiling study in schizophrenic patients as well as in healthy controls and in patients with bipolar disorder and try to relate and integrate our results with an aberrant amino acid transport through cell membranes. In particular we have focused on genes and mechanisms involved in amino acid transport through cell membranes from whole genome expression profiling data. We performed bioinformatic analysis on raw data derived from four different published studies. In two studies postmortem samples from prefrontal cortices, derived from patients with bipolar disorder, schizophrenia, and control subjects, have been used. In another study we used samples from postmortem orbitofrontal cortex of bipolar subjects while the final study was performed based on raw data from a gene expression profiling dataset in the postmortem superior temporal cortex of schizophrenics. The data were downloaded from NCBI's GEO datasets.

## 1. Introduction

Schizophrenia (SZ) and bipolar disorder (BD) are approached and studied as diseases with aberrant functions of the neurotransmitter systems, as neurodevelopmental diseases or generally complex diseases caused by multiple genetic and environmental factors. Recently they have started to be studied as systemic diseases; thus a combination of disturbed biological systems and genes of small contribution is believed to cause their expression [1, 2].

Altered membrane composition of the cells, aberrant membrane phospholipid metabolism [3, 4], dysfunctional tyrosine, and other amino acid (AA) transport systems [5–11] evidence the systemic nature of SZ disease. Moreover, failure of niacin skin test implying reduced arachidonic acid (ARA) in cell membranes of schizophrenics [12] and abnormalities in muscle fibers [13] constitute such indications. The same holds for BD, which can also be considered a systemic disease. Aberrant tyrosine, and other AA transport systems, in cells from BD disorder patients [14, 15], aberrant signal transduction [16], and abnormal membrane composition and metabolism support the notion of BD being a systemic disease as well [17, 18].

Studying these disorders through this holistic approach, we presume the membrane phospholipid hypothesis, namely, that aberrant AA transport mechanisms and the disturbed cell membrane composition are highly correlated. AAs are transported though cell membranes with specific transporter/protein transport systems, which perform active transport of AAs from one side of the cell membrane to the other [19]. These AA transporters are embedded in the cell membranes; thus their structure and functionality interact with the membrane composition and functionality, as well as with membrane fluidity and enzymatic activity [9, 20]. Particularly, a membrane defect would impact, for example, the functionality of the tyrosine transporters as well as the permeability of the membranes [2, 5].

**The Membrane Theory**

The membrane theory of mental diseases is related with two primary abnormalities: an increased rate of removal of essential fatty acids (EFA) from the membrane phospholipids, combined with a reduced rate of incorporation of fatty acids (FA) into membrane phospholipids [21]. Some SZ study findings that relate the expression of the disease with the membrane hypothesis are studies based on postmortem and blood samples showing reduction of docosahexaenoic acid (DHA) and ARA in cell membranes independently of the disease state and magnetic resonance spectroscopy (MRS) studies revealing decreased levels of phosphomonoesters (phospholipid membrane synthesis precursors) and higher levels of phosphodiesters (phospholipid metabolism products) in SZ patients compared to control patients [22]. Also, the niacin skin flush test is indicative of a membrane dysfunction resulting in an inflammatory dysfunction [12]. In addition, phospholipase A2 (PLA) calcium (Ca) dependent type has been shown to have an increased activity and PLA Ca independent type a decreased activity. The latter is considered quite important finding, as the A2 enzyme catalyzes the breakdown of FA [23].

Similar findings suggest cell membrane dysfunction in BD. 31P-MRS magnetic resonance spectroscopy (MRS) measures phosphorus metabolites in the organs [24]. Phosphomonoester levels are measured in BD depressed patients with MRS. Phosphomonoesters are measured as being higher in these patients compared to control subjects and lower in asymptomatic patients. Abnormal functionalities in signal transduction pathways are also repeated in several studies including overactivated phosphatidylinositol and G-protein pathways, as well as altered membrane protein kinase C and adenyl cyclase enzyme pathways. PLA enzyme activity and Ca release are involved in the membrane hypothesis of BD [17].

**Amino Acid Transporters**

The transport of AAs into the cell membranes of the blood brain barrier (BBB) is mediated by many transport systems. Three basic active transporters result in the AA flux from and into all types of cells (including brain cells). The primary active transport mechanism is an adenosine triphosphatase (ATPase) that exchanges sodium (Na) and potassium (K) ions, contributing in the maintenance of the ion gradients of the cells, known as sodium-potassium adenosine triphosphatase (Na,K-ATPase). These ion gradients in combination with other ions and gradients are utilized by the secondary active transport mechanisms for the influx of specific AAs into the cells. The secondary active transport through these AA influxes sets also an AA concentration gradient in the cells, which, in combination with Na+ exchange, is further utilized by the tertiary active transport mechanisms for transport of another group of AAs in and out of the cells. AAs may be transported via different AA transport mechanisms. An alteration in any of the active transport mechanisms could result in an aberrant AA transport into the cells [10, 25].

**Aim of the Study**

The aim of our meta-analysis was to interpret the results of comparative genomic profiling studies in schizophrenic patients as compared to healthy controls and in patients with BD and try to relate and integrate our results with an aberrant AA transport through cell membranes.

## 2. Materials and Methods 

### 2.1. Microarray Datasets

Four human datasets were used, by downloading submitted raw data (Cel files) from corresponding studies, available at the Gene Expression Omnibus (GEO) database of National Center for Biotechnology Information (NCBI) [26].

1. The first study has the GEO Accession number GSE12654 and the microarrays preparation followed the guidelines of MIAME in the way it is described in [27]. RNA from postmortem brain tissues (Brodmann's Area 10) of 15 schizophrenic and 15 BD affected patients and 15 control healthy subjects was hybridized on Affymetrix HG-U95 Arrays. After quality control stage in this study, 11 schizophrenic, 11 BD and 15 control subjects were used for further bioinformatic analysis.
2. The second study has the GEO Accession number GSE5389, and the microarrays preparation followed the guidelines of MIAME in the way it is described in [28]. RNA extracted from human postmortem brain tissue (Brodmann's Area 11) from 15 adult subjects with BD and 15 healthy control subjects was hybridized to Affymetrix HG-U133A GeneChip to identify differentially expressed (DE) genes in the disease state. After quality control in this study, 10 BD and 11 control subjects were used for further bioinformatic analysis.
3. The third study has the GEO Accession number GSE21935, and the microarrays preparation followed the guidelines of MIAME in the way it is described in [29]. 60 postmortem RNA samples derived from brain tissue (Brodmann's Area 22) of schizophrenic and control patients were hybridized to the Affymetrix HG-U133 Plus 2.0 Array. After quality control stage samples from 19 control and 23 SZ subjects were subjected to bioinformatic analysis.
4. The fourth study has the GEO Accession number GSE12649, and the microarrays preparation followed the guidelines of MIAME in the way it is described in [30]. RNA samples were extracted from postmortem brain tissue (Brodmann's Area 46) of 35 BD subjects, 35 SZ subjects, and 35 healthy control subjects. The RNA was applied to the Affymetrix HG-U133A GeneChip. After quality control stage in this study, 35 SZ, 33 BD samples, and 34 control samples were finally subjected to bioinformatic analysis.

### 2.2. Analysis of Microarray Data 

The raw signal intensity data of each study were imported into the Gene Automated and Robust MicroArray Data Analysis (Gene ARMADA) software tool [31] for versatile, microarray data analysis. In order to extract the signal intensities from the raw data, specific steps were followed: background correction was performed with the gcRMA method and was followed by Quantile normalization. The negative intensity values were treated with the minimum positive and noise method and then summarization followed with the Median Polish method. The data were transformed in log⁡_2_⁡ values. In each analysis two experimental conditions were always selected: the disease condition and its corresponding control condition. Genes that were characterized as absent in more than 40% of the samples in each experimental condition were excluded from further analysis. The missing values were imputed using the *k*-nearest neighbor (*k*-NN) algorithm. All the steps of the microarray analysis were common for all the extracted datasets.

### 2.3. Statistical Analysis

The probe sets that were differentially expressed in the disease samples compared to the control healthy samples were selected by two-tailed Student's t-test. The lists of the DE probe sets were defined by applying the following criteria in each dataset: (i) 1.3 or greater-fold change (FC) of the mean expression in all studies, except for the fourth study of BD samples compared to controls with FC > 1.2 (small number of DE genes with stricter cutoff) and (ii) *P* value threshold below 0.05. The *P* value distribution for each gene list was used to estimate the False Discovery Rate (FDR) levels. The final gene list corresponds to an FDR < 0.05. The statistical analysis was also performed in the Gene ARMADA software. 

### 2.4. Prioritized Pathway/Functional Analysis of Differentially Expressed Genes

In order to derive better insight into the biological processes related to the DE genes, the lists of significant genes from each microarray analysis were subjected to statistical enrichment analysis using the Statistical Ranking Annotated Genomic Experimental Results (StRAnGER) web application [22]. This bioinformatic tool is using gene ontology term (GOT) annotations and KEGG pathways as well as statistical overrepresentation tests further corrected by resampling methods, aiming to select in a prioritized fashion those GOTs and pathways related to the DE genes, that do not just have a high statistical enrichment score, but also bear a high biological information, in terms of differential expression. Specifically gene ontology (GO) based analysis and KEGG-based analysis result in a list of GO terms and KEGG pathways, respectively, based on hypergeometric tests with values <0.05, which have been reordered according to bootstrapping to correct for statistical distribution-related bias. 

### 2.5. Prioritizations of Putative Disease Genes

In order to prioritize the gene list of interest according to the functional involvement of genes in various cellular processes, thus indicating candidate hubgenes, after inferring the theoretical topology of the GOT-gene interaction network delineated, we used the online tool GOrevenge [32] with the following settings: Aspect: BP (Biological Process), Distance: Resnik, Algorithm: BubbleGene, and Relaxation: 0.15. By adopting these settings we are able to exclude from the interaction network the bias relating to the presence of functionally redundant terms, describing the same cellular phenotypic trait, and thus assessing the centrality, namely, the correlation of the specific genes to certain biological phenotypes in an objective way.

Finally, BioGraph [33] is a data integration and data mining platform for the exploration and discovery of biomedical information. The platform offers prioritizations of putative disease genes, supported by functional hypotheses. BioGraph can retrospectively confirm recently discovered disease genes and identify potential susceptibility genes, without requiring prior domain knowledge, outperforming other text-mining applications in the field of biomedicine.

## 3. Results and Discussion

### 3.1. Differentially Expressed Probesets

**Figure 1:**
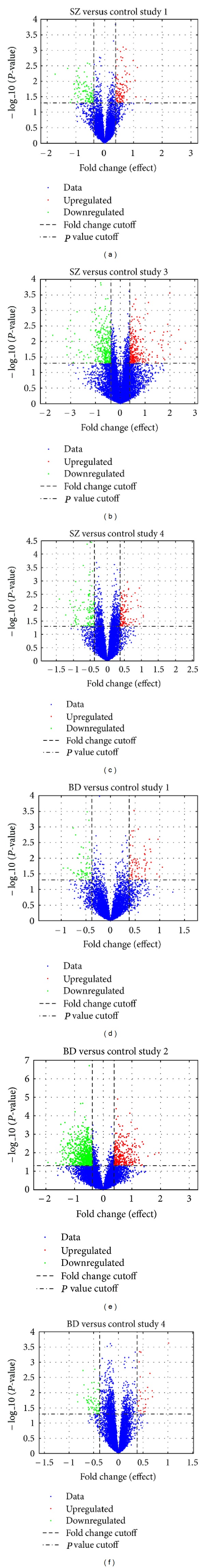
Volcano plots of DE probesets, generated from two-tailed Student's t-test. Upregulated genes in the disease state are depicted with red-colored spots and downregulated genes with green-colored spots. The first three plots (a, b, c) represent DE genes in SZ patients from first, third, and fourth studies, respectively, and the following three plots (d, e, f) represent DE genes in BD patients from first, second, and fourth studies, respectively. FC ratio between gene expression in disease state and healthy state is depicted in the horizontal axes for each dataset in log⁡_2_⁡ scale, and *P* values in −log⁡_10_⁡ scale are depicted in vertical axes. All plots are similar in most studies, except for plot (e), which shows more green and red spots. This fact means that the number of DE genes is similar in most studies but in study 2 there is a greater number of statistically significant genes in comparison to other plots.

After the microarray analysis and the statistical selection, lists of DE probesets for each dataset occurred. From the first and fourth studies' analysis, four lists of significantly differentiated probesets were generated: two after comparison of SZ and control subjects and two after comparison of BD and control subjects. The second study (comparison of BD patients to control subjects) resulted also in a list of DE probesets and the third study in another list of DE probesets (SZ subjects compared to control subjects). The differentiated probesets from each case are depicted in representative volcano plots (Figure 1).

#### 3.1.1. Differentially Expressed Genes in Each Study

In postmortem studies the alterations in the gene expression are usually lower than twofold [29]. For each study, transcripts of interest and of particular expression alterations are described in the following paragraphs. The lists of DE genes for each study are presented in Supplementary Tables 1–6 (available online at doi:10.1155/2013/685917). Information about the protein products arising from the DE genes has been provided mainly from the Reference Sequence (RefSeq) database of NCBI [34].

**First Study**

Statistical analysis of the gene expression profile of SZ and BD patients as compared to controls is summarized in Table 1. The number of DE genes is 196 and 134 respectively.

In SZ patients, transcripts related to the membrane hypothesis show altered expression. Lipases LPL and LIPA, downregulated phosphodiesterases ENPP2 and PDE8A, downregulated phosphoinositide PIK3R4, PNPLA4 phospholipase are related to membrane metabolic processes. ENPP2 and PDE8A dysregulation could also be related to previous MRS studies revealing different levels of phosphodiesters in SZ patients [23]. Some genes encoding proteins of signal transduction pathways, for example, downregulated G protein-coupled receptors GPR37 and GPRC5B, downregulated kinase activity encoding genes PIK3R4 and AATK, or SST somatostatin and CX3CR1 chemokine receptor can also be related to membrane dysfunctions [17]. Genes encoding ion homeostasis seem to be dysregulated as well. NPY, GRIN2A, and CACNA1C all annotated to Ca ion transport (provided by Gene Ontology Annotation UniProt Database) are DE. Also expression of manganese ion binding genes and copper ion binding genes (provided by Gene Ontology Annotation UniProt Database), such as MT1X, is affected. KCNQ2 encoding K voltage-gated channel is overexpressed.

In BD patients of the same study transcript ATP1A3, expressing Na,K-ATPase is downregulated. This ATPase is very important for the normal regulation of the primary active transport mechanism of the cells [29]; thus it affects indirectly the normal function of the AA active transport into the cells. Other dysregulated genes contribute to abnormal K binding and transport (provided by Gene Ontology Annotation UniProt Database): SLC12A5, KCNK3, and KCNK1 are downregulated. POLR2 K encoding phosphodiesterase 6D is upregulated. This fact complies with dysregulated membrane phospholipid metabolism, as phosphodiesters are products of this metabolic pathway [17]. SLC7A8 gene is overexpressed. The importance of this gene relies on the fact that it is encoding transmembrane Na-independent AA transport proteins of the L system. LAT1 protein complex, which is specifically expressed from SL7A8 gene, is a tertiary active transporter and mediates tyrosine, tryptophan, and other neutral AA transport systems through cell membranes [19].

**Second Study**

Statistical analysis of the gene expression profile of BD patients as compared to controls is summarized in Table 2. The number of DE genes is 1035.

Many transcripts regulating ion transport are shown to be downregulated in this study: SCN1A, KCNK1, TRPC1, ATP6V1A, and ATP5G3. Many metallothionein encoding genes (provided by Gene Ontology Annotation UniProt Database) (MT1X, MT2A, MT1E, MT1M, MT1H, MT3, MT1A, and MT1G) are overexpressed. The latter genes combined with downregulated genes COX11, PAM, and RNF7 seem to result in abnormal copper ion binding, because their protein products are involved in this pathway (provided by Gene Ontology Annotation UniProt Database). Genes, encoding ATPases related to Ca++ (ATP2B1, ATP2B2) and H+ (ATP5G3, ATP6AP2, ATP6V1A, ATP6V1D, ATP6V1G2) transporting (provided by Gene Ontology Annotation UniProt Database), are downregulated. The protein encoded by the overexpressed ATP1B1 gene is a member of the family of Na+/K+ and H+/K+ ATPases, as well as a member of the subfamily of responsible proteins for establishing and maintaining the electrochemical gradients of Na and K ions across the plasma membranes [29]. PLA2G5 gene encodes an enzyme that belongs to PLA family. It catalyzes the membrane phospholipid hydrolysis to free FA, and in this study it is overexpressed. Overexpressed PLA2G4A also encodes an enzyme of A2 family. It hydrolyzes phospholipids to ARA (provided by RefSeq). ARA is subsequently metabolized into eicosanoids. Prostaglandins and leukotrienes belong to the eicosanoids, and they are lipid-based cell hormones that regulate inflammation pathways and cellular thermodynamics. The catalyzed hydrolysis also results in lysophospholipids that are further utilized as platelet-activating factors. High Ca++ levels and phosphorylation activate the enzyme (provided by RefSeq). 37 genes encoding proteins involved in magnesium ion binding (provided by Gene Ontology Annotation UniProt Database) show altered expression. Phosphoinositide-3-kinases encoded by downregulated genes PIK3C3, PIK3CB, and PIK3R1 encode phosphoinositide 3-kinases (PI3 K). These kinases are involved in signaling pathways, and their receptors are located on the outer cell membranes [17].

**Third Study**

Statistical analysis of the gene expression profile of SZ patients as compared to controls is summarized in Table 3. The number of DE genes is 122.

The membrane-related protein encoded by the overexpressed ABCA1 gene is a member of ATP-binding cassette (ABC) transporter proteins superfamily. ABC proteins mediate transport of many molecules across extra- and intracellular membranes. ABC1 transporter subfamily's substrate is cholesterol; thus its function is affecting the cellular lipid removal pathway. This gene is related to Tangier's disease and familial high-density lipoprotein deficiency (provided by RefSeq). Apart from ABCA1 gene, also SLC27A3, HSD11B1, CHPT1, and GM2A genes encoding proteins associated with lipid metabolic processes (provided by Gene Ontology Annotation UniProt Database) present a different expression in SZ patients compared to controls. In the DE list CACNB2 is present as an overexpressed gene. This gene encodes a subunit of a voltage-dependent Ca channel protein which is a member of the voltage-gated Ca channel superfamily (provided by RefSeq). CACNA1B, encoding another Ca channel that regulates neuronal release of neurotransmitter, has been proved to be involved in BD and SZ (provided by RefSeq).

**Fourth Study**

Statistical analysis of the gene expression profiles of SZ and BD patients as compared to controls is summarized in Table 4. The number of DE genes is 216 and 205, respectively.

In SZ patients of these study genes ATP2B2 and ATP2B4 are downregulated and upregulated, respectively. These genes encode proteins that belong to the family of P-type ATPases. These enzymes regulate primary ion transport. These two specific ATPases are very important for the homeostasis of Ca in the cell, as they catalyze cellular efflux of bivalent Ca ions from cells against great concentration gradients (provided by RefSeq). Ca ion homeostasis and Ca ion transport (provided by Gene Ontology Annotation UniProt Database) are also dependent on some other genes dysregulated in this study, such as upregulated NPY, RYR3, and ITPR2 and downregulated CXCL12. Two metallothionein encoding genes MT1X and MT1H are overexpressed. After pathway analysis, these genes, in concert with the differentiated expression of several other genes, seem to affect zinc ion binding and copper ion binding (provided by Gene Ontology Annotation UniProt Database).

In BD patients of the fourth study ATP1A2 is overexpressed. The protein expressed by this gene is a member of P-type cation transport ATPases and belongs to the subfamily of Na,K-ATPases. It belongs to integral membrane proteins, responsible for establishing and maintaining the electrochemical gradients of Na and K ions across the plasma membrane. These gradients are very important for osmoregulation, for Na-coupled transport of many organic and inorganic molecules, and for nerve and muscle electrical excitability. The catalytic subunit of Na,K-ATPase is encoded by multiple genes (provided by RefSeq). PLA2G16 is downregulated. The protein encoded by this gene belongs to a superfamily of PLA enzymes. PLA regulates adipocyte lipolysis and release of FA through a G-protein coupled pathway involving prostaglandin and prostaglandin receptors. It belongs to the phospholipase C enzymes that are activated by G-coupled regulatory pathways, such as serotoninergic 5-HT2 pathways (provided by RefSeq). Finally overexpressed metallothioneins MT1X, MT1M, MT1H, and MT1M may result in copper ion binding dysfunctions, as they are involved in this biological function (provided by Gene Ontology Annotation UniProt Database).

#### 3.1.2. Common Differentially Expressed Genes in the Examined Studies

**Figure 2:**
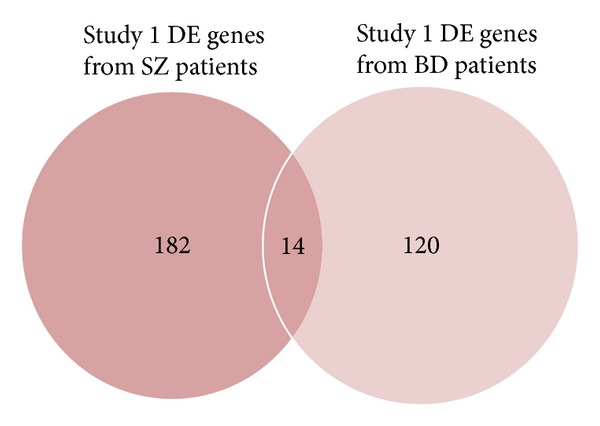
Venn diagram drawn based on DE genes in SZ and BD patients compared with controls of the first study from Brodmann's Area 10 (cognitive functions, goal formation functions). The common DE genes are represented by the intersection of the two circles.

**Figure 3:**
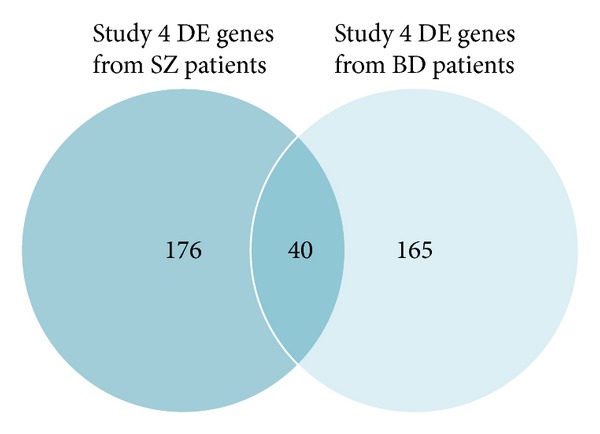
Venn diagram drawn based on DE genes in SZ and BD patients compared with controls of the fourth study from Brodmann's Area 46 (attention and working memory functions). The common DE genes are represented by the intersection of the two circles.

**Figure 4:**
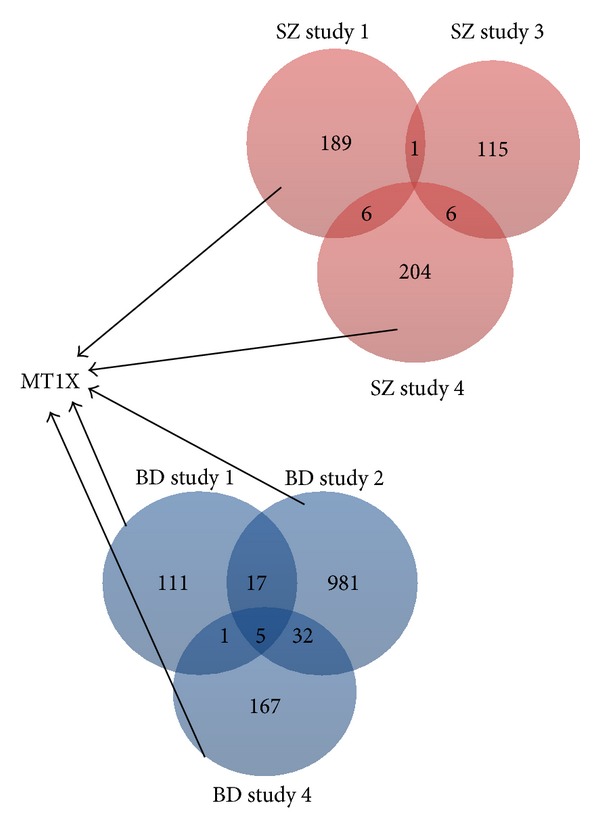
Venn diagram drawn based on DE genes in SZ and BD patients compared with controls. Red circles represent number of DE genes of SZ samples and blue circles represent number of DE genes of BD samples. MT1X is DE in all studies apart from study 3. All studies include samples from frontal cortices, apart from study 3.

In the first and fourth study SZ gene expressions and BD gene expressions are compared to the same control gene expressions. Common DE genes in SZ and BD patients compared to the same control subjects, for example, in the first (Figure 2) and fourth (Figure 3) examined studies are depicted in Tables 5 and 6, respectively. The genes present in lists of statistical significant genes derived from SZ patients' expression profiles are given in Table 8. The common genes in all DE genes of BD patients compared to control groups from all related studies are presented in Table 7. MT1X gene is overexpressed in all studies, in all gene expression comparisons, except for the second study, where it is not among the statistical significant genes as shown in Figure 4.

Among the common DE genes in BD and SZ patients of the first study HTR2C is an interesting gene. Serotonergic pathway is highly related to psychiatric disease expressions. The neurotransmitter serotonin (5-hydroxytryptamine, 5-HT) causes many physiological functions after binding to receptor subtypes, such as 5-HT2 family of seven-transmembrane-spanning, G-protein-coupled receptors. These receptors activate phospholipase C and D signaling pathways. This gene encodes the 2C subtype of serotonin receptor, and its RNA editing is predicted to alter AAs within the second intracellular loop of the 5-HT2C receptor and generate receptor isoforms that differ in their ability to interact with G proteins and the activation of phospholipase C and D signaling cascades, thus modulating serotonergic neurotransmission in the central nervous system. Studies in humans have reported abnormalities in patterns of 5-HT2C editing in depressed suicide victims. Three transcript variants encoding two different isoforms have been found for this gene. This gene is downregulated in both diseases [17]. Serotonin neurotransmitter has been proved to play an important role in emotional, sexual, and eating behavior and in other symptoms of mental diseases, such as hallucinations. Many drugs used for the treatment of these diseases are serotonin agonists. Upregulated ADD2, GGRRF1, and MT1X encode proteins related to metal ion binding. HTR2C, DARC, and GRK5 products participate in signal transduction pathway. 

The protein encoded by SDC4 gene is a transmembrane heparan sulfate proteoglycan that functions as a receptor in intracellular signaling. Downregulated KCNK1 gene encodes one of the members of the superfamily of K channel proteins, and it has been previously reported as dysregulated in BD patients [35]. The downregulation of this gene may affect the passive transport of K into the cells.

NPY (neuropeptide) and GABA-system-related SST (somatostatin) are downregulated in two of our SZ studies. These genes have been reported in many studies as candidate psychosis genes [36]. They have also been related to SZ. Earlier studies reveal also downregulation of these specific genes. Neuropeptide genes are involved in working memory functions [37]. In psychiatric diseases working memory and neurodegeneration have been suggested as possible abnormal functions of the prefrontal cortex. These genes seem to be implicated in these functions [36]. PALLD gene, myocardial infarction-related gene, has also been reported as dysregulated in SZ [38]. The protein encoded by AQP4 gene is involved in the regulation of the water homeostasis. Upregulation of this gene has been already reported and has been related to white matter hyperintensity, observed in MRS studies of BD patients [27]. Generally there are no common genes in all three SZ datasets. This could be explained by the fact that there are region-specific alterations in SZ, and our SZ raw data were extracted from different brain regions.

### 3.2. Pathway Analysis

The lists of statistical significant genes of each study were submitted to StrAnGER web application elucidating overrepresented GO terms. The results of GO-analysis for each dataset are presented in Supplementary Tables 7–12.

In the first study, StRAnGER analysis in the SZ-related DE gene list indicated that K ion binding and transport are two of the statistical significant altered GO terms. These processes are very important for the maintenance of K ion gradients in the cells. K ion transport regulates the fluxes of K ions from and into the cells via some transport proteins or pores [19, 25].

StRAnGER analysis in the BD-related DE gene list indicated altered synaptic pathways. Synaptic pathways and genes have been reported earlier as possible dysfunction factors in BD [39]. G-protein pathways are also related to neurotransmitter receptors and particularly to serotonergic receptors, most studied in BD as part of serotonergic pathway [17]. Ca transport, protein tyrosine kinase, and phosphoinositide binding are involved in signal transduction pathways. Several studies of BD patients have shown abnormalities in the phosphoinositol/protein kinase C (PKC) signaling system. One such study has demonstrated significantly higher concentrations of 4,5-bisphosphate (PIP2) in the platelet membranes of patients in the manic phase of BD; they also found that the levels of PIP2 increased when cycling from the euthymic state into the manic state. Additionally, the activity of platelet PKC was found elevated in patients, during a manic episode of BD. Additionally several independent studies have shown increased concentrations of the stimulatory alpha subunit (G_as_) of G-protein in the brains of BD patients, specifically in the frontal, temporal, and occipital cortices. Other studies have suggested there is also increased presence/activity of G-proteins in the leukocytes of untreated manic patients and the mononuclear leukocytes of bipolar, but not unipolar, patients. Currently, there is no evidence to indicate that the increased concentration of G_as_ is caused by gene mutations; it has been suggested that they could be caused by a change in any of the biochemical pathways leading to the transcription and translation of the G_as_ gene [40]. Copper ion binding belongs to the significant GOTs as well.

In the second study copper ion binding, magnesium ion binding, chloride channel activity, chloride transport, postsynaptic membrane, and inositol or phosphatidylinositol phosphatase activity represent significantly differentiated GOTs.

In study 3 and 4 defense response, immune response, and inflammatory response GOTs are present in the overrepresented GOTs. The inflammatory system is strongly related to these mental disorders, and the immune underlying mechanisms remain mainly obscure [41]. Lipid metabolic process is also a statistically significant GOT altered in study 3.

Dysregulated neurotransmitter systems in the central nervous system of BD and SZ patients have been systematically reported [2, 4]; thus central nervous system development is among the GO terms resulting from pathway analysis of study 3 BD DE list. Copper ion binding, chloride ion binding, and signal transduction pathways seem to be affected.

Copper ion binding is present in almost all lists of significantly altered GO terms. Signaling pathways are among the KEGG pathways that appear more often as overrepresentative pathways (Supplementary Table 13).

We also performed GO analysis in the 68 genes, shown schematically in Figure 4, that were present in at least two of the BD or SZ DE lists.  Table 9 summarizes the GO terms of this pathway analysis. ATP binding is essential for the maintenance of the ion gradients in the cell. ATP is universally an important coenzyme and enzyme regulator [19].

### 3.3. Identification of Candidate Hub Genes

In order to expand our knowledge regarding which genes have critical role among the common DE genes in BD datasets, we used the online tool GOrevenge [32], which performs prioritization of the gene list taking into consideration the centrality of each gene, as described in the GO tree. The 68 genes found differentiated in at least two BD- or SZ-related studies were submitted to GOrevenge, and the analysis was performed based on GO annotations for Homo sapiens as described in materials and methods section. A prioritized list of genes, containing candidate linker genes, that is, genes participating in many different cellular processes, was derived (Table 10). Among them, three genes, namely, APOE, RELA, and NPY, have also been found as statistically significantly differentiated in at least two of either SZ or BD DE gene lists.

### 3.4. Prioritizations of Putative Disease Genes

By setting SZ and BD as concept, the relation of each gene with the BD and SZ was assessed, and the 68 genes found differentiated in at least two BD- or SZ-related studies were prioritized by BioGraph algorithm as shown in Tables 11 and 12, respectively. The genes are prioritized according to their score which is a statistical enrichment measure of the relevance of each gene with the inquired context (here specified as either BD or SZ) to the total relations (references) of the gene in the universe of terms. In this way, the user can derive which of its genes are already associated and in what extent with a given disease or generally biological term and which of them represent novel findings with respect to the investigated pathological phenotype. APOE, RELA, and NPY have also high scores and are among the ten top genes related either to the BD or SZ after the prioritization of genes in BioGraph. These three genes have been shown to play a major role in the examined studies, after different bioinformatic analyses. NPY has been reported as a candidate psychosis gene, as aforementioned.

APOE regulates cholesterol of the central nervous system; thus any alteration in APOE levels may result in abnormal brain function. APOE has been mostly related to Alzheimer's disease [42].

Genotyping studies and Western plot analysis have shown differences of APOE in SZ patients. Abnormal cholesterol metabolism has been associated with SZ as well. High levels of three different apolipoproteins in brains of patients with psychiatric disorders may indicate aberrant central nervous system lipid metabolism. Additionally, APOE has been implicated in inflammation pathways, after studies on mice revealing possible action of APOE as inflammatory response inhibitor. Inflammation pathways are considered candidate mechanisms responsible for the pathogenesis of several mental disorders and mainly of SZ [42].

RELA, v-rel reticuloendotheliosis viral oncogene homolog A (avian), is also involved in immune and inflammatory responses, as it encodes the main component of the NF-*κ*B complex. NF-*κ*B has been related indirectly to SZ, as it is highly correlated to SZ involved cytokines: interleukin-1*β* (IL-1*β*), IL-1 receptor antagonist (IL-1RA), IL-6, and tumor necrosis factor-*α* (TNF-*α*). NF-*κ*B is a regulator of cytokines' expression, and proinflammatory cytokines activate NF-*κ*B. NF-*κ*B is present in synaptic terminals and participates in regulation of neuronal plasticity. NF-*κ*B regulates genes that encode subunits of N-methyl-D-aspartate receptors, voltage-dependent Ca channels and the Ca-binding protein calbindin, cell survival factors, including Bcl-2, Mn-SOD, and inhibitor of apoptosis proteins (IAPs) and cell death factors, including Bcl-x(S) and Bax. All these genes are related to neurotransmission, and altered expression of several of them has been reported in previous SZ postmortem brain studies [43].

## 4. Conclusions

The aim of the study was to interpret the results of comparative genomic profiling studies in schizophrenic patients as compared to healthy controls and in patients with BD and try to relate and integrate our results with an aberrant AA transport through cell membranes. Starting from genomewide expression data, the analysis focused on genes and mechanisms involved in AA transport through cell membranes. We performed transcriptomic computational analysis on raw data derived from four different studies. Moreover, a multistage, translational bioinformatic computational framework is employed, previously utilized for the molecular analysis of transcriptomic data of atherosclerotic mice models [44], exploiting different methods in order to identify critical altered molecular mechanisms and important central players. In this way, the results derived here do not rely solely on a single stage of significance. They are complying to a systematic screening of the results, exploiting various statistical measures, in a unified analysis pipeline. These measures either exploit the stringent FDR estimations at the single gene level, further filtered to keep those common in between diseases or studies comparisons. Moreover, the consensus gene lists thus derived are corrected through a rigorous, bootstrapping framework, applied in the statistical enrichment analysis of the significant biological processes. Moreover, critical regulatory genes, prioritized by their total number of GO annotations, to the resulting significant GOTs list, are highlighted. It is also examined, whether these genes have been associated with the disease phenotypes of SZ or BD in the broader biomedical literature. The results were eventually analyzed, complying with a meta-analysis context, giving emphasis on common functional patterns mined amid the various studies.

Our bioinformatic analyses of the downloaded datasets demonstrate genes and GOTs associated with ion transport dysregulation (K, Na, Ca, and other ion transports and bindings) resulting in a disturbed primary active transport, suggesting a deficit in transmembrane Na+ and K+ gradients maintenance. Characteristic downregulation of Na+ and K+ transporting ATPases, enzymes responsible for establishing and maintaining the electrochemical gradients of Na and K ions across the plasma membrane, is indicated in the DE gene lists of two of our datasets. They are also upregulated in one dataset (BD patients' expression profiles). Also downregulation of P-type ATPases is reported in the datasets. Altered distribution of specific ions in the cells may affect distributions of other ion groups. A statistical integration of many studies has previously related published data of Na,K-ATPase activity in erythrocytes of BD patients with the expression of the disease [45]. Decreased activity of Na,K-ATPase has been also related to SZ in previous studies [38]. The disturbed primary active transport observed in our study indicates difficulty in maintaining transmembrane ion gradients. This fact should result in disrupted, secondary, active AA transporter Systems A, X-AG, N, and y+, as they couple AA transport to the electrical and chemical gradients initiated by primary active transport. AA exchangers, systems ASC, y+L and L, that transport AAs by antiport mechanisms, may suffer from a deficit of secondary, actively transported AAs they need for the exchange, resulting in a disrupted transport of AAs mainly transported through this third mechanism.

Genes and pathways related to Ca transport agree with abnormalities in Ca signaling, that have been implicated in BD; findings show elevated intracellular Ca concentrations in the platelets, lymphocytes, and neutrophils of BD patients. Ca is very important in most intracellular signaling pathways and in the regulation of neurotransmitter synthesis and release [40].

Phospholipase activity may be dysregulated in BD and SZ diseases, as indicated by altered expression of the genes encoding this enzyme in this study. This alteration has obvious impacts on the phospholipid metabolism of the membrane, as it is a crucial enzyme in this metabolic pathway [23].

A consistent upregulation of MT1X and generally of metallothionein genes is consistent in different datasets. The functional role of metallothioneins in the brain has not been very well characterized [36]. The main function of metallothioneins is to protect neurons from pathological stressing factors. Abnormal expression of genes encoding these proteins may indicate an endogenous reaction to constant oxidative stress [46]. Several studies have suggested involvement of metallothioneins in functions of the central nervous system, such as neuroprotection, regeneration, and cognitive function. Other studies reported that metallothioneins are involved in cellular response, immunoregulation, cell survival, and brain functional restoration. Metallothioneins are mainly produced in astrocytes. Metallothionein overexpression has been also reported as a contributing factor in brain pathologies, such as excitotoxic injury, amyotrophic lateral sclerosis, Alzheimer's disease, and Parkinson's disease. Animal studies have associated substance dependences and learning procedures with metallothioneins. Other prefrontal cortex (PFC) studies have revealed overexpression of metallothioneins in SZ patients. All these studies indicate involvement of metallothioneins in neuroprotection and cognitive functions. A possible neurodegenerative function in the PFC may affect cognitive function in BD and SZ patients. Overexpression of these genes could then be a defense mechanism against these adverse processes. Metallothioneins have also been proposed as possible medical treatment as they have been tested in animal models and have been proved nontoxic [36].

The observed small number of common DE genes among the different studies reflects heterogeneity among the datasets analyzed, which could be explained by both biological and technical reasons. The brain area under study, the microarray platform used, and the selection of patients and controls could contribute to the heterogeneity and should be taken into consideration and duly addressed, ideally at the stage of the experimental design, whenever analogous meta-analysis tasks are envisioned. Highlighting genes that present different expression in different cases, but in the context of a multitiered systematic framework, like the one presented here, could result in molecular interactions, linked with causative, universal, and molecular pathways in mental disorders.

## Supplementary Material

The lists of differentiated transcripts after comparing the gene expression of control and either of SZ or BD patients from each dataset are presented in supplementary tables 1-6. The lists include the Gene ID, the Gene Symbol, the Gene Title, the p-value and the fold change in natural and in log2 scale for each differentially expressed transcript.
The results of GO-analysis for each dataset are presented in supplementary tables 7-12. GOT p-value represents the hypergeometric test p-value score for each GO term. Enrichment represents the ratio of the number of times a GO term occurs in the examined DE gene list to the number of times this GO term exists in the list of the entire microarray (for each study, its respective Affymetrix platform). GO terms presented in italics, are the ones considered most interesting in our study.
Supplementary Table 13: Kegg pathways based analysis. The lists of significantly altered genes from each study were submitted to StRAnGER analysis, elucidating over-represented Kegg terms.

## Figures and Tables

**Table 1:** Number of DE genes and probesets, in SZ and BD patients as compared to healthy controls. Genes are characterized as overexpressed when they present positive FC > |0.37| in log_2_ scale and as downregulated when they present negative FC respectively. Out of 63000 probesets and 10000 genes of the Affymetrix HG-U95 platform, we derived a much smaller number of probesets and genes.

Disease versus control	Overexpressed genes	Downregulated genes	Total DE genes	Total probesets
SZ versus control	103	93	196	203
BD versus control	74	60	134	134

**Table 2:** Number of DE genes and probesets, occurring from comparison of BD gene expression profile and control expression profile. Genes are characterized as overexpressed when they present positive FC > |0.37| in log_2_ scale and as downregulated when they present negative FC, respectively. Out of 45000 probesets and 33000 genes of the Affymetrix HG-U133A GeneChip, we derived a much smaller number of probesets and genes.

Disease versus control	Overexpressed genes	Downregulated genes	Total DE genes	Total probesets
BD versus control	303	732	1035	1162

**Table 3:** Number of DE genes and probesets, occurring from comparison of SZ gene expression profile and control expression profile. Genes are characterized as overexpressed when they present positive FC > |0.37| in log_2_ scale and as downregulated when they present negative FC, respectively. Out of 54921 probesets and 38500 genes of Affymetrix HG-U133 Plus 2.0 Array, we derived a much smaller number of probesets and genes.

Disease versus control	Overexpressed genes	Downregulated genes	Total DE genes	Total probesets
SZ versus control	88	34	122	128

**Table 4:** Number of DE genes and probesets, occurring from comparison of SZ or BD gene expression profile and control expression profile. In case of SZ vs control samples genes are characterized as overexpressed when they present positive FC >|0.37| in log_2_ scale and in case of BD vs control when they present FC >|0.26| in log_2_ scale. Genes are characterized as downregulated when they present the negative FCs respectively. Out of 45000 probesets and 33000 genes of the Affymetrix HG-U133A GeneChips, we derived a much smaller number of probesets and genes.

Disease versus control	Overexpressed genes	Downregulated genes	Total DE genes	Total probesets
SZ versus control	113	103	216	227
BD versus control	69	136	205	210

**Table 5:** The fourteen common DE genes in schizophrenic and BD samples compared to control samples derived from the first study.

Gene symbol	FC (log_2_)	FC (log_2_)	Gene title
	SZ versus control	BP versus control	
SLC25A1	−*0.624219 *	−*0.627028 *	“Solute carrier family 25 (mitochondrial carrier; citrate transporter), member 1"
HTR2C	−*0.511652 *	−0.515884	5-hydroxytryptamine (serotonin) receptor 2C
SYP	−*0.506666 *	−*0.644315 *	Synaptophysin
SERINC5	−*0.476598 *	−*0.564567 *	Serine incorporator 5
CGRRF1	**0.388505**	**0.443519**	Cell growth regulator with ring finger domain 1
SF3B1	**0.434178**	**0.435295**	Splicing factor 3b, subunit 1, 155 kDa
ADD2	**0.476098**	**0.529755**	Adducin 2 (beta)
GRK5	**0.554659**	−*0.593328 *	G protein-coupled receptor kinase 5
UCHL3	**0.587522**	**0.701958**	ubiquitin carboxyl-terminal esterase L3 (ubiquitin thiolesterase)
DARC	**0.642385**	**0.498777**	Duffy blood group, chemokine receptor
SEPT11	**0.651131**	−*0.551204 *	septin 11
MT1X	**0.754667**	**0.966154**	Metallothionein 1X
CEBPD	**0.774212**	**0.726239**	CCAAT/enhancer binding protein (C/EBP), delta
LGALS3	**0.892986**	**0.636527**	Lectin, galactoside-binding, soluble, 3

Downregulation of genes in each disease state compared with controls is represented with negative FC values (fold decrease) and upregulation with positive FC values. Most statistically significant genes, common in SZ and BD, are differentiated in similar way.

**Table 6:** Common DE genes in SZ and BD patients as compared to control samples derived from the fourth study. Top twenty genes (BD) are shown.

Gene symbol	FC (log_2_) SZ versus control	FC (log_2_) BD versus control	Gene title
DERL1	−*0.9218 *	−*0.59278 *	Der1-like domain family, member 1
DDX27	−*0.58735 *	−*0.55081 *	DEAD (Asp-Glu-Ala-Asp) box polypeptide 27
NELL1	−*0.48395 *	−*0.49181 *	NEL-like 1 (chicken)
WDR41	−*0.561422 *	−*0.47103 *	WD repeat domain 41
SST	−*0.56168 *	−*0.47692 *	Somatostatin
ZYX	−*0.55832 *	−*0.4319 *	Zyxin
SSR1	−*0.79829 *	−*0.41544 *	Signal sequence receptor, alpha fibronectin
FSD1	−*0.4133 *	−*0.39578 *	Type III and SPRY domain containing 1
TRIM27	−*0.51857 *	−*0.39195 *	Tripartite motif-containing
TESC	−*0.546183 *	−*0.364501 *	27 Tescalcin
HES1	**0.383441**	**0.32929**	Hairy and enhancer of split 1
MT1H	**0.477326**	**0.329479**	(Drosophila) metallothionein 1H
GJA1	**0.694821**	**0.332313**	Gap junction protein, alpha 1, 43 kDa
TRIL	**0.405464**	**0.343382**	TLR4 interactor with leucine-rich repeats
MT1X	**0.60052**	**0.35402**	Metallothionein 1X
AGXT2L1	**0.816962**	**0.375859**	Alanine-glyoxylate aminotransferase 2-like 1
GREB1	**0.623598**	**0.418634**	Growth regulation by estrogen in breast cancer 1
EMX2	**0.975302**	**0.545582**	Empty spiracles homeobox
GPC5	**0.772653**	**0.591493**	2 glypican 5
ALDH1L1	**1.0583**	**0.599394**	Aldehyde dehydrogenase 1 family, member L1

Downregulation of genes in each disease state is represented with negative FC values (fold decrease) and upregulation with positive FC values. Most statistically significant genes, common in SZ and BD, are differentiated in similar way.

**Table 7:** Genes present in all gene lists from all studies including comparison of gene expression between BD samples and control samples.

Gene symbol	FC BD versus control	FC BD versus control	FC BD versus control	Gene title
	(Study 1)	(Study 2)	( Study 4)	
SDC4	**0.403522**	**0.79702**	**0.323976**	Syndecan 4
MT1X	**0.440635**	**1.1129**	**0.35402**	Metallothionein 1X channel
KCNK1	−*0.416116 *	−*0.5935 *	−*0.280259 *	Potassium,
				SubfamilyK,
				Member 1
MT1H	0.684202	1.09618	0.329479	Metallothionein 1H
POLR3C	**0.563585**	**1.28172**	−*0.335122 *	Polymerase (RNA)
				III (DNA directed)
				PolypeptideC
				(62 kDa)

Downregulation of genes in each disease state is represented with negative FC values (fold decrease) and upregulation with positive FC values. Most statistical significant genes, common in all BD studies are differentiated in similar way.

**Table 8:** Genes present in DE gene lists from all studies including comparison of gene expression between SZ samples with control samples.

Gene symbol	FC SZ versus control	FC SZ versus control	FC SZ versus control	Gene title
	(Study 1)	(Study 3)	(Study 4)	
SRGN	**0.777085**	**0.42152**	—	Serglycin
PRPF4B	**0.563723**	—	0.415853	PRP4 pre-mRNA processing factor 4 homolog B (yeast)
MT1X	**0.754667**	—	0.60052	Metallothionein 1X
GYG2	**0.754525**	—	*0.686934 *	Glycogenin 2
NR4A2	−*0.90769 *	—	−*0.550066 *	Nuclear receptor subfamily 4, group A, member 2
NPY	−*0.568144 *		−*0.406243 *	Neuropeptide Y
SST	−*0.83089 *	—	−*0.561683 *	Somatostatin
PALLD	—	**0.509794**	**0.401231**	Paladin, cytoskeletal
				Associated protein
AQP4	—	**0.449303**	**0.714565**	Aquaporin 4
ARPC1B	—	**0.392173**	−*0.597327 *	Actin-related protein 2/3 complex, subunit 1B, 41 kDa
PVALB	—	−*0.403296 *	−*0.432033 *	Parvalbumin
HSD11B1	—	−*0.413042 *	−*0.538573 *	Hydroxysteroid(11-beta)dehydrogenase1
PHLDA2	—	−*0.452704 *	−*0.455578 *	Pleckstrin homology-like domain, family A

Downregulation of genes in each disease state is represented with negative FC values (fold decrease) and upregulation with positive FC values. Most statistical significant genes, common in SZ studies are differentiated in similar way.

**Table 9:** Overrepresented GO terms extracted from the union of 68 common genes either of BD patients or of SZ patients.

GO annotation	GOT *P*-value	Enrichment
Protein amino acid phosphorylation	0.000254537	6/424
ATP binding	0.000266417	10/1063
Protein binding	0.000833654	19/3248
Transferase activity	0.001557772	8/925
Nucleotide binding	0.001893973	10/1348
Cytoplasm	0.002264782	10/1379
Extracellular region	0.005570074	5/547
Metabolic process	0.0076371	4/414
Multicellular organismal development	0.011932632	5/644
Endoplasmic reticulum	0.018083004	4/514
Zinc ion binding	0.024540778	8/1430
Plasma membrane	0.034457754	4/610

**Table 10:** GOrevenge prioritization. The second column refers to the number of GO terms remaining after GOrevenge pruning, reflecting the centrality of each gene, while the third column refers to the original number of biological process category GO terms of each gene. Top 20 genes are shown. Genes presented in italics are among the statistically significant differentiated genes in at least two of either SZ or BD DE gene lists.

Gene symbol	Remaining GO terms	Original GO terms
TGFB1	56	126
CTNNB1	53	117
BCL2	50	121
SHH	45	142
AKT1	44	73
PSEN1	39	70
WNT5A	38	98
*APOE *	38	54
BMP4	37	128
TNF	37	88
FGF10	36	102
IL1B	35	75
AGT	34	63
P2RX7	33	68
SFRP1	32	81
*RELA *	32	50
TGFB2	32	66
BMP2	32	59
PPARG	31	51
EP300	31	46

**Table 11:** Prioritization of the genes presented in table 11, by BioGraph exploiting unsupervised methodologies for the identification of causative SZ-associated genes. Genes with the higher nineteen scores are shown.

Gene symbol	Score
PVALB	0.172895
SYN2	0.084975
APOE	0.013519
RELA	0.00034
CRK	0.000246
NTRK2	0.000219
MAPT	0.000136
TRIP13	0.000127
NPY	7.39*E* − 05
MT1X	6.19*E* − 05
NR4A2	4.25*E* − 05
SDC4	3.57*E* − 05
PGK1	3.29*E* − 05
PRPF4B	3.21*E* − 05
SST	2.35*E* − 05
TRPC1	2.28*E* − 05
LGALS3	2.19*E* − 05
DUSP6	1.96*E* − 05
BGN	1.66*E* − 05

**Table 12:** Prioritization of the genes presented in table 12, by BioGraph exploiting unsupervised methodologies for the identification of causative BD-associated genes. Genes with the higher nineteen scores are shown.

Gene symbol	Score
PVALB	1.930909595
NTRK2	0.520432786
MAPT	0.000852042
RELA	0.000381239
CRK	0.0002833
NPY	0.000109408
APOE	8.79036*E* − 05
SYN2	6.07336*E* − 05
NR4A2	5.57465*E* − 05
TRPC1	4.28846*E* − 05
SDC4	3.78467*E* − 05
HSD11B1	3.34794*E* − 05
TRIP13	2.26339*E* − 05
SLC12A5	0.000021501
LGALS3	0.000020488
MT1X	1.88525*E* − 05
SST	1.75935*E* − 05
DUSP6	0.000015482
AQP4	1.50416*E* − 05

## Abbreviations

ATPase: Adenosine triphosphatase
AA: Amino acid
ARA: Arachidonic acid
ARMADA: Automate Robust Microarray Data Analysis
BD: Bipolar disorder
DHA: Docosahexaenoic acid
EFA: Essential fatty acids
FA: Fatty acids
FC: Fold change
FDR: False discovery rate
GEO: Gene expression omnibus
GO: Gene ontology
GOT: Gene ontology term
*k*-NN: *k*-nearest neighbor
MRS: Magnetic resonance spectroscopy
NCBI: National Center for Biotechnology Information
PLA: Phospholipase A2
Na,K-ATPase: Sodium-potassium adenosine triphosphatase
StRAnGER: Statistical Ranking Annotated Genomic Experimental Results
SZ: Schizophrenia
Ca: Calcium
Na: Sodium
K: Potassium
G_as_: alpha subunit of G protein
DE: Differentially expressed
PFC: Prefrontal cortex.
