# Supplementary material for: A Comparative Genomic Study in Schizophrenic and in Bipolar Disorder Patients, Based on Microarray Expression Profiling Meta-Analysis
Source: ScientificWorldJournal. 2013 Mar 10;2013:685917. doi: 10.1155/2013/685917 (PMC3608181; doi:10.1155/2013/685917)
Supplement: Supplementary file 1 — The lists of differentiated transcripts after comparing the gene expression of control and either of SZ or BD patients from each dataset are presented in supplementary tables 1-6. The lists include the Gene ID, the Gene Symbol, the Gene Title, the p-value and the fold change in natural and in log2 scale for each differentially expressed transcript. The results of GO-analysis for each dataset are presented in supplementary tables 7-12. GOT p-value represents the hypergeometric test p-value score for each GO term. Enrichment represents the ratio of the number of times a GO term occurs in the examined DE gene list to the number of times this GO term exists in the list of the entire microarray (for each study, its respective Affymetrix platform). GO terms presented in italics, are the ones considered most interesting in our study. Supplementary Table 13: Kegg pathways based analysis. The lists of significantly altered genes from each study were submitted to StRAnGER analysis, elucidating over-represented Kegg terms. [file 685917.f1.pdf]

Supplementary tables

Supplementary tables 1-6: The lists of differentiated transcripts after comparing the gene expression of control and either of SZ or BD patients from each dataset are presented in supplementary tables 1-6. The lists include the Gene ID, the Gene Symbol, the Gene Title, the p-value and the fold change in natural and in log_2 scale for each differentially expressed transcript.

STUDY 1

Table 1: DE genes and probesets, occurring from comparison of SZ and control gene expression profiles. In the case that different probesets correspond to the same gene symbol, the one depicting the higher fold change is shown.

GeneID	Gene Symbol	Gene Title	p-value	Fold Change (natural) SZ vs CONTROL	FoldChange (log2) SZ vs CONTROL
34907_at	AATK	apoptosis-associated tyrosine kinase	0.045733	0.703207	-0.50798
39355_at	ABCE1	ATP-binding cassette, sub-family E (OABP), member 1	0.043055	1.36804	0.452113
1635_at	ABL1	c-abl oncogene 1, non-receptor tyrosine kinase	0.030494	0.733627	-0.44688
34790_at	ACAT2	acetyl-CoA acetyltransferase 2	0.020403	0.604437	-0.72634
32405_at	ACOT11	acyl-CoA thioesterase 11	0.011675	1.45194	0.537979
AFFX-HSAC07/X00351_5_at	ACTB	actin, beta	0.029276	0.720008	-0.47392
36051_s_at	ADD2	adducin 2 (beta)	0.048982	1.39098	0.476098
37708_r_at	ADH5	alcohol dehydrogenase 5 (class III), chi polypeptide	0.029861	1.44127	0.527342
38808_at	ADRM1	adhesion regulating molecule 1	0.015928	0.725849	-0.46226
36148_at	APLP1	amyloid beta (A4) precursor-like protein 1	0.019052	0.762504	-0.39118
41455_at	ARHGAP32	Rho GTPase activating protein 32	0.029405	1.32027	0.400837
40164_at	ARHGDIA	Rho GDP dissociation inhibitor (GDI) alpha	0.012231	0.727453	-0.45907
1784_s_at	ARID4A	AT rich interactive domain 4A (RBP1-like)	0.041792	0.631476	-0.6632
34691_f_at	ARPC4	actin related protein 2/3 complex, subunit 4, 20kDa	0.020407	0.741836	-0.43083
39410_at	ASAP2	ArfGAP with SH3 domain, ankyrin repeat and PH domain 2	0.047849	0.743312	-0.42796
35804_at	ASH2L	ash2 (absent, small, or homeotic)-like (Drosophila)	0.022448	1.38682	0.47178
32720_at	ATG12	ATG12 autophagy related 12 homolog (S. cerevisiae)	0.023338	1.48925	0.574588
35179_at	B3GAT3	beta-1,3-glucuronyltransferase 3 (glucuronosyltransferase I)	0.002742	0.695092	-0.52472
41356_at	BCL11A	B-cell CLL/lymphoma 11A (zinc finger protein)	0.037754	0.735402	-0.4434
40091_at	BCL6	B-cell CLL/lymphoma 6	0.013012	1.52906	0.612642
39114_at	C10orf10	chromosome 10 open reading frame 10	0.005981	1.8188	0.862991
36745_at	C1orf95	chromosome 1 open reading frame 95	0.010807	0.609611	-0.71404
41207_at	C9orf3	chromosome 9 open reading frame 3	0.02402	0.497186	-1.00814
32684_at	C9orf91	chromosome 9 open reading frame 91	0.013568	1.63219	0.706805
40095_at	CA2	carbonic anhydrase II	0.010308	0.633532	-0.65851
38001_at	CACNA1C		0.016793	1.37063	0.45484
36570_at	CALB1	calbindin 1, 28kDa	0.005473	1.71395	0.777323
32543_at	CALR	calreticulin	0.026521	0.664935	-0.58871
31787_at	CAMLG	calcium modulating ligand	0.021448	1.4259	0.511871
33385_g_at	CAST	calpastatin	0.025373	1.46764	0.553502
1942_s_at	CDK4	cyclin-dependent kinase 4	0.007134	0.652069	-0.6169
1052_s_at	CEBPD	CCAAT/enhancer binding protein (C/EBP), delta	0.010592	1.71026	0.774212
450_g_at	CGRRF1	cell growth regulator with ring finger	0.028327	1.30904	0.388505

		domain 1			
32081_at	CIT	citron (rho-interacting, serine/threonine kinase 21)	0.025225	1.34542	0.428053
292_s_at	CLK1 /// PPIL3	CDC-like kinase 1 /// peptidylprolyl isomerase (cyclophilin)-like 3	0.023157	0.698292	-0.5181
497_at	CLN3	ceroid-lipofuscinosis, neuronal 3	0.023927	0.738267	-0.43779
37072_at	CNGB1	cyclic nucleotide gated channel beta 1	0.005006	0.612601	-0.70698
344_s_at	CNP	2',3'-cyclic nucleotide 3' phosphodiesterase	0.014925	0.620134	-0.68935
38420_at	COL5A2	collagen, type V, alpha 2	0.043456	1.33344	0.415151
39925_at	COL9A2	collagen, type IX, alpha 2	0.033891	0.530416	-0.9148
36455_at	COL9A3	collagen, type IX, alpha 3	0.012429	0.474626	-1.07514
33132_at	CPSF1	cleavage and polyadenylation specific factor 1, 160kDa	0.016661	0.546674	-0.87125
40844_at	CTR9	Ctr9, Paf1/RNA polymerase II complex component, homolog (S. cerevisiae)	0.043669	1.42451	0.510462
40646_at	CX3CR1	chemokine (C-X3-C motif) receptor 1	0.00945	0.506698	-0.9808
35329_at	CYB5R1	cytochrome b5 reductase 1	0.023589	1.59936	0.677496
33295_at	DARC	Duffy blood group, chemokine receptor	0.000781	1.56091	0.642385
886_at	DCK	deoxycytidine kinase	0.024274	1.31195	0.39171
38355_at	DDX3Y	DEAD (Asp-Glu-Ala-Asp) box polypeptide 3, Y-linked	0.039626	2.63525	1.39794
35403_at	DOLK	dolichol kinase	0.02151	0.634862	-0.65549
36699_at	DSCAM	Down syndrome cell adhesion molecule	0.042313	1.54136	0.624203
36831_at	DSTYK	dual serine/threonine and tyrosine protein kinase	0.038539	1.47622	0.561909
35347_at	EFEMP2	EGF-containing fibulin-like extracellular matrix protein 2	0.0112	1.33445	0.416251
1008_f_at	EIF2AK2	eukaryotic translation initiation factor 2-alpha kinase 2	0.042712	0.758104	-0.39953
41124_r_at	ENPP2	ectonucleotide pyrophosphatase/phosphodiesterase 2	0.007651	0.541054	-0.88616
41095_at	ERCC2	excision repair cross-complementing rodent repair deficiency, complementation group 2	0.015919	0.530584	-0.91435
1258_s_at	ERCC4	excision repair cross-complementing rodent repair deficiency, complementation group 4	0.000914	1.67367	0.743017
38996_at	ERF	Ets2 repressor factor	0.026384	0.650078	-0.62132
41686_s_at	FAM153A /// FAM153B /// LOC100507397	family with sequence similarity 153, member A /// family with sequence similarity 153, member B /// hypothetical LOC100507397	0.006208	1.35936	0.442925
34708_at	FCN3	ficolin (collagen/fibrinogen domain containing) 3 (Hakata antigen)	0.003338	1.66368	0.734374
35081_at	FGF9	fibroblast growth factor 9 (glia-activating factor)	0.012779	1.50614	0.590854
33556_at	FICD	FIC domain containing	0.034126	1.52929	0.612862
1740_g_at	FOLH1 /// FOLH1B	folate hydrolase (prostate-specific membrane antigen) 1 /// folate hydrolase 1B	0.004704	0.510899	-0.96889
35296_at	GGPS1	geranylgeranyl diphosphate synthase 1	0.020886	1.34934	0.432257
34297_at	GPR37	G protein-coupled receptor 37 (endothelin receptor type B-like)	0.039484	0.450192	-1.15139
40240_at	GPRC5B	G protein-coupled receptor, family C, group 5, member B	0.047235	0.688149	-0.53921
38236_at	GRIN2A	glutamate receptor, ionotropic, N-methyl D-aspartate 2A	0.044381	0.677926	-0.5608
1135_at	GRK5	G protein-coupled receptor kinase 5	0.017496	1.46882	0.554659
1253_at	GSK3B	glycogen synthase kinase 3 beta	0.022399	1.61694	0.693268
32612_at	GSN	gelsolin	0.043893	0.668128	-0.5818
375_at	GSTT1	glutathione S-transferase theta 1	0.044792	1.4521	0.538144
35334_at	GYG2	glycogenin 2	0.014293	1.68708	0.754525
34308_at	HIST1H2AC	histone cluster 1, H2ac	0.031901	1.5343	0.617579
40778_at	HSD17B10	hydroxysteroid (17-beta) dehydrogenase 10	0.045649	0.631378	-0.66342
645_at	HSPA2	heat shock 70kDa protein 2	0.005908	0.302021	-1.72728
33550_at	HTR2C	5-hydroxytryptamine (serotonin) receptor 2C	0.032814	0.701419	-0.51165

36875_at	IBTK	inhibitor of Bruton agammaglobulinemia tyrosine kinase	0.047162	1.56482	0.645997
38453_at	ICAM2	intercellular adhesion molecule 2	0.007627	0.766096	-0.3844
41049_at	IRS1	insulin receptor substrate 1	0.036937	1.33065	0.412133
1895_at	JUN	jun proto-oncogene	0.018656	0.763048	-0.39016
41484_r_at	JUND	jun D proto-oncogene	0.048354	0.437291	-1.19333
41589_at	KCNQ2	potassium voltage-gated channel, KQT-like subfamily, member 2	0.001984	1.3467	0.429431
37387_r_at	KDELR1	KDEL (Lys-Asp-Glu-Leu) endoplasmic reticulum protein retention receptor 1	0.0026	0.661036	-0.5972
36548_at	KIAA0895	KIAA0895	0.045762	0.747591	-0.41968
36070_at	KIAA1199	KIAA1199	0.006699	0.523008	-0.93509
32079_at	KIF13B	kinesin family member 13B	0.035782	0.660562	-0.59823
40407_at	KPNA2	karyopherin alpha 2 (RAG cohort 1, importin alpha 1)	0.011978	1.3581	0.441585
34031_i_at	KRIT1	KRIT1, ankyrin repeat containing	0.041336	1.35364	0.436841
36917_at	LAMA2	laminin, alpha 2	0.049095	1.37483	0.459258
38402_at	LAMP2	lysosomal-associated membrane protein 2	0.049158	0.703683	-0.507
35367_at	LGALS3	lectin, galactoside-binding, soluble, 3	0.010317	1.85702	0.892986
38745_at	LIPA	lipase A, lysosomal acid, cholesterol esterase	0.031404	0.638626	-0.64696
34387_at	LPGAT1	lysophosphatidylglycerol acyltransferase 1	0.015123	0.677589	-0.56152
41209_at	LPL	lipoprotein lipase	0.018186	1.42912	0.515126
36711_at	MAFF	v-maf musculoaponeurotic fibrosarcoma oncogene homolog F (avian)	0.032142	2.129	1.09018
38558_at	MAG	myelin associated glycoprotein	0.012734	0.576021	-0.79581
38727_at	MCFD2	multiple coagulation factor deficiency 2	0.028718	1.37732	0.461866
31824_at	ME1	malic enzyme 1, NADP(+)-dependent, cytosolic	0.03123	1.49498	0.580124
37712_g_at	MEF2C	myocyte enhancer factor 2C	0.003579	2.20111	1.13823
38499_s_at	MOBP	myelin-associated oligodendrocyte basic protein	0.04579	0.517791	-0.94956
39120_at	MT1X	metallothionein 1X	0.009844	1.68724	0.754667
39607_at	MTMR9	myotubularin related protein 9	0.017617	1.83414	0.875101
41055_at	NACAD	NAC alpha domain containing	0.0361	0.644119	-0.6346
36977_at	NAPA	N-ethylmaleimide-sensitive factor attachment protein, alpha	0.043524	0.707455	-0.49929
38557_at	NCAM2	neural cell adhesion molecule 2	0.004698	1.62503	0.700462
39075_at	NEU1	sialidase 1 (lysosomal sialidase)	0.04194	0.630192	-0.66614
36868_at	NFASC	neurofascin	0.027062	0.755293	-0.40489
1979_s_at	NOP2	NOP2 nucleolar protein homolog (yeast)	0.020394	0.616442	-0.69796
37047_at	NPC1	Niemann-Pick disease, type C1	0.019494	0.634838	-0.65554
38604_at	NPY	neuropeptide Y	0.029619	0.674484	-0.56814
37623_at	NR4A2	nuclear receptor subfamily 4, group A, member 2	0.013068	0.533038	-0.90769
738_at	NT5C2	5'-nucleotidase, cytosolic II	0.012054	1.45213	0.538175
36423_at	NUPR1	nuclear protein, transcriptional regulator, 1	0.039851	1.70906	0.773204
38312_at	OLFML2A	olfactomedin-like 2A	0.027796	1.38103	0.465748
40624_at	OLIG2	oligodendrocyte lineage transcription factor 2	0.017591	0.688231	-0.53904
32474_at	PAX7	paired box 7	0.037044	0.675312	-0.56638
32696_at	PBX3	pre-B-cell leukemia homeobox 3	0.044748	1.36041	0.444039
37676_at	PDE8A	phosphodiesterase 8A	0.003853	0.410238	-1.28547
37249_at	PDE8B	phosphodiesterase 8B	0.003202	1.32527	0.406283
1771_s_at	PDGFRB	platelet-derived growth factor receptor, beta polypeptide	0.036059	1.33493	0.416764
40471_at	PEX19	peroxisomal biogenesis factor 19	0.007308	1.31789	0.398234
35719_at	PHLPP1	PH domain and leucine rich repeat protein phosphatase 1	0.021298	0.599722	-0.73764
32558_at	PIAS3	protein inhibitor of activated STAT, 3	0.007465	1.52238	0.606328
37901_at	PIK3R4	phosphoinositide-3-kinase, regulatory subunit 4	0.021633	0.658798	-0.60209
199_s_at	PKN2	protein kinase N2	0.048427	1.47896	0.564584
41689_at	PLLP	plasmolipin	0.003758	0.533112	-0.90749
41158_at	PLP1	proteolipid protein 1	0.028838	0.707968	-0.49824
38653_at	PMP22	peripheral myelin protein 22	0.039826	0.682399	-0.55131

32310_f_at	PMS2P1	postmeiotic segregation increased 2 pseudogene 1	0.036849	0.768602	-0.37969
35173_at	PNPLA4	patatin-like phospholipase domain containing 4	0.00614	1.66618	0.736548
1470_at	POLD2	polymerase (DNA directed), delta 2, regulatory subunit 50kDa	0.036522	1.44578	0.531852
37028_at	PPP1R15A	protein phosphatase 1, regulatory (inhibitor) subunit 15A	0.012086	0.481103	-1.05558
36898_r_at	PRIM2	primase, DNA, polypeptide 2 (58kDa)	0.001728	1.5388	0.621802
32784_at	PRPF4B	PRP4 pre-mRNA processing factor 4 homolog B (yeast)	0.001634	1.47808	0.563723
994_at	PTPRM	protein tyrosine phosphatase, receptor type, M	0.046583	1.4487	0.534759
34320_at	PTRF	polymerase I and transcript release factor	0.037056	0.694492	-0.52597
39327_at	PXDN	peroxidasin homolog (Drosophila)	0.029138	1.40603	0.491623
39760_at	QKI	quaking homolog, KH domain RNA binding (mouse)	0.033776	0.750713	-0.41367
32741_at	RABEP1	rabaptin, RAB GTPase binding effector protein 1	0.04028	1.33351	0.415225
39253_s_at	RALA	v-ral simian leukemia viral oncogene homolog A (ras related)	0.039272	1.50155	0.586449
1848_at	RAP1A	RAP1A, member of RAS oncogene family	0.034275	0.768959	-0.37902
40556_at	RCN1	reticulocalbin 1, EF-hand calcium binding domain	0.015817	1.54626	0.628785
37651_at	RCOR1	REST corepressor 1	0.044074	1.5214	0.605395
36034_at	RGP1	RGP1 retrograde golgi transport homolog (S. cerevisiae)	0.017223	1.4115	0.49723
34268_at	RGS19	regulator of G-protein signaling 19	0.029572	1.58595	0.665348
1826_at	RHOB	ras homolog gene family, member B	0.040304	0.56199	-0.83139
37402_at	RNASE1	ribonuclease, RNase A family, 1 (pancreatic)	0.018101	0.484317	-1.04598
32036_i_at	RPP14	ribonuclease P/MRP 14kDa subunit	0.009399	2.07797	1.05517
35195_at	RTCD1	RNA terminal phosphate cyclase domain 1	0.007561	1.53059	0.614089
587_at	S1PR1	sphingosine-1-phosphate receptor 1	0.002122	1.5048	0.589574
35827_at	SEC31A	SEC31 homolog A (S. cerevisiae)	0.042443	1.35042	0.433407
38820_at	SEP15	15 kDa selenoprotein	0.01509	1.33397	0.415725
34363_at	SEPP1	selenoprotein P, plasma, 1	0.011957	0.602004	-0.73216
38033_at	SEPT10	septin 10	0.047482	0.700713	-0.5131
33173_g_at	SEPT11	septin 11	0.012475	1.5704	0.651131
34840_at	SERINC5	serine incorporator 5	0.023804	0.71867	-0.4766
39444_at	SF3B1	splicing factor 3b, subunit 1, 155kDa	0.000923	1.35114	0.434178
39825_at	SLC25A1	"solute carrier family 25 (mitochondrial carrier; citrate transporter), member 1"	0.013383	0.648771	-0.62422
38208_at	SLC35A3	solute carrier family 35 (UDP-N-acetylglucosamine (UDP-GlcNAc) transporter), member A3	0.033292	0.735198	-0.4438
37045_at	SNX19	sorting nexin 19	0.010437	0.517541	-0.95026
36018_at	SOX10	SRY (sex determining region Y)-box 10	0.012109	0.528839	-0.9191
37353_g_at	SP100	SP100 nuclear antigen	0.025305	1.44483	0.530904
33596_at	SPOCK3	sparc/osteonectin, cwcv and kazal-like domains proteoglycan (testican) 3	0.021836	0.539788	-0.88954
32227_at	SRGN	serglycin	0.016146	1.71366	0.777085
34717_s_at	SRSF10	serine/arginine-rich splicing factor 10	0.043811	0.665712	-0.58703
37782_at	SST	somatostatin	0.025779	0.562182	-0.83089
39391_at	STAMBP	STAM binding protein	0.048761	0.704357	-0.50562
37182_at	SYP	synaptophysin	0.047764	0.703847	-0.50667
32048_at	TCEB3	Transcription elongation factor B (SIII), polypeptide 3 (110kDa, elongin A)	0.034949	1.58141	0.66121
38966_at	TECR	trans-2,3-enoyl-CoA reductase	0.03233	0.70532	-0.50365
32538_at	TF	transferrin	0.029317	0.523262	-0.9344
659_g_at	THBS2	thrombospondin 2	0.038476	0.569319	-0.81269
40601_at	TM2D1	TM2 domain containing 1	0.019258	1.73978	0.798903
37445_at	TMEM5	transmembrane protein 5	0.022173	1.31479	0.394833
40437_at	TMEM87A	transmembrane protein 87A	0.020919	0.588474	-0.76495
1563_s_at	TNFRSF1A	tumor necrosis factor receptor superfamily, member 1A	0.019195	1.52836	0.611983
32244_at	TOX4	TOX high mobility group box family	0.001084	1.45893	0.544913

		member 4			
32824_at	TPP1	tripeptidyl peptidase I	0.008211	1.33483	0.416659
32599_at	TSC1	tuberous sclerosis 1	0.020403	1.31865	0.399065
38813_at	TSC2	tuberous sclerosis 2	0.025912	0.722414	-0.4691
41788_i_at	TSC22D2	TSC22 domain family, member 2	0.03303	1.4166	0.502434
36083_at	TSPAN31	tetraspanin 31	0.007668	1.30263	0.381424
379_at	TXNDC9	thioredoxin domain containing 9	0.035119	1.51319	0.597593
33631_at	TXNL4A	thioredoxin-like 4A	0.037654	1.44395	0.530017
40619_at	UBE2S	ubiquitin-conjugating enzyme E2S	0.017721	0.689044	-0.53733
37244_at	UCHL3	ubiquitin carboxyl-terminal esterase L3 (ubiquitin thiolesterase)	0.029963	1.50266	0.587522
35722_at	UPF2	UPF2 regulator of nonsense transcripts homolog (yeast)	0.01244	1.45967	0.545645
34477_at	UTY	ubiquitously transcribed tetratricopeptide repeat gene, Y-linked	0.014473	0.648062	-0.6258
39313_at	WNK1	WNK lysine deficient protein kinase 1	0.029975	1.55114	0.633326
40928_at	WSB1	WD repeat and SOCS box-containing 1	0.025366	1.43231	0.518347
34613_at	ZFR2	zinc finger RNA binding protein 2	0.019334	1.57042	0.651152
36957_at	ZMYND8	zinc finger, MYND-type containing 8	0.008759	1.38169	0.466438
36048_at	ZNF318	zinc finger protein 318	0.002153	1.98634	0.990112
AFFX-BioDn-3_at	---	---	0.034896	1.36343	0.447239
31655_at	---	---	0.036592	0.69272	-0.52966
34112_r_at	---	---	0.027553	1.44951	0.53557
41247_at	---	---	0.02508	1.36906	0.453191

Table 2: DE genes and probesets, occurring from comparison of BD and control gene expression profiles. In the case that different probesets correspond to the same gene symbol, the one depicting the higher fold change is shown.

Probe Set ID	Gene Symbol	Gene Title	p-value	Fold Change (natural) BD vs CONTROL	Fold Change (log2) BD vs CONTROL
31519_f_at	---	---	0.020069	1.49886	0.58387
40628_at	AAK1	AP2 associated kinase 1	0.028355	1.35125	0.43429
39408_at	ACADS	acyl-CoA dehydrogenase, C-2 to C-3 short chain	0.015489	1.49261	0.577834
36269_at	ADAMTS3	ADAM metallopeptidase with thrombospondin type 1 motif, 3	0.003084	0.742579	-0.42938
36051_s_at	ADD2	adducin 2 (beta)	0.013917	1.44368	0.529755
32676_at	ALDH6A1	aldehyde dehydrogenase 6 family, member A1	0.022513	1.36666	0.450652
38068_at	AMFR	autocrine motility factor receptor	0.031309	0.628455	-0.67012
40793_s_at	AQP4	aquaporin 4	0.013569	1.67244	0.741952
37041_at	ATG4B	ATG4 autophagy related 4 homolog B (S. cerevisiae)	0.020851	0.514742	-0.95808
35670_at	ATP1A3	ATPase, Na+/K+ transporting, alpha 3 polypeptide	0.04354	0.705374	-0.50354
201_s_at	B2M	beta-2-microglobulin	0.021785	1.46159	0.54754
1801_at	BARD1	BRCA1 associated RING domain 1	0.039949	1.43308	0.519114
32606_at	BASP1	Brain abundant, membrane attached signal protein 1	0.020374	0.716708	-0.48054
38126_at	BGN	biglycan	0.025364	1.35707	0.4405
40483_at	C1orf61	chromosome 1 open reading frame 61	0.039991	1.99014	0.992868
482_at	CDH13	cadherin 13, H-cadherin (heart)	0.045569	1.3213	0.401955
1052_s_at	CEBPD	CCAAT/enhancer binding protein (C/EBP), delta	0.015057	1.65432	0.726239
36514_at	CGRRF1	cell growth regulator with ring finger domain 1	0.04213	1.35992	0.443519
450_g_at	CLTB	cell growth regulator with ring finger domain 1	0.021067	1.45595	0.541959
32523_at	CREB3L2	clathrin, light chain B	0.029544	0.749715	-0.41559
39692_at	CREBL2	cAMP responsive element binding protein 3-like 2	0.025841	1.62064	0.69656
39438_at	CRK	cAMP responsive element binding protein-like 2	0.031157	0.69706	-0.52065

38219_at	CSDA	v-crk sarcoma virus CT10 oncogene homolog (avian)	0.005117	1.74852	0.806132
39839_at	CUL9	cold shock domain protein A	0.020743	1.55565	0.63752
41614_at	DAGLA	cullin 9	0.000612	0.738689	-0.43696
35511_at	DARC	diacylglycerol lipase, alpha	0.028906	0.594247	-0.75087
33295_at	DENND4B	Duffy blood group, chemokine receptor	0.010586	1.41302	0.498777
35786_at	DHRS3	DENN/MADD domain containing 4B	0.011679	0.768238	-0.38037
40782_at	DHX9	dehydrogenase/reductase (SDR family) member 3	0.048213	1.36033	0.443952
662_at	DIXDC1	DEAH (Asp-Glu-Ala-His) box polypeptide 9	0.044626	1.63647	0.710583
36534_at	DLGAP4	DIX domain containing 1	0.034598	1.62818	0.703257
41134_at	DLST	discs, large (Drosophila) homolog-associated protein 4	0.029158	0.717346	-0.47926
33258_g_at	DOPEY1	dihydrolipoamide S-succinyltransferase (E2 component of 2-oxo-glutarate complex)	0.03187	1.92012	0.941195
40612_at	DTNA	dopey family member 1	0.001795	0.61183	-0.7088
36466_at	DZIP3	dystrobrevin, alpha	0.017555	1.45497	0.540994
32131_at	EIF4EBP2	DAZ interacting protein 3, zinc finger	0.02654	0.664625	-0.58939
35263_at	EP400	eukaryotic translation initiation factor 4E binding protein 2	0.013857	1.36376	0.447589
34816_at	EXPH5	E1A binding protein p400	0.03323	0.709537	-0.49505
39625_at	FAAH	exophilin 5	0.037266	1.35247	0.4356
34769_at	FRY	fatty acid amide hydrolase	0.048576	0.72736	-0.45926
1530_g_at	GMPR	furry homolog (Drosophila)	0.039696	0.681458	-0.5533
31812_at	GPR162	guanosine monophosphate reductase	0.001324	1.3864	0.471341
37942_at	GRK5	G protein-coupled receptor 162	0.032128	0.630931	-0.66445
40994_at	HDDC2	G protein-coupled receptor kinase 5	0.025371	0.662812	-0.59333
34359_at	HERC2P2 /// HERC2P9	HD domain containing 2	0.011186	1.3851	0.469986
40877_s_at	HIST1H2BK	hect domain and RLD 2 pseudogene 2 /// hect domain and RLD 2 pseudogene 9	0.013856	0.720544	-0.47284
32819_at	HPS5	histone cluster 1, H2bk	0.022815	1.62648	0.701757
35223_at	HTR2C	Hermansky-Pudlak syndrome 5	0.044432	1.5012	0.586112
33550_at	IFITM3	5-hydroxytryptamine (serotonin) receptor 2C	0.03028	0.699364	-0.51588
41745_at	IL27RA	interferon induced transmembrane protein 3 (1-8U)	0.005408	1.6362	0.710348
37844_at	ING3	interleukin 27 receptor, alpha	0.025291	0.634555	-0.65618
31808_at	IQSEC3	inhibitor of growth family, member 3	0.0137	1.41078	0.496488
34250_at	KCNK1	IQ motif and Sec7 domain 3	0.048318	0.74944	-0.41612
37552_at	KCNK3	potassium channel, subfamily K, member 1	0.010237	0.72612	-0.46172
41325_at	KDR	potassium channel, subfamily K, member 3	0.009795	0.627401	-0.67254
1954_at	KIAA0141	kinase insert domain receptor (a type III receptor tyrosine kinase)	0.021625	1.73194	0.792388
35744_at	LAMB2	KIAA0141	0.047761	1.47686	0.562538
38812_at	LARP4	laminin, beta 2 (laminin S)	0.013242	1.48061	0.566194
35180_at	LGALS3	La ribonucleoprotein domain family, member 4	0.014345	1.31809	0.398453
35367_at	LRP4	lectin, galactoside-binding, soluble, 3	0.038661	1.55458	0.636527
36059_at	MAF	low density lipoprotein receptor-related protein 4	0.036948	1.43165	0.51768
41505_r_at	MAPT	v-maf musculoaponeurotic fibrosarcoma oncogene homolog (avian)	0.049097	0.728387	-0.45722
310_s_at	MINK1	microtubule-associated protein tau	0.023843	0.570673	-0.80926
39833_at	MT1E	misshapen-like kinase 1	0.020735	0.736928	-0.44041
36130_f_at	MT1H	metallothionein 1E	0.004131	1.60681	0.684202
39594_f_at	MT1X	metallothionein 1H	0.032186	1.3572	0.440635
39120_at	MYL12A	metallothionein 1X	0.00247	1.95363	0.966154

33447_at	NFIC	myosin, light chain 12A, regulatory, non-sarcomeric	0.036581	1.33687	0.418857
440_at	NME1	nuclear factor I/C (CCAAT-binding transcription factor)	0.008999	0.683786	-0.54838
39073_at	NME5	non-metastatic cells 1, protein (NM23A) expressed in	0.015109	0.714204	-0.48559
36859_at	NOP56	non-metastatic cells 5, protein expressed in (nucleoside-diphosphate kinase)	0.030115	0.669277	-0.57933
34882_at	NPRL2	NOP56 ribonucleoprotein homolog (yeast)	0.01145	1.59671	0.675101
40497_at	NTRK3	nitrogen permease regulator-like 2 (S. cerevisiae)	0.037159	0.620251	-0.68908
1059_at	NUP133	neurotrophic tyrosine kinase, receptor, type 3	0.007457	0.641933	-0.63951
1060_g_at	ODC1	neurotrophic tyrosine kinase, receptor, type 3	0.020803	0.548371	-0.86678
39390_at	PARVB	nucleoporin 133kDa	0.039775	0.732228	-0.44964
36203_at	PDE6D	ornithine decarboxylase 1	0.000296	1.39444	0.479686
37965_at	PFKFB2	parvin, beta	0.001087	0.590989	-0.7588
36044_at	POLR2K	phosphodiesterase 6D, cGMP-specific, rod, delta	0.004032	1.36911	0.453234
34108_g_at	POLR3C	6-phosphofructo-2-kinase/fructose-2,6-biphosphatase 2	0.039145	1.47794	0.563585
39766_r_at	PPFIA4	polymerase (RNA) II (DNA directed) polypeptide K, 7.0kDa	0.012146	1.43083	0.516856
782_at	PPIG	polymerase (RNA) III (DNA directed) polypeptide C (62kD)	0.041025	0.664184	-0.59035
38167_at	PRKDC	protein tyrosine phosphatase, receptor type, f polypeptide (PTPRF), interacting protein (liprin), alpha 4	0.020483	0.769116	-0.37873
37385_at	PRR4	peptidylprolyl isomerase G (cyclophilin G)	0.032875	1.33883	0.420968
1250_at	PYROXD1	protein kinase, DNA-activated, catalytic polypeptide	0.042783	0.660297	-0.59881
2012_s_at	RBM25	protein kinase, DNA-activated, catalytic polypeptide	0.035105	0.718301	-0.47734
36024_at	RBM5	proline rich 4 (lacrimal)	0.030737	0.704103	-0.50614
34685_at	RELA	pyridine nucleotide-disulphide oxidoreductase domain 1	0.015488	0.743713	-0.42718
41208_at	RFC4	RNA binding motif protein 25	0.007671	1.62373	0.699315
1556_at	RFTN1	RNA binding motif protein 5	0.030662	0.75502	-0.40541
36645_at	RNASEH2B	v-rel reticuloendotheliosis viral oncogene homolog A (avian)	0.014752	1.43892	0.524991
1055_g_at	RNF113A	replication factor C (activator 1) 4, 37kDa	0.002467	1.72942	0.790288
32593_at	RPL10A	raftlin, lipid raft linker 1	0.020381	0.708534	-0.49709
35972_at	RPP40	ribonuclease H2, subunit B	0.046123	0.644359	-0.63406
36565_at	RPS15A	ring finger protein 113A	0.011435	1.62455	0.700042
36786_at	RPS16	ribosomal protein L10a	0.013277	1.36824	0.452326
37471_at	RPS19	ribonuclease P/MRP 40kDa subunit	0.031148	1.47616	0.561852
34317_g_at	RPS2	ribosomal protein S15a	0.00383	1.3233	0.40414
34316_at	RPS28	ribosomal protein S15a	0.001177	1.47855	0.564187
38061_at	RPS5	ribosomal protein S16	0.002093	1.36774	0.451798
31330_at	RRP15	ribosomal protein S19	0.008103	1.32712	0.408299
31527_at	SDC4	ribosomal protein S2	0.001826	1.32273	0.403522
39798_at	SEC62	ribosomal protein S28	0.021034	1.32205	0.402776
32437_at	SEPT11	ribosomal protein S5	0.00134	1.35424	0.437487
36360_at	SERINC5	ribosomal RNA processing 15 homolog (S. cerevisiae)	0.013528	1.77077	0.824375
35844_at	SF3B1	syndecan 4	0.01945	2.07693	1.05445
950_at	SLC12A5	SEC62 homolog (S. cerevisiae)	0.009231	1.31977	0.400283
35181_at	SLC25A1	septin 11	0.027838	0.68245	-0.5512
34840_at	SLC7A8	serine incorporator 5	0.026253	0.676158	-0.56457
39444_at	SMPDL3A	splicing factor 3b, subunit 1, 155kDa	0.01175	1.35219	0.435295
34208_at	SNRPD2	solute carrier family 12 (potassium/chloride transporter), member 5	0.033666	0.669071	-0.57977

38997_at	SPARC	"solute carrier family 25 (mitochondrial carrier; citrate transporter), member 1"	0.007457	0.647509	-0.62703
41271_at	SPTBN2	solute carrier family 7 (amino acid transporter, L-type), member 8	0.01973	0.745799	-0.42314
39950_at	SQLE	sphingomyelin phosphodiesterase, acid-like 3A	0.035423	0.698029	-0.51864
35270_at	STAT6	small nuclear ribonucleoprotein D2 polypeptide 16.5kDa	0.033748	1.33661	0.418581
671_at	STOM	secreted protein, acidic, cysteine-rich (osteonectin)	0.026794	1.40826	0.493911
33630_s_at	SYP	spectrin, beta, non-erythrocytic 2	0.004662	0.681248	-0.55375
35839_at	TIMP3	squalene epoxidase	0.023152	0.660121	-0.5992
41222_at	TRIO	signal transducer and activator of transcription 6, interleukin-4 induced	0.03153	0.720249	-0.47343
40419_at	TRIP13	stomatin	0.017451	1.34123	0.423552
37182_at	TRPC1	synaptophysin	0.033657	0.639796	-0.64432
1035_g_at	TUBB2B	TIMP metallopeptidase inhibitor 3	0.016964	1.64463	0.717766
40792_s_at	TXNIP	triple functional domain (PTPRF interacting)	0.005374	0.746866	-0.42108
36813_at	UBE2D2	thyroid hormone receptor interactor 13	0.000328	0.720069	-0.47379
39123_s_at	UCHL3	transient receptor potential cation channel, subfamily C, member 1	0.02989	0.598806	-0.73984
39332_at	YBX1	tubulin, beta 2B	0.043949	1.3054	0.384493
31508_at	YWHAE	thioredoxin interacting protein	0.006163	1.76301	0.818041
832_at	ZMYM4	ubiquitin-conjugating enzyme E2D 2 (UBC4/5 homolog, yeast)	0.007123	1.61613	0.692546
37244_at	ZNF204P	ubiquitin carboxyl-terminal esterase L3 (ubiquitin thiolesterase)	0.021652	1.62671	0.701958
32340_s_at		Y box binding protein 1	0.010721	1.45549	0.541503
1011_s_at		tyrosine 3-monooxygenase/tryptophan 5-monooxygenase activation protein, epsilon polypeptide	0.013936	0.661326	-0.59657
39762_at		zinc finger, MYM-type 4	0.008846	0.729522	-0.45498
34001_at		zinc finger protein 204, pseudogene	0.004382	0.662771	-0.59342
326_i_at	---	---	0.017059	1.41523	0.501038

<u>STUDY2</u>

Table 3. DE genes and probesets, occurring from comparison of BD and control gene expression profiles. In the case that different probesets correspond to the same gene symbol, the one depicting the higher fold change is shown.

Probe Set ID	Gene Symbol	Gene Title	p-value	Fold Change (natural) BD vs CONTROL	Fold Change (log2) BD vs cONTROL
212502_at		2-aminoethanethiol dioxygenase	0.007233	0.576418	-0.79481
202170_s_at	AASDHPPT	aminoadipate-semialdehyde dehydrogenase-phosphopantetheinyl transferase	0.00504	0.588629	-0.76457
210100_s_at	ABCA2	ATP-binding cassette, sub-family A (ABC1), member 2	0.04979	0.324067	-1.62564
204617_s_at	ACD	adrenocortical dysplasia homolog (mouse)	0.004302	2.37261	1.24647
201128_s_at	ACLY	ATP citrate lyase	0.007614	0.738222	-0.43787
201662_s_at	ACSL3	acyl-CoA synthetase long-chain family member 3	0.028528	0.733247	-0.44763
202422_s_at	ACSL4	acyl-CoA synthetase long-chain family member 4	0.003487	0.520745	-0.94135
212984_at	activating transcription factor 2	chr2:175936986-175938895 (-) // 90.99 // q31.1	0.004824	0.481079	-1.05565
222230_s_at	ACTR10	actin-related protein 10 homolog (S. cerevisiae)	0.025871	0.709235	-0.49567

200729_s_at	ACTR2	ARP2 actin-related protein 2 homolog (yeast)	0.01626	0.608614	-0.7164
200996_at	ACTR3	ARP3 actin-related protein 3 homolog (yeast)	0.011105	0.703695	-0.50698
213808_at	ADAM23	ADAM metallopeptidase domain 23	0.012946	0.544529	-0.87692
202381_at	ADAM9	ADAM metallopeptidase domain 9	0.022402	0.676504	-0.56383
213245_at	ADCY1	adenylate cyclase 1 (brain)	0.025459	0.667025	-0.58419
203741_s_at	ADCY7	adenylate cyclase 7	0.001882	0.469888	-1.08961
206811_at	ADCY8	adenylate cyclase 8 (brain)	0.048564	1.33347	0.415191
200760_s_at	ADP-ribosylation-like factor 6 interacting protein 5	chr3:69134093-69155217 (+) // 96.46 // p14.1	0.002391	0.664038	-0.59066
221761_at	ADSS	adenylosuccinate synthase	0.001597	0.661224	-0.59679
204066_s_at	AGAP1	ArfGAP with GTPase domain, ankyrin repeat and PH domain 1	0.005524	0.547677	-0.8686
218092_s_at	AGFG1	ArfGAP with FG repeats 1	0.001777	0.625392	-0.67717
218534_s_at	AGGF1	angiogenic factor with G patch and FHA domains 1	0.007019	0.667893	-0.58231
204500_s_at	AGTPBP1	ATP/GTP binding protein 1	0.016823	0.600151	-0.7366
221008_s_at	AGXT2L1	alanine-glyoxylate aminotransferase 2-like 1	0.033643	1.97157	0.979342
212172_at	AK2	adenylate kinase 2	0.01334	1.5898	0.668844
201675_at	AKAP1	A kinase (PRKA) anchor protein 1	0.018592	0.698199	-0.51829
203156_at	AKAP11	A kinase (PRKA) anchor protein 11	0.007539	0.735756	-0.4427
209160_at	AKR1C3	aldo-keto reductase family 1, member C3 (3-alpha hydroxysteroid dehydrogenase, type II)	0.031555	1.90832	0.932301
201951_at	ALCAM	activated leukocyte cell adhesion molecule	0.016351	0.535521	-0.90099
202022_at	ALDOC	aldolase C, fructose-bisphosphate	0.025523	1.3945	0.479746
201197_at	AMD1	adenosylmethionine decarboxylase 1	0.025017	0.655	-0.61043
204294_at	AMT	aminomethyltransferase	0.006807	1.4622	0.548139
208353_x_at	ANK1	ankyrin 1, erythrocytic	0.003503	1.45958	0.545551
214919_s_at	ANKHD1-EIF4EBP3 /// EIF4EBP3	ANKHD1-EIF4EBP3 readthrough /// eukaryotic translation initiation factor 4E binding protein 3	0.027731	1.37026	0.454448
216073_at	ANKRD34C	ankyrin repeat domain 34C	0.020538	0.451323	-1.14777
212731_at	ANKRD46	ankyrin repeat domain 46	0.002045	0.671881	-0.57372
209442_x_at	ankyrin 3, node of Ranvier (ankyrin G)	chr10:61789457-61900433 (-) // 99.51 // q21.2	0.016675	0.688873	-0.53769
218804_at	ANO1	anoctamin 1, calcium activated chloride channel	0.020201	1.63617	0.710322
203300_x_at	AP1S2	adaptor-related protein complex 1, sigma 2 subunit	0.031783	0.642347	-0.63858
210278_s_at	AP4S1	adaptor-related protein complex 4, sigma 1 subunit	0.043969	0.558228	-0.84107
215148_s_at	APBA3	amyloid beta (A4) precursor protein-binding, family A, member 3	0.021744	1.99085	0.993388
201686_x_at	API5	apoptosis inhibitor 5	0.013796	0.619853	-0.69
203382_s_at	APOE	apolipoprotein E	0.018525	1.75855	0.814386
221620_s_at	APOO	apolipoprotein O	0.036479	0.739943	-0.43451
213289_at	APOOL	apolipoprotein O-like	0.005534	0.635048	-0.65506
211277_x_at	APP	amyloid beta (A4) precursor protein	0.021895	0.519586	-0.94457
218158_s_at	APPL1	adaptor protein, phosphotyrosine interaction, PH domain and leucine zipper containing 1	0.005932	0.611556	-0.70944
34206_at	ARAP1	ArfGAP with RhoGAP domain, ankyrin repeat and PH domain 1	0.02249	1.33056	0.412032
218098_at	ARFGEF2	ADP-ribosylation factor guanine nucleotide-exchange factor 2 (brefeldin A-inhibited)	0.006483	0.672127	-0.57319
203174_s_at	ARFRP1	ADP-ribosylation factor related protein 1	0.010088	3.83162	1.93796
203756_at	ARHGEF17	Rho guanine nucleotide exchange factor (GEF) 17	0.006981	1.44609	0.532156
209435_s_at	ARHGEF2	Rho/Rac guanine nucleotide exchange factor (GEF) 2	0.012715	0.716938	-0.48008
218501_at	ARHGEF3	Rho guanine nucleotide exchange factor (GEF) 3	0.0122	0.638146	-0.64804
205062_x_at	ARID4A	AT rich interactive domain 4A (RBP1-like)	0.005404	0.551575	-0.85837

221230_s_at	ARID4B	AT rich interactive domain 4B (RBP1-like)	0.045971	0.762611	-0.39098
201657_at	ARL1	ADP-ribosylation factor-like 1	0.010792	0.601247	-0.73397
202641_at	ARL3	ADP-ribosylation factor-like 3	0.011327	0.70959	-0.49494
202206_at	ARL4C	ADP-ribosylation factor-like 4C	0.0213	0.534461	-0.90384
218150_at	ARL5A	ADP-ribosylation factor-like 5A	0.007451	0.588256	-0.76549
220597_s_at	ARL6IP4	ADP-ribosylation-like factor 6 interacting protein 4	0.03473	1.449	0.535057
218185_s_at	ARMC1	armadillo repeat containing 1	0.000417	0.548011	-0.86772
219094_at	ARMC8	armadillo repeat containing 8	0.001789	0.384419	-1.37925
217858_s_at	ARMCX3	armadillo repeat containing, X-linked 3	0.000235	0.612153	-0.70804
208679_s_at	ARPC2	actin related protein 2/3 complex, subunit 2, 34kDa	0.024604	0.732393	-0.44931
208736_at	ARPC3	actin related protein 2/3 complex, subunit 3, 21kDa	0.025741	0.747305	-0.42023
211963_s_at	ARPC5	actin related protein 2/3 complex, subunit 5, 16kDa	0.015371	0.767685	-0.38141
221482_s_at	ARPP19	cAMP-regulated phosphoprotein, 19kDa	0.013898	0.717437	-0.47908
207919_at	ART1	ADP-ribosyltransferase 1	0.012732	1.53577	0.618963
205673_s_at	ASB9	ankyrin repeat and SOCS box-containing 9	0.00766	1.46626	0.552139
218782_s_at	ATAD2	ATPase family, AAA domain containing 2	0.003734	2.04212	1.03007
222103_at	ATF1	activating transcription factor 1	0.021831	0.54116	-0.88587
201242_s_at	ATP1B1	ATPase, Na+/K+ transporting, beta 1 polypeptide	0.032644	0.63394	-0.65758
215716_s_at	ATP2B1	ATPase, Ca++ transporting, plasma membrane 1	0.003527	0.552261	-0.85658
216120_s_at	ATP2B2	ATPase, Ca++ transporting, plasma membrane 2	0.002083	0.51012	-0.97109
207507_s_at	ATP5G3	ATP synthase, H+ transporting, mitochondrial Fo complex, subunit C3 (subunit 9)	0.002741	0.741262	-0.43195
201443_s_at	ATP6AP2	ATPase, H+ transporting, lysosomal accessory protein 2	0.000427	0.705074	-0.50415
201972_at	ATP6V1A	ATPase, H+ transporting, lysosomal 70kDa, V1 subunit A	0.001518	0.6755	-0.56597
208899_x_at	ATP6V1D	ATPase, H+ transporting, lysosomal 34kDa, V1 subunit D	0.023958	0.638259	-0.64779
214762_at	ATP6V1G2	ATPase, H+ transporting, lysosomal 13kDa, V1 subunit G2	0.042759	0.677403	-0.56191
219659_at	ATP8A2	ATPase, aminophospholipid transporter, class I, type 8A, member 2	0.047708	0.617042	-0.69656
208833_s_at	ATXN10	ataxin 10	0.036334	0.751634	-0.4119
205052_at	AUH	AU RNA binding protein/enoyl-CoA hydratase	0.002168	0.692894	-0.52929
220525_s_at	AUP1	ancient ubiquitous protein 1	0.001268	1.61926	0.695333
202686_s_at	AXL	AXL receptor tyrosine kinase	0.022843	1.69407	0.760497
201772_at	AZIN1	antizyme inhibitor 1	0.010196	0.685913	-0.5439
211379_x_at	B3GALNT1	beta-1,3-N-acetylgalactosaminyltransferase 1 (globoside blood group)	0.029656	0.600931	-0.73473
217452_s_at	B3GALT2	UDP-Gal:betaGlcNAc beta 1,3-galactosyltransferase, polypeptide 2	0.005839	0.449263	-1.15437
221240_s_at	B3GNT4	UDP-GlcNAc:betaGal beta-1,3-N-acetylglucosaminyltransferase 4	0.014265	1.5765	0.656729
206233_at	B4GALT6	UDP-Gal:betaGlcNAc beta 1,4-galactosyltransferase, polypeptide 6	0.0035	0.425005	-1.23445
217911_s_at	BAG3	BCL2-associated athanogene 3	0.000224	1.96131	0.971819
202984_s_at	BAG5	BCL2-associated athanogene 5	0.019365	0.39076	-1.35564
205638_at	BAI3	brain-specific angiogenesis inhibitor 3	0.020068	0.721969	-0.46999
203304_at	BAMBI	BMP and activin membrane-bound inhibitor homolog (Xenopus laevis)	0.021346	1.50188	0.586772
203053_at	BCAS2	breast carcinoma amplified sequence 2	0.001611	0.648376	-0.6251
203576_at	BCAT2	branched chain amino-acid transaminase 2, mitochondrial	0.016533	1.53574	0.618933
219497_s_at	BCL11A	B-cell CLL/lymphoma 11A (zinc finger protein)	0.004535	0.65044	-0.62051
221478_at	BCL2/adenovirus E1B 19kDa interacting protein 3-like	chr8:26240544-26270640 (+) // 96.58 // p21.2	0.01978	0.70765	-0.49889
207618_s_at	BCS1L	BCS1-like (S. cerevisiae)	0.020906	1.54017	0.623092

219670_at	BEND5	BEN domain containing 5	0.027639	0.690255	-0.5348
202710_at	BET1	blocked early in transport 1 homolog (S. cerevisiae)	0.039031	0.690443	-0.53441
201261_x_at	BGN	biglycan	0.03844	1.30045	0.379011
221530_s_at	BHLHE41	basic helix-loop-helix family, member e41	0.046878	0.653035	-0.61477
202076_at	BIRC2	baculoviral IAP repeat-containing 2	0.023579	0.710384	-0.49333
202265_at	BMI1	BMI1 polycomb ring finger oncogene	0.042332	0.722961	-0.46801
37226_at	BNIP1	BCL2/adenovirus E1B 19kDa interacting protein 1	0.034932	1.36041	0.444043
201849_at	BNIP3	BCL2/adenovirus E1B 19kDa interacting protein 3	0.003967	0.718148	-0.47765
218955_at	BRF2	BRF2, subunit of RNA polymerase III transcription initiation factor, BRF1-like	0.038988	1.31849	0.398888
217945_at	BTBD1	BTB (POZ) domain containing 1	0.011745	0.714617	-0.48476
207722_s_at	BTBD2	BTB (POZ) domain containing 2	0.024006	2.22391	1.1531
202946_s_at	BTBD3	BTB (POZ) domain containing 3	0.012325	0.718532	-0.47688
201458_s_at	BUB3	budding uninhibited by benzimidazoles 3 homolog (yeast)	0.046898	0.680367	-0.55562
205839_s_at	BZRAP1	benzodiazapine receptor (peripheral) associated protein 1	0.012943	1.49714	0.582208
55662_at	C10orf76	chromosome 10 open reading frame 76	0.003105	1.62329	0.698917
201784_s_at	C11orf58	chromosome 11 open reading frame 58	0.01721	0.763538	-0.38923
204521_at	C12orf24	chromosome 12 open reading frame 24	0.013242	0.738481	-0.43737
218183_at	C16orf5	chromosome 16 open reading frame 5	0.047602	1.55814	0.639828
218945_at	C16orf68	chromosome 16 open reading frame 68	0.006544	0.738186	-0.43794
212055_at	C18orf10	chromosome 18 open reading frame 10	0.000447	0.67719	-0.56237
200076_s_at	C19orf50	chromosome 19 open reading frame 50	0.021617	1.334	0.415763
219439_at	C1GALT1	core 1 synthase, glycoprotein-N-acetylgalactosamine 3-beta-galactosyltransferase, 1	0.040741	0.633851	-0.65779
205103_at	C1orf61	chromosome 1 open reading frame 61	0.038595	1.33606	0.417988
209006_s_at	C1orf63	chromosome 1 open reading frame 63	0.010464	0.481159	-1.05541
219617_at	C2orf34	chromosome 2 open reading frame 34	0.002373	1.56395	0.645193
219176_at	C2orf47	chromosome 2 open reading frame 47	0.012286	0.735376	-0.44345
219288_at	C3orf14	chromosome 3 open reading frame 14	0.0322	0.729973	-0.45409
218646_at	C4orf27	chromosome 4 open reading frame 27	0.010445	0.723082	-0.46777
203738_at	C5orf22	chromosome 5 open reading frame 22	0.024369	0.676662	-0.56349
221823_at	C5orf30	chromosome 5 open reading frame 30	0.006286	0.635753	-0.65346
218674_at	C5orf44	chromosome 5 open reading frame 44	0.048096	0.689925	-0.53549
47083_at	C7orf26	chromosome 7 open reading frame 26	0.036286	1.68217	0.750326
220889_s_at	CA10	carbonic anhydrase X	0.029423	0.520755	-0.94132
211761_s_at	CACYBP	calcyclin binding protein	0.002868	0.650303	-0.62082
219572_at	CADPS2	Ca++-dependent secretion activator 2	0.015979	0.669094	-0.57972
34846_at	CAMK2B	calcium/calmodulin-dependent protein kinase II beta	0.005519	1.30496	0.384002
208838_at	CAND1	cullin-associated and neddylation-dissociated 1	0.032383	0.619558	-0.69069
212551_at	CAP2	CAP, adenylate cyclase-associated protein, 2 (yeast)	0.049183	0.526811	-0.92464
208374_s_at	CAPZA1	capping protein (actin filament) muscle Z-line, alpha 1	0.00488	0.666399	-0.58554
201238_s_at	CAPZA2	capping protein (actin filament) muscle Z-line, alpha 2	0.016874	0.681928	-0.55231
218153_at	CARS2	cysteinyl-tRNA synthetase 2, mitochondrial (putative)	0.033366	1.53161	0.615048
219342_at	CASD1	CAS1 domain containing 1	0.025255	0.696183	-0.52246
211366_x_at	CASP1	caspase 1, apoptosis-related cysteine peptidase (interleukin 1, beta, convertase)	0.008157	1.83336	0.874491
201091_s_at	CBX3	chromobox homolog 3	0.036304	0.576735	-0.79402
202048_s_at	CBX6	chromobox homolog 6	0.004933	1.48525	0.570708
218936_s_at	CCDC59	coiled-coil domain containing 59	0.006702	0.724221	-0.4655
218545_at	CCDC91	coiled-coil domain containing 91	0.019297	0.71039	-0.49332
202769_at	CCNG2	cyclin G2	0.020717	0.759887	-0.39614
222156_x_at	CCPG1	cell cycle progression 1	0.020332	0.588776	-0.76421
207445_s_at	CCR9	chemokine (C-C motif) receptor 9	0.032493	0.658466	-0.60282
209583_s_at	CD200	CD200 molecule	0.00376	0.61193	-0.70856
217880_at	CDC27	cell division cycle 27 homolog (S. cerevisiae)	0.005943	0.704171	-0.506
215296_at	CDC42BPA	CDC42 binding protein kinase alpha	0.034048	2.19609	1.13494

		(DMPK-like)			
207149_at	CDH12	cadherin 12, type 2 (N-cadherin 2)	0.000233	0.396743	-1.33372
212401_s_at	CDK11A	cyclin-dependent kinase 11A	0.007044	1.4752	0.560908
211502_s_at	CDK14	cyclin-dependent kinase 14	0.044313	0.701983	-0.51049
208824_x_at	CDK16	cyclin-dependent kinase 16	0.025817	1.36381	0.447645
221918_at	CDK17	cyclin-dependent kinase 17	0.00276	0.755231	-0.40501
204831_at	CDK8	cyclin-dependent kinase 8	0.004716	0.582181	-0.78046
213183_s_at	CDKN1C	Cyclin-dependent kinase inhibitor 1C (p57, Kip2)	0.003683	0.356517	-1.48796
202158_s_at	CELF2	CUGBP, Elav-like family member 2	0.012974	0.585861	-0.77137
212437_at	CENPB	centromere protein B, 80kDa	0.012814	1.61434	0.690947
207719_x_at	CEP170	centrosomal protein 170kDa	0.000542	0.694409	-0.52614
204373_s_at	CEP350	centrosomal protein 350kDa	0.02555	0.686832	-0.54197
212677_s_at	CEP68	centrosomal protein 68kDa	0.008751	0.646254	-0.62983
204591_at	CHL1	cell adhesion molecule with homology to L1CAM (close homolog of L1)	0.000991	0.515973	-0.95463
218085_at	CHMP5	chromatin modifying protein 5	0.002367	0.626465	-0.67469
213385_at	CHN2	chimerin (chimaerin) 2	0.022086	0.72908	-0.45585
218566_s_at	CHORDC1	cysteine and histidine-rich domain (CHORD)-containing 1	0.017649	0.638922	-0.64629
218252_at	CKAP2	cytoskeleton associated protein 2	0.035323	0.716929	-0.4801
205295_at	CKMT2	creatine kinase, mitochondrial 2 (sarcomeric)	0.016434	1.51794	0.602113
207855_s_at	CLCC1	chloride channel CLIC-like 1	0.015247	0.520046	-0.94329
214769_at	CLCN4	chloride channel 4	0.008549	0.595571	-0.74766
207995_s_at	CLEC4M	C-type lectin domain family 4, member M	0.047414	1.31406	0.394033
210346_s_at	CLK4	CDC-like kinase 4	0.010027	0.725144	-0.46366
216295_s_at	CLTA	clathrin, light chain A	0.018852	0.76402	-0.38832
202164_s_at	CNOT8	CCR4-NOT transcription complex, subunit 8	0.02519	0.7055	-0.50328
209797_at	CNPY2	canopy 2 homolog (zebrafish)	0.001785	0.675694	-0.56556
201913_s_at	COASY	CoA synthase	0.007531	1.35375	0.436962
211964_at	COL4A2	collagen, type IV, alpha 2	0.044268	1.67104	0.740749
202467_s_at	COPS2	COP9 constitutive photomorphogenic homolog subunit 2 (Arabidopsis)	0.035105	0.73176	-0.45056
218042_at	COPS4	COP9 constitutive photomorphogenic homolog subunit 4 (Arabidopsis)	0.005212	0.63955	-0.64487
201652_at	COPS5	COP9 constitutive photomorphogenic homolog subunit 5 (Arabidopsis)	0.012363	0.723034	-0.46786
214260_at	COPS8	COP9 constitutive photomorphogenic homolog subunit 8 (Arabidopsis)	0.014202	0.591962	-0.75642
219397_at	COQ10B	coenzyme Q10 homolog B (S. cerevisiae)	0.024261	0.767977	-0.38087
211727_s_at	COX11	COX11 cytochrome c oxidase assembly homolog (yeast)	0.015065	0.70779	-0.49861
203663_s_at	COX5A	cytochrome c oxidase subunit Va	0.019499	0.703938	-0.50648
202110_at	COX7B	cytochrome c oxidase subunit VIIb	0.013354	0.725184	-0.46358
206918_s_at	CPNE1	copine I	0.019472	1.46939	0.555216
218142_s_at	CRBN	cereblon	0.01415	0.619491	-0.69085
204313_s_at	CREB1	cAMP responsive element binding protein 1	0.028538	0.697389	-0.51997
201112_s_at	CSE1L	CSE1 chromosome segregation 1-like (yeast)	0.00698	0.672232	-0.57297
200621_at	CSRP1	cysteine and glycine-rich protein 1	0.03168	1.50335	0.588178
201360_at	CST3	cystatin C	0.041468	1.46864	0.554486
208407_s_at	CTNND1	catenin (cadherin-associated protein), delta 1	0.016104	1.42245	0.508381
209617_s_at	CTNND2	catenin (cadherin-associated protein), delta 2 (neural plakophilin-related arm-repeat protein)	0.001324	0.688477	-0.53852
202087_s_at	CTSL1	cathepsin L1	0.034843	0.482734	-1.0507
201059_at	CTTN	cortactin	0.012748	1.35973	0.443317
207614_s_at	CUL1	cullin 1	0.039014	0.767814	-0.38117
201424_s_at	CUL4A	cullin 4A	0.009722	0.497192	-1.00812
203533_s_at	CUL5	cullin 5	0.000291	0.359368	-1.47647
208839_s_at	cullin-associated and neddylation-dissociated 1	chr12:67663148-67707920 (+) // 97.2 // q14.3	0.037974	0.738746	-0.43685
201634_s_at	CYB5B	cytochrome b5 type B (outer mitochondrial membrane)	0.014957	1.45467	0.540696
201885_s_at	CYB5R3	cytochrome b5 reductase 3	0.009514	1.44156	0.52763

213072_at	CYHR1	cysteine/histidine-rich 1	0.020005	1.84867	0.886484
216288_at	CYSLTR1	cysteinyl leukotriene receptor 1	0.004825	1.39352	0.47873
212309_at	cytoplasmic linker associated protein 2	chr3:33537740-33700598 (-) // 98.55 // p22.3	0.019582	0.763521	-0.38926
203302_at	DCK	deoxycytidine kinase	0.027088	0.733113	-0.44789
215303_at	DCLK1	doublecortin-like kinase 1	0.042774	0.384131	-1.38033
211896_s_at	DCN	decorin	0.002773	1.39732	0.482658
217973_at	DCXR	dicarbonyl/L-xylulose reductase	0.042027	1.76328	0.818264
214909_s_at	DDAH2	dimethylarginine dimethylaminohydrolase 2	0.001229	1.84402	0.882854
208675_s_at	DDOST	dolichyl-diphosphooligosaccharide--protein glycosyltransferase	0.027111	0.703585	-0.5072
210749_x_at	DDR1	discoidin domain receptor tyrosine kinase 1	0.014806	1.37987	0.464533
201241_at	DDX1	DEAD (Asp-Glu-Ala-Asp) box polypeptide 1	0.030821	0.71734	-0.47927
205000_at	DDX3Y	DEAD (Asp-Glu-Ala-Asp) box polypeptide 3, Y-linked	0.024183	0.668744	-0.58047
202447_at	DECR1	2,4-dienoyl CoA reductase 1, mitochondrial	0.048442	1.35958	0.443159
219646_at	DEF8	differentially expressed in FDCP 8 homolog (mouse)	0.038537	1.44349	0.529558
209250_at	degenerative spermatocyte homolog 1, lipid desaturase (Drosophila)	chr1:224370927-224381140 (+) // 81.2 // q42.11	0.003131	0.657594	-0.60473
53991_at	DENND2A	DENN/MADD domain containing 2A	0.014923	1.63654	0.710653
219402_s_at	DERL1	Der1-like domain family, member 1	0.020358	0.65993	-0.59962
206061_s_at	DICER1	dicer 1, ribonuclease type III	0.02376	0.407205	-1.29617
219619_at	DIRAS2	DIRAS family, GTP-binding RAS-like 2	0.035174	0.554854	-0.84982
214724_at	DIXDC1	DIX domain containing 1	0.022242	0.518956	-0.94632
205677_s_at	DLEU1	deleted in lymphocytic leukemia 1 (non-protein coding)	1.25E-05	1.41718	0.503023
202514_at	DLG1	discs, large homolog 1 (Drosophila)	0.008849	0.549218	-0.86455
203881_s_at	DMD	dystrophin	0.047248	0.666308	-0.58574
203791_at	DMXL1	Dmx-like 1	0.047234	0.751829	-0.41152
212820_at	DMXL2	Dmx-like 2	0.000993	0.565687	-0.82192
200881_s_at	DNAJA1	DnaJ (Hsp40) homolog, subfamily A, member 1	0.041364	0.739452	-0.43547
219237_s_at	DNAJB14	DnaJ (Hsp40) homolog, subfamily B, member 14	0.002997	0.430479	-1.21598
208810_at	DNAJB6 /// TMEM135	DnaJ (Hsp40) homolog, subfamily B, member 6 /// transmembrane protein 135	0.005088	0.663916	-0.59093
204720_s_at	DNAJC6	DnaJ (Hsp40) homolog, subfamily C, member 6	0.00198	0.720185	-0.47356
203187_at	DOCK1	dedicator of cytokinesis 1	0.006787	1.8743	0.906348
212538_at	DOCK9	dedicator of cytokinesis 9	0.004684	0.65153	-0.6181
208141_s_at	DOHH	deoxyhypusine hydroxylase/monooxygenase	0.039911	0.43884	-1.18823
219746_at	DPF3	D4, zinc and double PHD fingers, family 3	0.00418	1.39901	0.484404
222041_at	DPH1 /// OVCA2	DPH1 homolog (S. cerevisiae) /// ovarian tumor suppressor candidate 2	0.009201	2.14447	1.10062
202673_at	DPM1	dolichyl-phosphate mannosyltransferase polypeptide 1, catalytic subunit	0.000733	0.549793	-0.86304
215143_at	dpy-19-like 2 pseudogene 2 (C. elegans)	chr12:64053558-64055463 (-) // 61.53 // q14.2 /// chr7:102910392-102912520 (-) // 64.43 // q22.1 /// chr7:35217078-35218968 (-) // 63.03 // p14.2 /// chr7:32791700-32795230 (-) // 57.8 // p14.3	0.014558	0.575903	-0.7961
219065_s_at	DPY30 /// MEMO1	dpy-30 homolog (C. elegans) /// mediator of cell motility 1	0.001044	0.45068	-1.14982
209187_at	DR1	down-regulator of transcription 1, TBP-binding (negative cofactor 2)	0.009256	0.667327	-0.58353
208486_at	DRD5	dopamine receptor D5	0.013356	1.36463	0.448512
201021_s_at	DSTN	destrin (actin depolymerizing factor)	0.031601	0.690812	-0.53363
218845_at	DUSP22	dual specificity phosphatase 22	0.008421	0.68126	-0.55372
208891_at	DUSP6	dual specificity phosphatase 6	0.035977	0.617489	-0.69552
208956_x_at	DUT	deoxyuridine triphosphatase	0.007425	0.677334	-0.56206
205348_s_at	DYNC1I1	dynein, cytoplasmic 1, intermediate chain 1	0.043225	0.698771	-0.51711

217976_s_at	DYNC1LI1	dynein, cytoplasmic 1, light intermediate chain 1	0.021128	0.623293	-0.68202
203303_at	DYNLT3	dynein, light chain, Tctex-type 3	0.017696	0.752686	-0.40988
209033_s_at	DYRK1A	dual-specificity tyrosine-(Y)-phosphorylation regulated kinase 1A	0.015348	0.729911	-0.45421
213186_at	DZIP3	DAZ interacting protein 3, zinc finger	0.019874	0.7009	-0.51272
202623_at	EAPP	E2F-associated phosphoprotein	0.020003	0.531472	-0.91194
218552_at	ECHDC2	enoyl CoA hydratase domain containing 2	0.000358	1.59756	0.67587
219787_s_at	ECT2	epithelial cell transforming sequence 2 oncogene	0.036012	0.645966	-0.63047
204271_s_at	EDNRB	endothelin receptor type B	0.006594	1.96032	0.971093
204905_s_at	EEF1E1	eukaryotic translation elongation factor 1 epsilon 1	0.008006	0.512175	-0.96529
212410_at	EFHA1	EF-hand domain family, member A1	0.039038	0.757645	-0.40041
212149_at	EFR3A	EFR3 homolog A (S. cerevisiae)	0.027434	0.729312	-0.45539
219454_at	EGFL6	EGF-like-domain, multiple 6	0.003705	1.33592	0.417829
211551_at	EGFR	epidermal growth factor receptor	0.008477	1.5021	0.586983
201694_s_at	EGR1	early growth response 1	0.015872	0.560323	-0.83567
212653_s_at	EHBP1	EH domain binding protein 1	0.023133	0.745347	-0.42402
211698_at	EID1	EP300 interacting inhibitor of differentiation 1	0.024035	0.577569	-0.79193
201019_s_at	EIF1AX	eukaryotic translation initiation factor 1A, X-linked	0.007609	0.515312	-0.95648
201738_at	EIF1B	eukaryotic translation initiation factor 1B	0.002729	0.720665	-0.4726
202232_s_at	EIF3M	eukaryotic translation initiation factor 3, subunit M	0.047116	0.625782	-0.67627
201436_at	EIF4E	eukaryotic translation initiation factor 4E	0.039354	0.711246	-0.49158
208290_s_at	EIF5	eukaryotic translation initiation factor 5	0.025595	0.69134	-0.53253
204256_at	ELOVL6	ELOVL family member 6, elongation of long chain fatty acids (FEN1/Elo2, SUR4/Elo3-like, yeast)	0.034	0.717964	-0.47802
219134_at	ELTD1	EGF, latrophilin and seven transmembrane domain containing 1	0.043331	1.56951	0.650312
213779_at	EMID1	EMI domain containing 1	0.003087	1.3488	0.431673
204796_at	EML1	echinoderm microtubule associated protein like 1	0.024111	0.642679	-0.63783
65635_at	ENGASE	endo-beta-N-acetylglucosaminidase	0.005817	1.35983	0.443431
201231_s_at	ENO1	enolase 1, (alpha)	0.000888	1.38828	0.473301
217956_s_at	ENOPH1	enolase-phosphatase 1	0.006542	0.689712	-0.53593
204161_s_at	ENPP4	ectonucleotide pyrophosphatase/phosphodiesterase 4 (putative)	0.010452	0.54563	-0.87401
221487_s_at	ENSA	endosulfine alpha	0.002299	0.36401	-1.45795
204077_x_at	ENTPD4	ectonucleoside triphosphate diphosphohydrolase 4	0.023183	0.714127	-0.48575
200878_at	EPAS1	endothelial PAS domain protein 1	0.022228	1.59683	0.675209
206710_s_at	EPB41L3	erythrocyte membrane protein band 4.1-like 3	0.003908	0.535565	-0.90087
220977_x_at	EPB41L5	erythrocyte membrane protein band 4.1 like 5	0.003545	1.70241	0.767576
215664_s_at	EPHA5	EPH receptor A5	0.010957	0.410606	-1.28418
200842_s_at	EPRS	glutamyl-prolyl-tRNA synthetase	0.01464	0.667164	-0.58389
217887_s_at	EPS15	epidermal growth factor receptor pathway substrate 15	0.00531	0.710675	-0.49274
213541_s_at	ERG	v-ets erythroblastosis virus E26 oncogene homolog (avian)	0.02576	1.47099	0.556784
218135_at	ERGIC2	ERGIC and golgi 2	0.049058	0.580776	-0.78395
218859_s_at	ESF1	ESF1, nucleolar pre-rRNA processing protein, homolog (S. cerevisiae)	0.043084	0.597337	-0.74338
205530_at	ETFDH	electron-transferring-flavoprotein dehydrogenase	0.006948	1.45735	0.543349
210011_s_at	EWSR1	Ewing sarcoma breakpoint region 1	0.014044	1.34249	0.424911
213648_at	EXOSC7	exosome component 7	0.025182	1.3945	0.479751
208623_s_at	EZR	ezrin	0.035072	2.03099	1.02219
204363_at	F3	coagulation factor III (thromboplastin, tissue factor)	0.017222	1.77229	0.825618
204257_at	FADS3	fatty acid desaturase 3	0.015744	1.81455	0.859609
207547_s_at	FAM107A	family with sequence similarity 107, member A	0.011572	1.82577	0.868505

202972_s_at	FAM13A	family with sequence similarity 13, member A	0.021615	0.707394	-0.49941
213304_at	FAM179B	family with sequence similarity 179, member B	0.003899	0.64271	-0.63776
218297_at	FAM188A	family with sequence similarity 188, member A	0.034183	0.744603	-0.42546
209379_s_at	FAM190B	family with sequence similarity 190, member B	0.007479	0.677368	-0.56199
202915_s_at	FAM20B	family with sequence similarity 20, member B	0.032981	0.736634	-0.44098
211068_x_at	FAM21C /// FAM21D	family with sequence similarity 21, member C /// family with sequence similarity 21, member D	0.035327	0.757115	-0.40142
201889_at	FAM3C	family with sequence similarity 3, member C	0.010517	0.57175	-0.80654
209074_s_at	family with sequence similarity 107, member A	chr3:58551187-58563104 (-) // 85.42 // p14.3	0.033511	1.43155	0.51758
218518_at	family with sequence similarity 13, member B	chr5:137273650-137368720 (-) // 99.82 // q31.2	0.01098	0.708455	-0.49725
212370_x_at	family with sequence similarity 21, member A /// family with sequence similarity 21, member B /// family with sequence similarity 21, member C	chr10:51827666-51893268 (+) // 95.96 // q11.23 /// chr10:46222666-46288411 (+) // 94.53 // q11.22 /// chr10:47894571-47949416 (+) // 86.86 // q11.22	0.000836	0.696315	-0.52219
212333_at	family with sequence similarity 98, member A	chr2:33808728-33824354 (-) // 96.6 // p22.3	0.002474	0.648585	-0.62463
215000_s_at	fasciculation and elongation protein zeta 2 (zygin II)	chr2:36779406-36786398 (-) // 84.77 // p22.2	0.003779	0.757703	-0.4003
202271_at	FBXO28	F-box protein 28	0.015132	0.661504	-0.59618
218432_at	FBXO3	F-box protein 3	0.013365	0.679742	-0.55694
212987_at	FBXO9	F-box protein 9	0.000435	0.641958	-0.63945
209630_s_at	FBXW2	F-box and WD repeat domain containing 2	0.007748	0.604031	-0.72731
218751_s_at	FBXW7	F-box and WD repeat domain containing 7	0.044846	0.669721	-0.57837
208647_at	FDFT1	farnesyl-diphosphate farnesyltransferase 1	0.00873	0.756739	-0.40213
212373_at	FEM1B	fem-1 homolog b (C. elegans)	0.012838	0.616248	-0.69842
202305_s_at	FEZ2	fasciculation and elongation protein zeta 2 (zygin II)	0.011769	0.565525	-0.82234
221086_s_at	FEZF2	FEZ family zinc finger 2	0.042416	1.42466	0.51062
214589_at	FGF12	fibroblast growth factor 12	0.00261	0.604416	-0.72639
205110_s_at	FGF13	fibroblast growth factor 13	0.012239	0.59887	-0.73969
206404_at	FGF9	fibroblast growth factor 9 (glia-activating factor)	0.030091	0.680035	-0.55632
204379_s_at	FGFR3	fibroblast growth factor receptor 3	0.028611	1.43131	0.517339
209931_s_at	FKBP1B /// MFSD2B	FK506 binding protein 1B, 12.6 kDa /// major facilitator superfamily domain containing 2B	0.009928	1.6871	0.754548
218003_s_at	FKBP3	FK506 binding protein 3, 25kDa	0.022607	0.693984	-0.52703
219250_s_at	FLRT3	fibronectin leucine rich transmembrane protein 3	0.013283	0.592375	-0.75542
205022_s_at	FOXN3	forkhead box N3	0.020439	0.509269	-0.9735
204072_s_at	FRY	furry homolog (Drosophila)	0.018969	0.646395	-0.62951
203592_s_at	FSTL3	follistatin-like 3 (secreted glycoprotein)	0.005486	1.42454	0.510497
205384_at	FXYD1	FXYD domain containing ion transport regulator 1	0.015908	1.46176	0.547707
208841_s_at	G3BP2	GTPase activating protein (SH3 domain) binding protein 2	0.014296	0.692392	-0.53034
208868_s_at	GABA(A) receptor-associated protein like 1	chr12:10365448-10375731 (+) // 98.25 // p13.2	0.029726	0.614319	-0.70294
211458_s_at	GABARAPL1 /// GABARAPL3	GABA(A) receptor-associated protein like 1 /// GABA(A) receptors associated protein like 3, pseudogene	0.037611	0.736428	-0.44138

206678_at	GABRA1	gamma-aminobutyric acid (GABA) A receptor, alpha 1	0.041348	0.472858	-1.08052
207014_at	GABRA2	gamma-aminobutyric acid (GABA) A receptor, alpha 2	0.017166	0.561118	-0.83362
206525_at	GABRR1	gamma-aminobutyric acid (GABA) receptor, rho 1	0.014592	1.4947	0.579858
219013_at	GALNT11	UDP-N-acetyl-alpha-D-galactosamine:polypeptide N-acetylgalactosaminyltransferase 11 (GalNAc-T11)	0.008559	0.716166	-0.48163
204471_at	GAP43	growth associated protein 43	0.044624	0.707316	-0.49957
209710_at	GATA2	GATA binding protein 2	0.044922	0.698461	-0.51775
203282_at	GBE1	glucan (1,4-alpha-), branching enzyme 1	0.016533	0.580229	-0.78531
203765_at	GCA	grancalcin, EF-hand calcium binding protein	0.024146	0.649264	-0.62312
36475_at	GCAT	glycine C-acetyltransferase	0.011861	1.39908	0.484482
218912_at	GCC1	GRIP and coiled-coil domain containing 1	0.005685	1.41723	0.503079
212241_at	GCOM1 /// GRINL1A	GRINL1A complex locus /// glutamate receptor, ionotropic, N-methyl D-aspartate-like 1A	0.023239	0.646401	-0.6295
221314_at	GDF9	growth differentiation factor 9	0.003083	1.41963	0.505518
202722_s_at	GFPT1	glutamine--fructose-6-phosphate transaminase 1	0.025071	0.71508	-0.48382
207131_x_at	GGT1	gamma-glutamyltransferase 1	0.02549	1.48862	0.573979
211416_x_at	GGTLC1	gamma-glutamyltransferase light chain 1	0.018699	1.64196	0.715418
209249_s_at	GHITM	growth hormone inducible transmembrane protein	0.026111	0.766686	-0.38329
214730_s_at	GLG1	golgi glycoprotein 1	0.029361	0.70796	-0.49826
207153_s_at	GLMN	glomulin, FKBP associated protein	0.012837	0.534418	-0.90396
205279_s_at	GLRB	glycine receptor, beta	0.024226	0.579108	-0.7881
206662_at	GLRX	glutaredoxin (thioltransferase)	0.01153	0.618631	-0.69285
219933_at	GLRX2	glutaredoxin 2	0.01839	0.632736	-0.66032
203158_s_at	GLS	glutaminase	0.008612	0.598641	-0.74024
212414_s_at	glyoxylate reductase 1 homolog (Arabidopsis) /// septin 6	chrX:118750910-118827323 (-) // 91.58 // q24	0.022189	1.53245	0.615841
218506_x_at	GLYR1	glyoxylate reductase 1 homolog (Arabidopsis)	0.034226	0.732636	-0.44883
200744_s_at	GNB1	guanine nucleotide binding protein (G protein), beta polypeptide 1	0.01664	0.571749	-0.80655
200651_at	GNB2L1	guanine nucleotide binding protein (G protein), beta polypeptide 2-like 1	0.00836	1.30899	0.388456
207124_s_at	GNB5	guanine nucleotide binding protein (G protein), beta 5	0.014296	0.435807	-1.19824
212959_s_at	GNPTAB	N-acetylglucosamine-1-phosphate transferase, alpha and beta subunits	0.044863	0.724816	-0.46431
215203_at	GOLGA4	golgin A4	0.010164	0.436295	-1.19662
211014_s_at	GOLGA6L4 /// PML	golgin A6 family-like 4 /// promyelocytic leukemia	0.027568	1.33987	0.422088
208843_s_at	GORASP2	golgi reassembly stacking protein 2, 55kDa	0.001704	0.707591	-0.49901
213180_s_at	GOSR2	golgi SNAP receptor complex member 2	0.018306	1.37278	0.457096
207174_at	GPC5	glypican 5	0.044857	2.56034	1.35633
201141_at	GPNMB	glycoprotein (transmembrane) nmb	0.030526	1.60047	0.678496
221306_at	GPR27	G protein-coupled receptor 27	0.031625	2.12783	1.08938
212856_at	GRAMD4	GRAM domain containing 4	0.000156	1.37207	0.456357
205358_at	GRIA2	glutamate receptor, ionotropic, AMPA 2	0.024584	0.612367	-0.70753
206730_at	GRIA3	glutamate receptor, ionotrophic, AMPA 3	0.029177	0.60384	-0.72776
207548_at	GRM7	glutamate receptor, metabotropic 7	0.001476	0.599093	-0.73915
201520_s_at	GRSF1	G-rich RNA sequence binding factor 1	0.020664	0.73121	-0.45164
215333_x_at	GSTM1	glutathione S-transferase mu 1	0.007879	1.32567	0.406719
205752_s_at	GSTM5	glutathione S-transferase mu 5	0.021667	1.3609	0.444561
202680_at	GTF2E2	general transcription factor IIE, polypeptide 2, beta 34kDa	0.046991	1.96108	0.97165
209595_at	GTF2F2	general transcription factor IIF, polypeptide 2, 30kDa	0.017529	2.34017	1.22661
202451_at	GTF2H1	general transcription factor IIH, polypeptide 1, 62kDa	0.048207	0.710556	-0.49298
213357_at	GTF2H5	general transcription factor IIH,	0.036808	0.739798	-0.4348

		polypeptide 5			
201065_s_at	GTF2I /// GTF2IP1 /// LOC100093631	general transcription factor IIi /// general transcription factor IIi, pseudogene 1 /// general transcription factor II, i, pseudogene	0.044002	0.767632	-0.38151
218238_at	GTPBP4	GTP binding protein 4	0.032781	0.506008	-0.98277
209576_at	guanine nucleotide binding protein (G protein), alpha inhibiting activity polypeptide 1	chr7:79764161-79847350 (+) // 98.92 // q21.11	0.015792	0.528118	-0.92107
211555_s_at	GUCY1B3	guanylate cyclase 1, soluble, beta 3	0.041087	0.62927	-0.66825
204235_s_at	GULP1	GULP, engulfment adaptor PTB domain containing 1	0.010372	0.635583	-0.65385
202605_at	GUSB	glucuronidase, beta	0.00589	1.83566	0.876299
217232_x_at	HBB	hemoglobin, beta	0.024821	1.68712	0.754559
206106_at	HDAC10 /// LOC100509694 /// MAPK12	histone deacetylase 10 /// mitogen-activated protein kinase 12-like /// mitogen-activated protein kinase 12	0.029435	2.04873	1.03473
209524_at	HDGFRP3	hepatoma-derived growth factor, related protein 3	0.000119	0.639628	-0.6447
217902_s_at	HERC2	hect domain and RLD 2	0.032002	0.699872	-0.51484
204512_at	HIVEP1	human immunodeficiency virus type I enhancer binding protein 1	0.02469	0.695257	-0.52438
213932_x_at	HLA-A	major histocompatibility complex, class I, A	0.043527	1.30018	0.378717
214459_x_at	HLA-C	major histocompatibility complex, class I, C	0.042561	1.43731	0.523376
211529_x_at	HLA-G	major histocompatibility complex, class I, G	0.003996	1.7826	0.833986
219269_at	HMBOX1	homeobox containing 1	0.034264	1.32274	0.403526
202539_s_at	HMGCR	3-hydroxy-3-methylglutaryl-CoA reductase	0.02538	0.657001	-0.60603
205822_s_at	HMGCS1	3-hydroxy-3-methylglutaryl-CoA synthase 1 (soluble)	0.042734	0.668411	-0.58119
209786_at	HMGN4	high mobility group nucleosomal binding domain 4	0.040451	0.732772	-0.44856
216559_x_at	HNRNPA1 /// HNRNPA1L2 /// HNRNPA1P10 /// LOC728643	heterogeneous nuclear ribonucleoprotein A1 /// heterogeneous nuclear ribonucleoprotein A1-like 2 /// heterogeneous nuclear ribonucleoprotein A1 pseudogene 10 /// heterogeneous nuclear ribonucleoprotein A1 pseudogene	0.003185	1.34448	0.427045
211930_at	HNRNPA3	heterogeneous nuclear ribonucleoprotein A3	0.018538	0.705837	-0.50259
211933_s_at	HNRNPA3 /// HNRNPA3P1	heterogeneous nuclear ribonucleoprotein A3 /// heterogeneous nuclear ribonucleoprotein A3 pseudogene 1	0.012526	0.764415	-0.38757
200073_s_at	HNRNPD	heterogeneous nuclear ribonucleoprotein D (AU-rich element RNA binding protein 1, 37kDa)	0.011847	0.767381	-0.38199
213619_at	HNRNPH1	Heterogeneous nuclear ribonucleoprotein H1 (H)	0.007535	0.570365	-0.81004
207127_s_at	HNRNPH3	heterogeneous nuclear ribonucleoprotein H3 (2H9)	0.013953	0.625926	-0.67594
208766_s_at	HNRNPR	heterogeneous nuclear ribonucleoprotein R	0.003246	0.760312	-0.39534
202854_at	HPRT1	hypoxanthine phosphoribosyltransferase 1	0.032385	0.577602	-0.79185
203283_s_at	HS2ST1	heparan sulfate 2-O-sulfotransferase 1	0.042164	0.667309	-0.58357
209657_s_at	HSF2	heat shock transcription factor 2	0.01371	0.649462	-0.62268
210211_s_at	HSP90AA1	heat shock protein 90kDa alpha (cytosolic), class A member 1	0.004069	0.716112	-0.48174
200599_s_at	HSP90B1	heat shock protein 90kDa beta (Grp94), member 1	0.0065	0.726292	-0.46138
205543_at	HSPA4L	heat shock 70kDa protein 4-like	0.029511	0.675713	-0.56552
208687_x_at	HSPA8	heat shock 70kDa protein 8	0.007482	0.691902	-0.53136
205133_s_at	HSPE1	heat shock 10kDa protein 1 (chaperonin 10)	0.014219	0.685296	-0.5452
206976_s_at	HSPH1	heat shock 105kDa/110kDa protein 1	0.005477	0.639571	-0.64483
210253_at	HTATIP2	HIV-1 Tat interactive protein 2, 30kDa	0.030772	0.755182	-0.4051
212957_s_at	hypothetical LOC92249	chrX:62646437-62780873 (-) // 25.74 // q11.1	0.041144	2.05365	1.03819
217900_at	IARS2	isoleucyl-tRNA synthetase 2, mitochondrial	0.018697	0.703697	-0.50697
204002_s_at	ICA1	islet cell autoantigen 1, 69kDa	0.006976	1.33123	0.412764
204683_at	ICAM2	intercellular adhesion molecule 2	0.001362	1.76677	0.821118

208937_s_at	ID1	inhibitor of DNA binding 1, dominant negative helix-loop-helix protein	0.024974	1.40396	0.489506
201565_s_at	ID2	inhibitor of DNA binding 2, dominant negative helix-loop-helix protein	0.017104	0.756184	-0.40319
214059_at	IFI44	Interferon-induced protein 44	0.036472	0.620393	-0.68875
203628_at	IGF1R	insulin-like growth factor 1 receptor	0.027264	0.606363	-0.72175
202491_s_at	IKBKAP	inhibitor of kappa light polypeptide gene enhancer in B-cells, kinase complex-associated protein	0.00066	0.636578	-0.65159
205207_at	IL6	interleukin 6 (interferon, beta 2)	0.032321	1.67418	0.743453
203011_at	IMPA1	inositol(myo)-1(or 4)-monophosphatase 1	0.033671	0.745043	-0.4246
205981_s_at	ING2	inhibitor of growth family, member 2	0.000164	0.653315	-0.61415
222240_s_at	inositol-3-phosphate synthase 1	chr19:18545631-18548119 (-) // 98.77 // p13.11	0.015979	1.94998	0.963458
202782_s_at	INPP5K	inositol polyphosphate-5-phosphatase K	0.001056	1.50086	0.58579
218819_at	integrator complex subunit 6	chr13:51939363-52027134 (-) // 99.21 // q14.3 /// chr5:39718839-39721612 (-) // 72.68 // p13.1	0.027583	0.455281	-1.13517
221185_s_at	IQCG	IQ motif containing G	0.008535	0.729441	-0.45514
204030_s_at	IQCJ-SCHIP1 /// SCHIP1	IQ motif containing J-schwannomin interacting protein 1 read-through transcript /// schwannomin interacting protein 1	0.0013	0.617233	-0.69611
202621_at	IRF3	interferon regulatory factor 3	0.043977	0.447022	-1.16158
209274_s_at	ISCA1	iron-sulfur cluster assembly 1 homolog (S. cerevisiae)	0.011497	0.687214	-0.54117
206766_at	ITGA10	integrin, alpha 10	0.004949	1.38516	0.470048
37201_at	ITIH4	inter-alpha (globulin) inhibitor H4 (plasma Kallikrein-sensitive glycoprotein)	0.005643	1.41283	0.498591
217731_s_at	ITM2B	integral membrane protein 2B	0.02151	0.737245	-0.43978
221004_s_at	ITM2C	integral membrane protein 2C	0.000107	1.42875	0.51475
203723_at	ITPKB	inositol 1,4,5-trisphosphate 3-kinase B	0.009852	1.59729	0.67563
203710_at	ITPR1	inositol 1,4,5-triphosphate receptor, type 1	0.013242	0.503682	-0.98941
206245_s_at	IVNS1ABP	influenza virus NS1A binding protein	0.00767	0.61189	-0.70866
205889_s_at	JAKMIP2	janus kinase and microtubule interacting protein 2	0.037907	0.758526	-0.39873
218418_s_at	KANK2	KN motif and ankyrin repeat domains 2	0.024385	1.33178	0.413354
206689_x_at	KAT5	K(lysine) acetyltransferase 5	0.031241	1.39469	0.479939
210078_s_at	KCNAB1	potassium voltage-gated channel, shaker-related subfamily, beta member 1	0.018751	0.534631	-0.90339
210263_at	KCNF1	potassium voltage-gated channel, subfamily F, member 1	0.029057	0.361523	-1.46784
204679_at	KCNK1	potassium channel, subfamily K, member 1	0.000773	0.498555	-1.00418
211713_x_at	KIAA0101	KIAA0101	0.023072	1.92737	0.946632
212523_s_at	KIAA0146	KIAA0146	0.040999	0.571845	-0.8063
212428_at	KIAA0368	KIAA0368	0.020319	0.757444	-0.40079
203955_at	KIAA0649	KIAA0649	0.030746	0.461596	-1.1153
212779_at	KIAA1109	KIAA1109	0.004323	0.676021	-0.56486
203087_s_at	KIF2A	kinesin heavy chain member 2A	0.002304	0.597841	-0.74217
213623_at	KIF3A	kinesin family member 3A	0.011626	0.373056	-1.42254
203390_s_at	KIF3C	kinesin family member 3C	0.041687	0.670712	-0.57624
213656_s_at	kinesin light chain 1	chr14:104166258-104167880 (+) // 97.62 // q32.33	0.034732	1.50507	0.589826
205978_at	KL	klotho	0.027741	1.67215	0.741707
202393_s_at	KLF10	Kruppel-like factor 10	0.032689	0.647644	-0.62673
203543_s_at	KLF9	Kruppel-like factor 9	0.040094	0.580918	-0.78359
209256_s_at	KLHDC10	kelch domain containing 10	0.034507	1.36282	0.446596
217906_at	KLHDC2	kelch domain containing 2	0.030708	0.768193	-0.38046
221219_s_at	KLHDC4	kelch domain containing 4	0.019847	1.69643	0.7625
219157_at	KLHL2	kelch-like 2, Mayven (Drosophila)	0.042757	0.747942	-0.419
201088_at	KPNA2	karyopherin alpha 2 (RAG cohort 1, importin alpha 1)	0.025594	0.674401	-0.56832
204009_s_at	KRAS	v-Ki-ras2 Kirsten rat sarcoma viral oncogene homolog	0.008365	0.730423	-0.4532
34031_i_at	KRIT1	KRIT1, ankyrin repeat containing	0.006014	0.588233	-0.76554
215189_at	KRT86 /// LOC100509764	keratin 86 /// hypothetical LOC100509764	0.010793	1.30074	0.379331
200914_x_at	KTN1	kinectin 1 (kinesin receptor)	0.03553	0.594256	-0.75084

202020_s_at	LANCL1	LanC lantibiotic synthetase component C-like 1 (bacterial)	0.00795	0.698644	-0.51737
208029_s_at	LAPTM4B	lysosomal protein transmembrane 4 beta	0.037692	0.592106	-0.75607
208953_at	LARP4B	La ribonucleoprotein domain family, member 4B	0.000455	0.692406	-0.53031
206268_at	LEFTY1	left-right determination factor 1	0.024067	1.67016	0.73999
208949_s_at	LGALS3	lectin, galactoside-binding, soluble, 3	0.033236	1.64084	0.714438
218253_s_at	LGTN	ligatin	0.036263	1.90053	0.926403
200805_at	LMAN2	lectin, mannose-binding 2	0.031497	2.02352	1.01686
221847_at	LOC100129361	hypothetical LOC100129361	0.048571	0.745759	-0.42322
215287_at	LOC100288939	Similar to hCG1987955	0.036023	0.564227	-0.82565
216336_x_at	LOC100505584 /// MT1E	hypothetical protein LOC100505584 /// metallothionein 1E	0.004703	2.1338	1.09342
213971_s_at	LOC100510525 /// SUZ12 /// SUZ12P	polycomb protein SUZ12-like /// suppressor of zeste 12 homolog (Drosophila) /// suppressor of zeste 12 homolog pseudogene	0.001959	0.669931	-0.57792
213367_at	LOC155060	AI894139 pseudogene	0.001495	1.46337	0.549295
213510_x_at	LOC220594	ubiquitin specific peptidase 6 (Tre-2 oncogene) pseudogene	0.008725	0.667929	-0.58223
202651_at	LPGAT1	lysophosphatidylglycerol acyltransferase 1	0.008703	0.540574	-0.88744
212692_s_at	LRBA	LPS-responsive vesicle trafficking, beach and anchor containing	0.044736	1.33997	0.422198
218577_at	LRRC40	leucine rich repeat containing 40	0.000348	0.596395	-0.74566
212904_at	LRRC47	leucine rich repeat containing 47	0.037669	0.661323	-0.59657
206483_at	LRRC6	leucine rich repeat containing 6	0.024004	1.46376	0.549683
212978_at	LRRC8B	leucine rich repeat containing 8 family, member B	0.030916	0.689116	-0.53718
211747_s_at	LSM5	LSM5 homolog, U6 small nuclear RNA associated (S. cerevisiae)	0.048558	0.729766	-0.4545
205036_at	LSM6	LSM6 homolog, U6 small nuclear RNA associated (S. cerevisiae)	0.036402	0.761191	-0.39367
202729_s_at	LTBP1	latent transforming growth factor beta binding protein 1	0.002581	2.56994	1.36174
215596_s_at	LTN1	listerin E3 ubiquitin protein ligase 1	0.013627	0.679372	-0.55773
220044_x_at	LUC7L3	LUC7-like 3 (S. cerevisiae)	0.006947	0.717816	-0.47831
203007_x_at	LYPLA1	lysophospholipase I	0.004902	0.632945	-0.65985
218437_s_at	LZTFL1	leucine zipper transcription factor-like 1	0.037902	0.676447	-0.56395
220945_x_at	MANSC1	MANSC domain containing 1	0.011096	0.578456	-0.78972
214577_at	MAP1B	microtubule-associated protein 1B	0.000741	0.38412	-1.38037
202670_at	MAP2K1	mitogen-activated protein kinase kinase 1	0.028268	0.627648	-0.67197
211536_x_at	MAP3K7	mitogen-activated protein kinase kinase kinase 7	0.0047	1.36876	0.452869
211499_s_at	MAPK11	mitogen-activated protein kinase 11	0.001505	1.74277	0.801382
203218_at	MAPK9	mitogen-activated protein kinase 9	0.001041	0.657621	-0.60467
203928_x_at	MAPT	microtubule-associated protein tau	0.045653	0.566005	-0.82111
201669_s_at	MARCKS	myristoylated alanine-rich protein kinase C substrate	0.043405	0.757911	-0.3999
201151_s_at	MBNL1	muscleblind-like (Drosophila)	0.015491	0.513149	-0.96255
211042_x_at	MCAM	melanoma cell adhesion molecule	0.037515	0.62506	-0.67793
200978_at	MDH1	malate dehydrogenase 1, NAD (soluble)	0.047025	0.655601	-0.60911
218165_at	MEAF6	MYST/Esa1-associated factor 6	0.012112	0.726772	-0.46043
203506_s_at	MED12	mediator complex subunit 12	0.002999	1.50799	0.592627
43544_at	MED16	mediator complex subunit 16	0.0281	1.76666	0.821027
217843_s_at	MED4	mediator complex subunit 4	0.013381	0.611079	-0.71057
212535_at	MEF2A	myocyte enhancer factor 2A	0.012469	0.746736	-0.42133
207968_s_at	MEF2C	myocyte enhancer factor 2C	0.026272	0.594102	-0.75122
212732_at	MEG3	maternally expressed 3 (non-protein coding)	0.020953	0.594239	-0.75089
221570_s_at	METTL5	methyltransferase like 5	0.017178	0.691853	-0.53146
216205_s_at	MFN2	mitofusin 2	0.034591	0.757007	-0.40162
203636_at	MID1	midline 1 (Opitz/BBB syndrome)	0.048673	2.49785	1.32069
209241_x_at	MINK1	misshapen-like kinase 1	0.030308	1.30265	0.38145
217408_at	mitochondrial ribosomal protein S18B	chr6:30585628-30594168 (+) // 80.58 // p21.33	0.026469	0.50434	-0.98753
209845_at	MKRN1	makorin ring finger protein 1	0.030634	0.615414	-0.70037
213395_at	MLC1	megalencephalic leukoencephalopathy with subcortical cysts 1	0.009647	1.98941	0.992337

219703_at	MNS1	meiosis-specific nuclear structural 1	0.009465	1.76774	0.821908
212508_at	MOAP1	modulator of apoptosis 1	0.010855	0.643117	-0.63685
219959_at	MOCOS	molybdenum cofactor sulfurase	0.03387	0.644776	-0.63313
221381_s_at	MORF4 /// MORF4L1	mortality factor 4 /// mortality factor 4 like 1	0.00814	0.687777	-0.53999
205079_s_at	MPDZ	multiple PDZ domain protein	0.003857	0.635934	-0.65305
221771_s_at	MPHOSPH8	M-phase phosphoprotein 8	0.025231	0.516826	-0.95225
215731_s_at	MPHOSPH9	M-phase phosphoprotein 9	0.024773	2.12684	1.08871
202974_at	MPP1	membrane protein, palmitoylated 1, 55kDa	0.0192	0.729597	-0.45483
219321_at	MPP5	membrane protein, palmitoylated 5 (MAGUK p55 subfamily member 5)	0.043041	0.57815	-0.79049
219648_at	MREG	melanoregulin	0.003854	0.553384	-0.85365
203781_at	MRPL33	mitochondrial ribosomal protein L33	0.000821	0.599227	-0.73883
218105_s_at	MRPL4	mitochondrial ribosomal protein L4	0.001492	1.52591	0.609666
218654_s_at	MRPS33	mitochondrial ribosomal protein S33	0.034768	0.499115	-1.00255
212859_x_at	MT1E	metallothionein 1E	0.000493	2.26904	1.18208
213629_x_at	MT1F	metallothionein 1F	0.000935	1.87292	0.905286
204745_x_at	MT1G	metallothionein 1G	0.003128	2.14961	1.10407
206461_x_at	MT1H	metallothionein 1H	0.001901	2.13788	1.09618
211456_x_at	MT1P2	metallothionein 1 pseudogene 2	0.000955	1.81047	0.856362
204326_x_at	MT1X	metallothionein 1X	0.036068	1.67329	0.74269
212185_x_at	MT2A	metallothionein 2A	0.002344	1.74622	0.804238
205970_at	MT3	metallothionein 3	0.003773	1.58557	0.665005
212248_at	MTDH	metadherin	0.011978	0.752562	-0.41012
203345_s_at	MTF2	metal response element binding transcription factor 2	0.028509	0.706548	-0.50114
205076_s_at	MTMR11	myotubularin related protein 11	0.00148	1.66902	0.739003
203211_s_at	MTMR2	myotubularin related protein 2	0.004361	0.526433	-0.92568
214429_at	MTMR6	myotubularin related protein 6	0.00608	0.577044	-0.79325
222014_x_at	MTO1	mitochondrial translation optimization 1 homolog (S. cerevisiae)	0.007229	0.615916	-0.69919
203517_at	MTX2	metaxin 2	0.030951	0.725815	-0.46233
205018_s_at	muscleblind-like 2 (Drosophila)	chr13:97874607-98044254 (+) // 99.88 // q32.1	0.046548	0.629522	-0.66767
201959_s_at	MYCBP2	MYC binding protein 2	0.046206	0.484366	-1.04583
202258_s_at	N4BP2L2	NEDD4 binding protein 2-like 2	0.00194	0.530914	-0.91345
222161_at	NAALAD2	N-acetylated alpha-linked acidic dipeptidase 2	0.011842	1.90472	0.929577
217884_at	N-acetyltransferase 10 (GCN5-related)	chr11:34127148-34168457 (+) // 99.9 // p13	0.014624	1.32253	0.403304
208753_s_at	NAP1L1	nucleosome assembly protein 1-like 1	0.003121	0.510459	-0.97013
219368_at	NAP1L2	nucleosome assembly protein 1-like 2	0.033251	0.615992	-0.69902
204749_at	NAP1L3	nucleosome assembly protein 1-like 3	0.016601	0.684127	-0.54766
204725_s_at	NCK1	NCK adaptor protein 1	0.047683	0.724534	-0.46488
207738_s_at	NCKAP1	NCK-associated protein 1	0.006798	0.723666	-0.4666
217800_s_at	NDFIP1	Nedd4 family interacting protein 1	0.033421	0.73114	-0.45178
201304_at	NDUFA5	NADH dehydrogenase (ubiquinone) 1 alpha subcomplex, 5, 13kDa	0.001389	0.465367	-1.10356
219006_at	NDUFAF4	NADH dehydrogenase (ubiquinone) 1 alpha subcomplex, assembly factor 4	0.003685	0.636929	-0.6508
216882_s_at	NEBL	nebulette	0.037533	1.30759	0.386911
33767_at	NEFH	neurofilament, heavy polypeptide	0.020312	0.51196	-0.9659
221916_at	NEFL	neurofilament, light polypeptide	0.028863	0.517752	-0.94967
205113_at	NEFM	neurofilament, medium polypeptide	0.011123	0.442541	-1.17612
216096_s_at	neurexin 1	chr2:50148149-50574885 (-) // 97.39 // p16.3	0.002937	0.581155	-0.78301
220045_at	NEUROD6	neurogenic differentiation 6	0.043288	0.585794	-0.77153
217526_at	NFATC2IP	nuclear factor of activated T-cells, cytoplasmic, calcineurin-dependent 2 interacting protein	0.005515	0.429833	-1.21815
221104_s_at	NIPSNAP3B	nipsnap homolog 3B (C. elegans)	0.009303	0.677638	-0.56141
205004_at	NKRF	NFKB repressing factor	0.041745	0.765679	-0.38519
205893_at	NLGN1	neuroligin 1	0.001434	0.687795	-0.53995
221933_at	NLGN4X	neuroligin 4, X-linked	0.008678	0.751481	-0.41219
207703_at	NLGN4Y	neuroligin 4, Y-linked	0.045415	0.523402	-0.93401
218318_s_at	NLK	nemo-like kinase	0.000898	0.589506	-0.76242
215743_at	NMT2	N-myristoyltransferase 2	0.023447	0.589416	-0.76264
202783_at	NNT	nicotinamide nucleotide transhydrogenase	0.017336	0.646555	-0.62915
209798_at	NPAT	nuclear protein, ataxia-telangiectasia locus	0.003818	0.677037	-0.56269

89476_r_at	NPEPL1	aminopeptidase-like 1	0.0236	2.3844	1.25363
201454_s_at	NPEPPS	aminopeptidase puromycin sensitive	0.000356	0.542644	-0.88192
202228_s_at	NPTN	neuroplastin	0.034221	0.707448	-0.4993
216321_s_at	NR3C1	nuclear receptor subfamily 3, group C, member 1 (glucocorticoid receptor)	0.028399	0.739002	-0.43635
216959_x_at	NRCAM	neuronal cell adhesion molecule	0.016853	0.662672	-0.59363
219557_s_at	NRIP3	nuclear receptor interacting protein 3	0.032252	0.57308	-0.80319
218625_at	NRN1	neuritin 1	0.013065	0.657928	-0.604
209915_s_at	NRXN1	neurexin 1	0.037787	0.683577	-0.54882
211376_s_at	NSMCE4A	non-SMC element 4 homolog A (S. cerevisiae)	0.017213	1.47536	0.561064
221796_at	NTRK2	neurotrophic tyrosine kinase, receptor, type 2	0.034777	1.86359	0.898085
218227_at	NUBP2	nucleotide binding protein 2 (MinD homolog, E. coli)	0.023281	0.476383	-1.06981
213029_at	nuclear factor I/B	chr9:14081847-14083499 (-) // 90.03 // p23	0.018429	1.30091	0.379523
206302_s_at	NUDT4 /// NUDT4P1	nudix (nucleoside diphosphate linked moiety X)-type motif 4 /// nudix (nucleoside diphosphate linked moiety X)-type motif 4 pseudogene 1	0.021574	0.612705	-0.70674
200747_s_at	NUMA1	nuclear mitotic apparatus protein 1	0.00885	0.351351	-1.50901
207545_s_at	NUMB	numb homolog (Drosophila)	0.004177	2.21673	1.14843
202097_at	NUP153	nucleoporin 153kDa	0.017786	0.671872	-0.57374
215207_x_at	NUS1 /// NUS1P3	nuclear undecaprenyl pyrophosphate synthase 1 homolog (S. cerevisiae) /// nuclear undecaprenyl pyrophosphate synthase 1 homolog (S. cerevisiae) pseudogene 3	0.00995	0.399651	-1.32319
208922_s_at	NXF1	nuclear RNA export factor 1	0.035026	1.30094	0.379551
214111_at	OPCML	opioid binding protein/cell adhesion molecule-like	0.03867	0.61924	-0.69143
202073_at	optineurin	chr10:13142209-13180308 (+) // 85.94 // p13	0.028321	0.670104	-0.57754
218556_at	ORMDL2	ORM1-like 2 (S. cerevisiae)	0.007022	1.9727	0.980172
215399_s_at	OS9	osteosarcoma amplified 9, endoplasmic reticulum lectin	0.008154	1.46156	0.547508
209485_s_at	OSBPL1A	oxysterol binding protein-like 1A	0.011917	0.686678	-0.5423
212585_at	OSBPL8	oxysterol binding protein-like 8	0.005013	0.732019	-0.45005
218197_s_at	OXR1	oxidation resistance 1	0.000528	0.449208	-1.15454
212582_at	oxysterol binding protein-like 8	chr12:76745576-76783574 (-) // 98.46 // q21.2	0.043303	0.614271	-0.70305
211547_s_at	PAFAH1B1	platelet-activating factor acetylhydrolase 1b, regulatory subunit 1 (45kDa)	0.000987	0.462952	-1.11107
208051_s_at	PAIP1	poly(A) binding protein interacting protein 1	0.02206	0.607529	-0.71898
33814_at	PAK4	p21 protein (Cdc42/Rac)-activated kinase 4	0.032449	1.73395	0.794065
200907_s_at	PALLD	palladin, cytoskeletal associated protein	0.04561	1.54327	0.625992
214620_x_at	PAM	peptidylglycine alpha-amidating monooxygenase	0.043451	0.5086	-0.9754
221751_at	PANK3	pantothenate kinase 3	0.002764	0.748765	-0.41742
218543_s_at	PARP12	poly (ADP-ribose) polymerase family, member 12	0.00575	1.42189	0.507815
37966_at	PARVB	parvin, beta	0.008854	1.55936	0.64095
207867_at	PAX4	paired box 4	0.023513	1.67064	0.740398
214177_s_at	PBXIP1	pre-B-cell leukemia homeobox interacting protein 1	0.004116	1.80414	0.851312
205202_at	PCMT1	protein-L-isoaspartate (D-aspartate) O-methyltransferase	0.005166	0.633871	-0.65774
205549_at	PCP4	Purkinje cell protein 4	0.026964	0.509936	-0.97161
204869_at	PCSK2	proprotein convertase subtilisin/kexin type 2	0.001916	0.570044	-0.81086
203803_at	PCYOX1	prenylcysteine oxidase 1	0.002761	0.447524	-1.15996
210907_s_at	PDCD10	programmed cell death 10	0.037228	0.769138	-0.37869
208612_at	PDIA3	protein disulfide isomerase family A, member 3	0.034591	0.574681	-0.79917
208638_at	PDIA6	protein disulfide isomerase family A, member 6	0.020397	0.768573	-0.37975
219165_at	PDLIM2	PDZ and LIM domain 2 (mystique)	0.041417	1.64114	0.714701

218273_s_at	PDP1	pyruvate dehyrogenase phosphatase catalytic subunit 1	0.011067	0.495902	-1.01187
207956_x_at	PDS5B	PDS5, regulator of cohesion maintenance, homolog B (S. cerevisiae)	0.003704	0.638212	-0.64789
212915_at	PDZRN3	PDZ domain containing ring finger 3	0.022618	0.632592	-0.66065
220595_at	PDZRN4	PDZ domain containing ring finger 4	0.019828	0.665585	-0.58731
209243_s_at	PEG3	paternally expressed 3	0.023121	0.562118	-0.83106
215354_s_at	PELP1	proline, glutamate and leucine rich protein 1	0.031372	1.5651	0.646254
203970_s_at	PEX3	peroxisomal biogenesis factor 3	0.024643	0.726097	-0.46177
211033_s_at	PEX7	peroxisomal biogenesis factor 7	0.033405	1.34089	0.423195
205361_s_at	PFDN4	prefoldin subunit 4	0.006609	0.648612	-0.62457
217356_s_at	PGK1	phosphoglycerate kinase 1	0.017047	0.698011	-0.51868
213227_at	PGRMC2	progesterone receptor membrane component 2	0.038342	0.708806	-0.49654
213388_at	phosphodiesterase 4D interacting protein	chr1:144898004-144901433 (-) // 95.9 // q21.1 /// chr1:146507937-146511311 (-) // 90.21 // q21.1	0.049012	1.35677	0.440181
221689_s_at	PIGP	phosphatidylinositol glycan anchor biosynthesis, class P	0.035507	0.647683	-0.62664
204297_at	PIK3C3	phosphoinositide-3-kinase, class 3	0.019	0.421871	-1.24513
212688_at	PIK3CB	phosphoinositide-3-kinase, catalytic, beta polypeptide	0.025411	0.717871	-0.4782
212239_at	PIK3R1	phosphoinositide-3-kinase, regulatory subunit 1 (alpha)	0.018762	0.764741	-0.38696
209193_at	PIM1	pim-1 oncogene	0.03995	1.47597	0.561666
205632_s_at	PIP5K1B	phosphatidylinositol-4-phosphate 5-kinase, type I, beta	0.046865	0.715998	-0.48197
201190_s_at	PITPNA	phosphatidylinositol transfer protein, alpha	0.021406	1.3736	0.457966
219155_at	PITPNC1	phosphatidylinositol transfer protein, cytoplasmic 1	0.004249	1.62012	0.696098
201133_s_at	PJA2	praja ring finger 2	2.13E-05	0.609195	-0.71502
210145_at	PLA2G4A	phospholipase A2, group IVA (cytosolic, calcium-dependent)	0.030793	0.535726	-0.90043
206178_at	PLA2G5	phospholipase A2, group V	0.033646	1.52163	0.605621
220952_s_at	PLEKHA5	pleckstrin homology domain containing, family A member 5	0.003615	0.585927	-0.77121
201411_s_at	PLEKHB2	pleckstrin homology domain containing, family B (evectins) member 2	0.003248	0.441757	-1.17868
218224_at	PNMA1	paraneoplastic antigen MA1	0.001995	0.603873	-0.72768
209598_at	PNMA2	paraneoplastic antigen MA2	0.024741	0.7665	-0.38364
201695_s_at	PNP	purine nucleoside phosphorylase	0.002669	1.60502	0.682587
209578_s_at	POFUT2	protein O-fucosyltransferase 2	0.003442	2.6418	1.40152
213325_at	poliovirus receptor-related 3	chr3:110790512-110854797 (+) // 91.64 // q13.13	0.024403	0.639469	-0.64505
207515_s_at	POLR1C	polymerase (RNA) I polypeptide C, 30kDa	4.15E-05	1.36893	0.453047
218258_at	POLR1D	polymerase (RNA) I polypeptide D, 16kDa	0.041834	0.724509	-0.46493
210573_s_at	POLR3C	polymerase (RNA) III (DNA directed) polypeptide C (62kD)	0.005022	2.43129	1.28172
209064_x_at	poly(A) binding protein interacting protein 1	chr17:18553507-18556020 (+) // 87.87 // p11.2 /// chr5:43526368-43557142 (-) // 98.37 // p12	0.042171	0.73499	-0.4442
208828_at	polymerase (DNA directed), epsilon 3 (p17 subunit)	chr9:116169520-116172644 (-) // 80.8 // q32	0.026786	0.700262	-0.51403
213469_at	post-GPI attachment to proteins 1	chr2:197700305-197705337 (-) // 81.14 // q33.1	0.044143	0.70466	-0.505
220741_s_at	PPA2	pyrophosphatase (inorganic) 2	0.011171	0.755478	-0.40454
212230_at	PPAP2B	phosphatidic acid phosphatase type 2B	0.012576	1.90573	0.930341
210235_s_at	PPFIA1	protein tyrosine phosphatase, receptor type, f polypeptide (PTPRF), interacting protein (liprin), alpha 1	0.000586	1.56953	0.650337
209296_at	PPM1B	protein phosphatase, Mg2+/Mn2+ dependent, 1B	0.004156	0.624377	-0.67951
205938_at	PPM1E	protein phosphatase, Mg2+/Mn2+ dependent, 1E	0.030804	0.509679	-0.97234
37384_at	PPM1F	protein phosphatase, Mg2+/Mn2+ dependent, 1F	0.00412	1.47079	0.556587

200913_at	PPM1G	protein phosphatase, Mg2+/Mn2+ dependent, 1G	0.001259	1.57207	0.652665
212686_at	PPM1H	protein phosphatase, Mg2+/Mn2+ dependent, 1H	0.009036	0.639975	-0.64391
201409_s_at	PPP1CB	protein phosphatase 1, catalytic subunit, beta isozyme	0.049532	0.719997	-0.47394
201603_at	PPP1R12A	protein phosphatase 1, regulatory (inhibitor) subunit 12A	0.012908	0.664691	-0.58924
216347_s_at	PPP1R13B	protein phosphatase 1, regulatory (inhibitor) subunit 13B	0.008383	2.0153	1.011
37028_at	PPP1R15A	protein phosphatase 1, regulatory (inhibitor) subunit 15A	0.012442	2.10649	1.07484
202166_s_at	PPP1R2	protein phosphatase 1, regulatory (inhibitor) subunit 2	0.03299	0.725777	-0.4624
203338_at	PPP2R5E	protein phosphatase 2, regulatory subunit B', epsilon isoform	0.013142	0.654162	-0.61228
202457_s_at	PPP3CA	protein phosphatase 3, catalytic subunit, alpha isozyme	0.009765	0.693333	-0.52838
212216_at	PREPL	prolyl endopeptidase-like	0.007165	0.515208	-0.95677
202742_s_at	PRKACB	protein kinase, cAMP-dependent, catalytic, beta	0.001213	0.539853	-0.88936
200604_s_at	PRKAR1A	protein kinase, cAMP-dependent, regulatory, type I, alpha (tissue specific extinguisher 1)	0.029829	0.700529	-0.51348
203680_at	PRKAR2B	protein kinase, cAMP-dependent, regulatory, type II, beta	0.011557	0.634415	-0.6565
207957_s_at	PRKCB	protein kinase C, beta	0.01507	0.562662	-0.82966
209139_s_at	PRKRA	protein kinase, interferon-inducible double stranded RNA dependent activator	0.011624	0.754141	-0.4071
209323_at	PRKRIR	protein-kinase, interferon-inducible double stranded RNA dependent inhibitor, repressor of (P58 repressor)	0.006527	0.757591	-0.40051
217786_at	PRMT5	protein arginine methyltransferase 5	0.022401	0.481032	-1.05579
209385_s_at	proline synthetase co-transcribed homolog (bacterial)	chr8:37620124-37636435 (+) // 84.76 // p11.23	0.008426	0.665729	-0.58699
38269_at	protein kinase D2	chr19:47177572-47204203 (-) // 84.45 // q13.32	0.000948	1.51966	0.603748
200603_at	protein kinase, cAMP-dependent, regulatory, type I, alpha (tissue specific extinguisher 1)	chr17:66508579-66529568 (+) // 97.56 // q24.2	0.000696	0.679795	-0.55683
215172_at	protein tyrosine phosphatase, non-receptor type 20A /// protein tyrosine phosphatase, non-receptor type 20B	chr10:46550124-46641003 (-) // 98.6 // q11.22 /// chr10:48737043-48827924 (-) // 98.6 // q11.22	0.024332	0.604168	-0.72698
213362_at	protein tyrosine phosphatase, receptor type, D	chr9:8314915-8317287 (-) // 92.75 // p24.1	0.048802	0.501373	-0.99604
221547_at	PRPF18	PRP18 pre-mRNA processing factor 18 homolog (S. cerevisiae)	0.014766	0.637997	-0.64838
220553_s_at	PRPF39	PRP39 pre-mRNA processing factor 39 homolog (S. cerevisiae)	0.03229	0.731895	-0.45029
208880_s_at	PRPF6	PRP6 pre-mRNA processing factor 6 homolog (S. cerevisiae)	0.036973	1.37204	0.456328
47069_at	PRR5	proline rich 5 (renal)	0.001299	1.46555	0.551439
205618_at	PRRG1	proline rich Gla (G-carboxyglutamic acid) 1	0.036035	0.546309	-0.87221
212806_at	PRUNE2	prune homolog 2 (Drosophila)	0.005401	0.580349	-0.78501
201532_at	PSMA3	proteasome (prosome, macropain) subunit, alpha type, 3	0.016549	0.706948	-0.50033
204279_at	PSMB9	proteasome (prosome, macropain) subunit, beta type, 9 (large multifunctional peptidase 2)	0.002103	1.44774	0.533807
201067_at	PSMC2	proteasome (prosome, macropain) 26S subunit, ATPase, 2	0.011184	0.439751	-1.18524
202353_s_at	PSMD12	proteasome (prosome, macropain) 26S	0.015764	0.674627	-0.56784

		subunit, non-ATPase, 12			
205194_at	PSPH	phosphoserine phosphatase	0.01678	0.698673	-0.51731
206772_at	PTH2R	parathyroid hormone 2 receptor	0.036147	0.598025	-0.74172
218045_x_at	PTMS	parathymosin	0.036385	2.3808	1.25144
201493_s_at	PUM2	pumilio homolog 2 (Drosophila)	0.000536	0.667285	-0.58362
205336_at	PVALB	parvalbumin	0.005058	0.761394	-0.39329
201606_s_at	PWP1	PWP1 homolog (S. cerevisiae)	0.001561	0.539248	-0.89098
212012_at	PXDN	peroxidasin homolog (Drosophila)	0.036583	1.31224	0.392035
203884_s_at	RAB11FIP2	RAB11 family interacting protein 2 (class I)	0.041728	0.705875	-0.50252
211503_s_at	RAB14	RAB14, member RAS oncogene family	0.00964	0.55202	-0.85721
207791_s_at	RAB1A	RAB1A, member RAS oncogene family	0.024781	0.611789	-0.70889
208731_at	RAB2A	RAB2A, member RAS oncogene family	1.96E-07	0.714124	-0.48575
206039_at	RAB33A	RAB33A, member RAS oncogene family	0.01917	0.596376	-0.74571
213530_at	RAB3GAP1	RAB3 GTPase activating protein subunit 1 (catalytic)	0.007133	0.517699	-0.94982
204547_at	RAB40B	RAB40B, member RAS oncogene family	0.0051	0.707718	-0.49875
203581_at	RAB4A	RAB4A, member RAS oncogene family	0.007257	0.724584	-0.46478
210406_s_at	RAB6A, member RAS oncogene family /// RAB6C, member RAS oncogene family	chr2:130737236-130740311 (+) // 98.96 // q21.1 /// chr11:73386940-73472121 (-) // 93.13 // q13.4 /// chr2:132118687-132121731 (-) // 96.53 // q21.1	0.001255	0.688961	-0.53751
74694_s_at	RABEP2	rabaptin, RAB GTPase binding effector protein 2	0.042017	1.637	0.711054
215342_s_at	RABGAP1L	RAB GTPase activating protein 1-like	0.043705	0.373545	-1.42065
204461_x_at	RAD1	RAD1 homolog (S. pombe)	0.007442	0.602178	-0.73174
201222_s_at	RAD23B	RAD23 homolog B (S. cerevisiae)	0.005303	0.63829	-0.64772
206066_s_at	RAD51C	RAD51 homolog C (S. cerevisiae)	0.024988	0.63869	-0.64681
205130_at	RAGE	renal tumor antigen	7.31E-05	1.90191	0.927447
214855_s_at	Ral GTPase activating protein, alpha subunit 1 (catalytic)	chr9:108287492-108289605 (+) // 96.52 // q31.2 /// chr14:36007953-36096675 (-) // 96.38 // q13.2	0.049826	0.695893	-0.52306
209110_s_at	ral guanine nucleotide dissociation stimulator-like 2	chr6:33259437-33266738 (-) // 98.66 // p21.32	0.005168	1.33291	0.414577
201711_x_at	RANBP2	RAN binding protein 2	0.020954	0.644523	-0.6337
202582_s_at	RANBP9	RAN binding protein 9	0.001582	0.660409	-0.59857
217457_s_at	RAP1GDS1	RAP1, GTP-GDP dissociation stimulator 1	0.020789	0.640323	-0.64313
210621_s_at	RASA1	RAS p21 protein activator (GTPase activating protein) 1	0.013765	0.701886	-0.51069
205590_at	RASGRP1	RAS guanyl releasing protein 1 (calcium and DAG-regulated)	0.006568	0.544129	-0.87798
202034_x_at	RB1CC1	RB1-inducible coiled-coil 1	0.016584	0.672646	-0.57208
212027_at	RBM25	RNA binding motif protein 25	0.034701	0.409872	-1.28675
206767_at	RBMS3	RNA binding motif, single stranded interacting protein 3	0.015326	1.58802	0.66723
212749_s_at	RCHY1	ring finger and CHY zinc finger domain containing 1	0.000815	0.672926	-0.57148
201486_at	RCN2	reticulocalbin 2, EF-hand calcium binding domain	0.005339	0.58644	-0.76994
217776_at	RDH11	retinol dehydrogenase 11 (all-trans/9-cis/11-cis)	0.038444	0.730551	-0.45294
222203_s_at	RDH14	retinol dehydrogenase 14 (all-trans/9-cis/11-cis)	0.010862	0.694587	-0.52577
204364_s_at	REEP1	receptor accessory protein 1	0.020988	0.633605	-0.65834
208873_s_at	REEP5	receptor accessory protein 5	0.005422	0.763252	-0.38977
201783_s_at	RELA	v-rel reticuloendotheliosis viral oncogene homolog A (avian)	0.00658	1.3785	0.463102
203225_s_at	RFK	riboflavin kinase	0.027178	0.700311	-0.51393
203169_at	RGP1	RGP1 retrograde golgi transport homolog (S. cerevisiae)	0.041143	0.761319	-0.39343
212842_x_at	RGPD4 /// RGPD5 /// RGPD6 /// RGPD8	RANBP2-like and GRIP domain containing 4 /// RANBP2-like and GRIP domain containing 5 /// RANBP2-like and GRIP domain containing 6 /// RANBP2-like and GRIP domain containing 8	0.018385	0.749408	-0.41618
210138_at	RGS20	regulator of G-protein signaling 20	0.022002	1.61471	0.691271

204338_s_at	RGS4	regulator of G-protein signaling 4	0.033534	0.263514	-1.92405
206290_s_at	RGS7	regulator of G-protein signaling 7	0.044535	0.715894	-0.48218
200885_at	RHOC	ras homolog gene family, member C	0.046245	1.87085	0.903695
219045_at	RHOF	ras homolog gene family, member F (in filopodia)	0.026382	1.85891	0.894458
222148_s_at	RHOT1	ras homolog gene family, member T1	0.026943	0.756533	-0.40253
219446_at	RIC8B	resistance to inhibitors of cholinesterase 8 homolog B (C. elegans)	0.022939	1.38856	0.473587
206137_at	RIMS2	regulating synaptic membrane exocytosis 2	0.013194	0.580781	-0.78394
202130_at	RIOK3	RIO kinase 3 (yeast)	0.044521	0.752868	-0.40953
206984_s_at	RIT2	Ras-like without CAAX 2	0.021314	0.460187	-1.11971
203022_at	RNASEH2A	ribonuclease H2, subunit A	0.044249	1.35328	0.436457
213467_at	RND2	Rho family GTPase 2	0.043755	1.75476	0.811275
208924_at	RNF11	ring finger protein 11	0.002741	0.690611	-0.53406
218761_at	RNF111	ring finger protein 111	0.004615	0.670223	-0.57729
209111_at	RNF5	ring finger protein 5	0.038579	0.756396	-0.40279
203403_s_at	RNF6	ring finger protein (C3H2C3 type) 6	0.004075	0.690877	-0.5335
218286_s_at	RNF7	ring finger protein 7	0.039954	0.71262	-0.48879
204208_at	RNGTT	RNA guanylyltransferase and 5'-phosphatase	0.023191	0.456795	-1.13038
201529_s_at	RPA1	replication protein A1, 70kDa	0.03691	0.47805	-1.06477
208929_x_at	RPL13	ribosomal protein L13	0.006373	1.41188	0.497614
200869_at	RPL18A /// RPL18AP3	ribosomal protein L18a /// ribosomal protein L18a pseudogene 3	0.021554	1.30944	0.388948
213969_x_at	RPL29	ribosomal protein L29	0.004449	1.35268	0.435816
218209_s_at	RPRD1A	regulation of nuclear pre-mRNA domain containing 1A	0.01537	0.651063	-0.61913
217915_s_at	RSL24D1	ribosomal L24 domain containing 1	0.003725	0.599638	-0.73784
203594_at	RTCD1	RNA terminal phosphate cyclase domain 1	0.021934	0.481224	-1.05522
214629_x_at	RTN4	reticulon 4	0.004992	0.762644	-0.39092
213939_s_at	RUFY3	RUN and FYVE domain containing 3	0.021115	0.741757	-0.43098
219598_s_at	RWDD1	RWD domain containing 1	0.008106	0.684337	-0.54722
201845_s_at	RYBP	RING1 and YY1 binding protein	0.033014	0.744238	-0.42616
214044_at	RYR2	ryanodine receptor 2 (cardiac)	0.017258	0.64688	-0.62843
205334_at	S100A1	S100 calcium binding protein A1	0.03249	1.72397	0.785737
218677_at	S100A14	S100 calcium binding protein A14	0.035181	1.47475	0.560473
202797_at	SACM1L	SAC1 suppressor of actin mutations 1-like (yeast)	0.014711	0.677343	-0.56204
213262_at	SACS	spastic ataxia of Charlevoix-Saguenay (sacsin)	0.004604	0.676193	-0.56449
200051_at	SART1	squamous cell carcinoma antigen recognized by T cells	0.049474	1.37228	0.456579
200069_at	SART3	squamous cell carcinoma antigen recognized by T cells 3	0.021036	0.746156	-0.42245
206668_s_at	SCAMP1	secretory carrier membrane protein 1	0.020885	0.61467	-0.70212
209741_x_at	SCAPER	S-phase cyclin A-associated protein in the ER	0.013056	0.639036	-0.64603
219416_at	SCARA3	scavenger receptor class A, member 3	0.012207	3.46444	1.79262
201646_at	SCARB2	scavenger receptor class B, member 2	0.008869	0.589826	-0.76164
215548_s_at	SCFD1	sec1 family domain containing 1	0.026743	0.766464	-0.38371
203889_at	SCG5	secretogranin V (7B2 protein)	0.03109	0.690215	-0.53488
210383_at	SCN1A	sodium channel, voltage-gated, type I, alpha subunit	0.000875	0.429585	-1.21898
206381_at	SCN2A	sodium channel, voltage-gated, type II, alpha subunit	0.001781	0.547724	-0.86848
205475_at	SCRG1	stimulator of chondrogenesis 1	0.030135	1.55739	0.639129
212158_at	SDC2	syndecan 2	0.030622	1.67139	0.741049
202071_at	SDC4	syndecan 4	0.029334	1.73751	0.79702
218649_x_at	SDCCAG1	serologically defined colon cancer antigen 1	0.034427	0.662694	-0.59358
215088_s_at	SDHC	succinate dehydrogenase complex, subunit C, integral membrane protein, 15kDa	0.024454	0.647051	-0.62805
212887_at	Sec23 homolog A (S. cerevisiae)	chr14:39501122-39510637 (-) // 84.96 // q21.1	0.002516	0.573247	-0.80277
202375_at	SEC24D	SEC24 family, member D (S. cerevisiae)	0.014686	0.514747	-0.95807
203484_at	SEC61G	Sec61 gamma subunit	0.023319	0.672389	-0.57263
208942_s_at	SEC62	SEC62 homolog (S. cerevisiae)	0.016972	0.658302	-0.60318
201915_at	SEC63	SEC63 homolog (S. cerevisiae)	0.003488	0.453217	-1.14172
218265_at	SECISBP2	SECIS binding protein 2	0.026095	0.739253	-0.43586
221931_s_at	SEH1L	SEH1-like (S. cerevisiae)	0.005653	0.662304	-0.59443

214433_s_at	SELENBP1	selenium binding protein 1	0.033585	1.42359	0.50953
200902_at	SEP15	15 kDa selenoprotein	0.011142	0.641726	-0.63997
210466_s_at	SERBP1	SERPINE1 mRNA binding protein 1	0.012924	0.671822	-0.57385
208671_at	SERINC1	serine incorporator 1	0.006027	0.729339	-0.45534
221471_at	SERINC3	serine incorporator 3	0.004722	0.673563	-0.57012
209723_at	SERPINB9	serpin peptidase inhibitor, clade B (ovalbumin), member 9	0.037213	0.561457	-0.83275
205352_at	SERPINI1	serpin peptidase inhibitor, clade I (neuroserpin), member 1	0.045421	0.62879	-0.66935
36545_s_at	SFI1	Sfi1 homolog, spindle assembly associated (yeast)	0.035583	1.75634	0.812573
201586_s_at	SFPQ	splicing factor proline/glutamine-rich	0.008615	0.63294	-0.65986
212176_at	SFRS18	splicing factor, arginine/serine-rich 18	0.020429	0.646873	-0.62845
201810_s_at	SH3BP5	SH3-domain binding protein 5 (BTK-associated)	0.002076	0.444433	-1.16996
205063_at	SIP1	survival of motor neuron protein interacting protein 1	0.045993	0.716135	-0.4817
206052_s_at	SLBP	stem-loop binding protein	0.048443	0.711815	-0.49043
210040_at	SLC12A5	solute carrier family 12 (potassium/chloride transporter), member 5	0.008736	0.62188	-0.68529
205856_at	SLC14A1	solute carrier family 14 (urea transporter), member 1 (Kidd blood group)	0.02631	2.37896	1.25033
205316_at	SLC15A2	solute carrier family 15 (H+/peptide transporter), member 2	0.037312	1.72598	0.787415
212810_s_at	SLC1A4	solute carrier family 1 (glutamate/neutral amino acid transporter), member 4	0.003486	1.6427	0.716066
201920_at	SLC20A1	solute carrier family 20 (phosphate transporter), member 1	0.004791	0.730226	-0.45359
219090_at	SLC24A3	solute carrier family 24 (sodium/potassium/calcium exchanger), member 3	0.015297	2.94619	1.55885
221020_s_at	SLC25A32	solute carrier family 25, member 32	0.042064	0.603348	-0.72894
201918_at	SLC25A36	solute carrier family 25, member 36	0.003468	0.505189	-0.98511
205716_at	SLC25A40	solute carrier family 25, member 40	0.001382	0.641985	-0.63939
202498_s_at	SLC2A3	solute carrier family 2 (facilitated glucose transporter), member 3	0.04463	0.585483	-0.7723
218519_at	SLC35A5	solute carrier family 35, member A5	0.048443	0.687133	-0.54134
218988_at	SLC35E3	solute carrier family 35, member E3	0.009936	0.475856	-1.0714
218041_x_at	SLC38A2	solute carrier family 38, member 2	0.007336	0.685584	-0.5446
202088_at	SLC39A6	solute carrier family 39 (zinc transporter), member 6	0.028426	0.756865	-0.40189
217859_s_at	SLC39A9	solute carrier family 39 (zinc transporter), member 9	0.028117	1.4014	0.486867
207056_s_at	SLC4A8	solute carrier family 4, sodium bicarbonate cotransporter, member 8	0.011115	0.453904	-1.13954
211123_at	SLC5A5	solute carrier family 5 (sodium iodide symporter), member 5	0.026562	1.95101	0.964223
201195_s_at	SLC7A5	solute carrier family 7 (cationic amino acid transporter, y+ system), member 5	0.032581	1.36437	0.44824
206732_at	SLITRK3	SLIT and NTRK-like family, member 3	0.032621	0.680063	-0.55626
206542_s_at	SMARCA2	SWI/SNF related, matrix associated, actin dependent regulator of chromatin, subfamily a, member 2	0.012768	0.753321	-0.40866
202303_x_at	SMARCA5	SWI/SNF related, matrix associated, actin dependent regulator of chromatin, subfamily a, member 5	0.024336	0.383409	-1.38304
201321_s_at	SMARCC2	SWI/SNF related, matrix associated, actin dependent regulator of chromatin, subfamily c, member 2	0.034507	0.6271	-0.67323
209518_at	SMARCD1	SWI/SNF related, matrix associated, actin dependent regulator of chromatin, subfamily d, member 1	0.035489	1.44465	0.530725
204099_at	SMARCD3	SWI/SNF related, matrix associated, actin dependent regulator of chromatin, subfamily d, member 3	0.034818	1.65905	0.73036
212569_at	SMCHD1	structural maintenance of chromosomes flexible hinge domain containing 1	0.006768	0.487507	-1.03651
200071_at	SMNDC1	survival motor neuron domain containing 1	0.022146	0.66243	-0.59416
213624_at	SMPDL3A	sphingomyelin phosphodiesterase, acid-like	0.022399	0.719283	-0.47537

		3A			
212922_s_at	SMYD2	SET and MYND domain containing 2	0.005421	0.507565	-0.97834
210465_s_at	SNAPC3	small nuclear RNA activating complex, polypeptide 3, 50kDa	0.001971	0.685161	-0.54549
202505_at	SNRPB2	small nuclear ribonucleoprotein polypeptide B	0.039203	0.754925	-0.4056
202690_s_at	SNRPD1	small nuclear ribonucleoprotein D1 polypeptide 16kDa	0.014127	0.723627	-0.46668
203516_at	SNTA1	syntrophin, alpha 1 (dystrophin-associated protein A1, 59kDa, acidic component)	0.017524	1.44096	0.527027
218404_at	SNX10	sorting nexin 10	0.005584	0.463699	-1.10874
202113_s_at	SNX2	sorting nexin 2	0.021766	0.664631	-0.58938
218974_at	SOBP	sine oculis binding protein homolog (Drosophila)	0.024824	0.747004	-0.42081
209647_s_at	SOCS5	suppressor of cytokine signaling 5	0.001282	0.67918	-0.55813
200657_at	solute carrier family 25 (mitochondrial carrier	chr7:32511042-32512610 (+) // 91.51 // p14.3 /// chr7:54487063-54488281 (+) // 89.88 // p11.2 /// chrX:118602408-118605279 (+) // 99.27 // q24 /// chr2:34064591-34065809 (-) // 90.94 // p22.3	0.006044	0.756726	-0.40216
201416_at	SOX4	SRY (sex determining region Y)-box 4	0.004485	2.01985	1.01425
210985_s_at	SP100	SP100 nuclear antigen	0.022351	1.61797	0.694187
209761_s_at	SP110	SP110 nuclear body protein	0.048367	1.38715	0.472123
210033_s_at	SPAG6	sperm associated antigen 6	0.03239	1.50048	0.585427
207724_s_at	spastin	chr2:32288775-32380852 (+) // 98.74 // p22.3	0.009486	0.72274	-0.46845
214965_at	SPATA2L	spermatogenesis associated 2-like	0.028174	1.58258	0.66228
212721_at	splicing regulatory glutamine/lysine-rich protein 1	chr5:65457941-65476712 (+) // 93.59 // q12.3	0.047347	0.646597	-0.62906
219677_at	SPSB1	splA/ryanodine receptor domain and SOCS box containing 1	0.014071	1.59483	0.673398
217608_at	SREK1IP1	SREK1-interacting protein 1	0.013776	0.505994	-0.98281
213329_at	SRGAP2	SLIT-ROBO Rho GTPase activating protein 2	0.035336	1.91671	0.938629
205335_s_at	SRP19	signal recognition particle 19kDa	0.005069	0.730788	-0.45248
208095_s_at	SRP72	signal recognition particle 72kDa	0.031031	0.502638	-0.99241
201273_s_at	SRP9	signal recognition particle 9kDa	0.005559	0.736868	-0.44052
202200_s_at	SRPK1	SRSF protein kinase 1	0.001587	0.681159	-0.55394
214882_s_at	SRSF2	serine/arginine-rich splicing factor 2	0.009281	0.701452	-0.51158
201129_at	SRSF7	serine/arginine-rich splicing factor 7	0.015216	0.742696	-0.42916
213140_s_at	SS18L1	synovial sarcoma translocation gene on chromosome 18-like 1	0.018819	0.722931	-0.46807
201138_s_at	SSB	Sjogren syndrome antigen B (autoantigen La)	0.021309	0.713425	-0.48717
202591_s_at	SSBP1	single-stranded DNA binding protein 1	0.00267	0.729907	-0.45422
210829_s_at	SSBP2	single-stranded DNA binding protein 2	0.034118	0.741829	-0.43084
203017_s_at	SSX2IP	synovial sarcoma, X breakpoint 2 interacting protein	0.019155	0.555597	-0.84789
201998_at	ST6GAL1	ST6 beta-galactosamide alpha-2,6-sialyltranferase 1	0.0297	0.634989	-0.6552
209023_s_at	STAG2	stromal antigen 2	0.025619	0.48385	-1.04737
AFFX-HUMISGF3A/M97935_3_at	STAT1	signal transducer and activator of transcription 1, 91kDa	0.028766	0.764107	-0.38815
205026_at	STAT5B	signal transducer and activator of transcription 5B	0.013852	1.54091	0.623779
205339_at	STIL	SCL/TAL1 interrupting locus	0.033244	1.44397	0.530044
40420_at	STK10	serine/threonine kinase 10	0.003615	1.6902	0.75719
202786_at	STK39	serine threonine kinase 39	0.000624	0.581179	-0.78295
204496_at	STRN3	striatin, calmodulin binding protein 3	0.019872	0.685872	-0.54399
221499_s_at	STX16	syntaxin 16	0.024435	0.740699	-0.43304
212631_at	STX7	syntaxin 7	0.021055	0.755675	-0.40416
202930_s_at	SUCLA2	succinate-CoA ligase, ADP-forming, beta subunit	0.001611	0.522872	-0.93547
211069_s_at	SUMO1	SMT3 suppressor of mif two 3 homolog 1 (S. cerevisiae)	0.00124	0.743824	-0.42697
201837_s_at	SUPT7L	suppressor of Ty 7 (S. cerevisiae)-like	0.035893	0.757426	-0.40082

212287_at	SUZ12	suppressor of zeste 12 homolog (Drosophila)	0.011255	0.711758	-0.49054
210315_at	SYN2	synapsin II	0.042022	0.471963	-1.08326
209025_s_at	SYNCRIP	synaptotagmin binding, cytoplasmic RNA interacting protein	0.045426	0.76306	-0.39013
212990_at	SYNJ1	synaptojanin 1	0.012316	0.564355	-0.82532
203998_s_at	SYT1	synaptotagmin I	0.006736	0.381279	-1.39108
221859_at	SYT13	synaptotagmin XIII	0.008838	1.43918	0.525243
206552_s_at	TAC1	tachykinin, precursor 1	0.01111	0.528917	-0.91889
209358_at	TAF11	TAF11 RNA polymerase II, TATA box binding protein (TBP)-associated factor, 28kDa	0.0195	0.758451	-0.39887
221580_s_at	TAF1D	TATA box binding protein (TBP)-associated factor, RNA polymerase I, D, 41kDa	0.008453	0.7235	-0.46694
201023_at	TAF7	TAF7 RNA polymerase II, TATA box binding protein (TBP)-associated factor, 55kDa	0.005906	0.719497	-0.47494
203893_at	TAF9	TAF9 RNA polymerase II, TATA box binding protein (TBP)-associated factor, 32kDa	0.035987	0.74765	-0.41957
206283_s_at	TAL1	T-cell acute lymphocytic leukemia 1	0.046305	1.37655	0.461062
201263_at	TARS	threonyl-tRNA synthetase	0.023138	0.721971	-0.46999
219443_at	TASP1	taspase, threonine aspartase, 1	0.013451	0.588164	-0.76571
213786_at	TAX1BP1	Tax1 (human T-cell leukemia virus type I) binding protein 1	0.01758	0.710029	-0.49405
212796_s_at	TBC1D2B	TBC1 domain family, member 2B	0.033473	1.39421	0.479449
203386_at	TBC1D4	TBC1 domain family, member 4	0.026944	1.37767	0.462227
206431_x_at	TBC1D9B	TBC1 domain family, member 9B (with GRAM domain)	0.004627	1.32039	0.400965
208398_s_at	TBPL1	TBP-like 1	0.020689	0.659109	-0.60141
220025_at	TBR1	T-box, brain, 1	0.017887	0.680085	-0.55621
204045_at	TCEAL1	transcription elongation factor A (SII)-like 1	0.017913	0.67692	-0.56294
202823_at	TCEB1	transcription elongation factor B (SIII), polypeptide 1 (15kDa, elongin C)	0.02527	0.583416	-0.7774
212382_at	TCF4	transcription factor 4	0.005619	0.459513	-1.12182
212762_s_at	TCF7L2	transcription factor 7-like 2 (T-cell specific, HMG-box)	0.030684	0.478112	-1.06458
202384_s_at	TCOF1	Treacher Collins-Franceschetti syndrome 1	0.00912	1.79733	0.845855
208778_s_at	TCP1	t-complex 1	0.003958	0.5602	-0.83599
205943_at	TDO2	tryptophan 2,3-dioxygenase	0.002235	1.35816	0.44165
208336_s_at	TECR	trans-2,3-enoyl-CoA reductase	0.029235	1.36721	0.451237
204106_at	TESK1	testis-specific kinase 1	0.005159	2.20342	1.13974
218605_at	TFB2M	transcription factor B2, mitochondrial	0.018914	0.68973	-0.5359
208691_at	TFRC	transferrin receptor (p90, CD71)	0.031012	1.46864	0.554486
221235_s_at	TGFBRAP1	transforming growth factor, beta receptor associated protein 1	0.04264	1.32509	0.40609
201042_at	TGM2	transglutaminase 2 (C polypeptide, protein-glutamine-gamma-glutamyltransferase)	0.003432	1.64132	0.714856
212040_at	TGOLN2	trans-golgi network protein 2	0.022699	0.556859	-0.84462
218334_at	THOC7	THO complex 7 homolog (Drosophila)	0.002335	0.639439	-0.64512
204100_at	THRA	thyroid hormone receptor, alpha (erythroblastic leukemia viral (v-erb-a) oncogene homolog, avian)	0.045594	1.83377	0.874809
213025_at	THUMPD1	THUMP domain containing 1	0.037764	0.685079	-0.54566
201450_s_at	TIA1	TIA1 cytotoxic granule-associated RNA binding protein	0.006136	0.446931	-1.16188
201821_s_at	TIMM17A	translocase of inner mitochondrial membrane 17 homolog A (yeast)	0.004053	0.424911	-1.23477
220052_s_at	TINF2	TERF1 (TRF1)-interacting nuclear factor 2	0.044387	2.01585	1.01139
204872_at	TLE4	transducin-like enhancer of split 4 (E(sp1) homolog, Drosophila)	0.012783	0.690545	-0.53419
202606_s_at	TLK1	tousled-like kinase 1	0.023876	0.66288	-0.59318
201078_at	TM9SF2	transmembrane 9 superfamily member 2	0.013967	0.767095	-0.38252
219206_x_at	TMBIM4	transmembrane BAX inhibitor motif containing 4	0.024838	0.671261	-0.57506
205123_s_at	TMEFF1	transmembrane protein with EGF-like and two follistatin-like domains 1	0.000402	0.476669	-1.06894
218930_s_at	TMEM106B	transmembrane protein 106B	0.012298	0.536868	-0.89736
212507_at	TMEM131	transmembrane protein 131	0.048399	0.745108	-0.42448
218531_at	TMEM134	transmembrane protein 134	0.044159	1.68817	0.755462
212164_at	TMEM183A	transmembrane protein 183A	0.028453	0.672953	-0.57142

217743_s_at	TMEM30A	transmembrane protein 30A	0.026566	0.707398	-0.49941
212623_at	TMEM41B	transmembrane protein 41B	0.034282	0.710355	-0.49339
212282_at	TMEM97	transmembrane protein 97	0.008094	0.523749	-0.93305
209499_x_at	TNFSF12-TNFSF13 /// TNFSF13	TNFSF12-TNFSF13 readthrough /// tumor necrosis factor (ligand) superfamily, member 13	0.043073	1.47876	0.56439
210314_x_at	TNFSF13	tumor necrosis factor (ligand) superfamily, member 13	0.010968	1.65868	0.730034
212773_s_at	TOMM20	translocase of outer mitochondrial membrane 20 homolog (yeast)	0.012344	0.550946	-0.86002
201512_s_at	TOMM70A	translocase of outer mitochondrial membrane 70 homolog A (S. cerevisiae)	0.042481	0.728491	-0.45702
202633_at	TOPBP1	topoisomerase (DNA) II binding protein 1	0.03021	0.735327	-0.44354
201688_s_at	TPD52	tumor protein D52	0.0021	0.476305	-1.07004
210372_s_at	TPD52L1	tumor protein D52-like 1	0.030026	1.49875	0.583761
203511_s_at	TRAPPC3	trafficking protein particle complex 3	0.039094	0.742533	-0.42947
219937_at	TRHDE	thyrotropin-releasing hormone degrading enzyme	0.023216	0.45509	-1.13578
202479_s_at	TRIB2	tribbles homolog 2 (Drosophila)	0.020081	2.14087	1.0982
205151_s_at	TRIL	TLR4 interactor with leucine-rich repeats	0.00037	1.49429	0.579461
202342_s_at	TRIM2	tripartite motif-containing 2	0.044347	0.766088	-0.38442
213293_s_at	TRIM22	tripartite motif-containing 22	0.031592	0.748918	-0.41712
210995_s_at	TRIM23	tripartite motif-containing 23	0.01622	0.729164	-0.45569
203846_at	TRIM32	tripartite motif-containing 32	0.012249	0.703025	-0.50835
202795_x_at	TRIOBP	TRIO and F-actin binding protein	0.035868	1.86979	0.902878
204033_at	TRIP13	thyroid hormone receptor interactor 13	0.017568	1.3331	0.414786
212435_at	tripartite motif-containing 33	chr1:114935402-114940298 (-) // 97.14 // p13.2	0.031207	0.740178	-0.43406
205802_at	TRPC1	transient receptor potential cation channel, subfamily C, member 1	0.024374	0.634859	-0.65549
217979_at	TSPAN13	tetraspanin 13	0.016238	0.612143	-0.70806
209263_x_at	TSPAN4	tetraspanin 4	0.011686	1.50503	0.589794
209890_at	TSPAN5	tetraspanin 5	0.029284	0.498661	-1.00387
212928_at	TSPYL4	TSPY-like 4	0.000701	0.666221	-0.58593
213122_at	TSPYL5	TSPY-like 5	0.022115	0.743562	-0.42748
221493_at	TSPY-like 1	chr6:116597888-116601090 (-) // 86.22 // q22.1	0.04819	0.72861	-0.45678
218972_at	TTC17	tetratricopeptide repeat domain 17	0.019345	0.744289	-0.42607
208663_s_at	TTC3	tetratricopeptide repeat domain 3	0.034261	0.506271	-0.98202
203049_s_at	TTC37	tetratricopeptide repeat domain 37	0.035066	0.667007	-0.58423
209660_at	TTR	transthyretin	0.029229	2.14966	1.10411
203690_at	TUBGCP3	tubulin, gamma complex associated protein 3	0.028425	0.602779	-0.7303
211758_x_at	TXNDC9	thioredoxin domain containing 9	0.031697	0.743381	-0.42783
218381_s_at	U2AF2	U2 small nuclear RNA auxiliary factor 2	0.029016	0.697094	-0.52058
219192_at	UBAP2	ubiquitin associated protein 2	0.01847	1.30374	0.382655
202333_s_at	UBE2B	ubiquitin-conjugating enzyme E2B (RAD6 homolog)	0.043991	0.74557	-0.42359
210024_s_at	UBE2E3	ubiquitin-conjugating enzyme E2E 3 (UBC4/5 homolog, yeast)	0.003251	0.66308	-0.59275
209142_s_at	UBE2G1	ubiquitin-conjugating enzyme E2G 1 (UBC7 homolog, yeast)	0.011279	0.684892	-0.54605
202346_at	UBE2K	ubiquitin-conjugating enzyme E2K (UBC1 homolog, yeast)	0.001713	0.570115	-0.81067
201649_at	UBE2L6	ubiquitin-conjugating enzyme E2L 6	0.002494	1.35651	0.439905
211285_s_at	UBE3A	ubiquitin protein ligase E3A	0.002375	0.659026	-0.60159
213128_s_at	ubiquitin protein ligase E3A	chr15:25582331-25583783 (-) // 93.46 // q11.2	0.036287	0.73785	-0.4386
209115_at	ubiquitin-like modifier activating enzyme 3	chr3:69103881-69129517 (-) // 98.87 // p14.1	0.009687	0.579328	-0.78755
215884_s_at	UBQLN2	ubiquilin 2	0.016721	0.667012	-0.58422
218050_at	UFM1	ubiquitin-fold modifier 1	0.049388	0.704533	-0.50526
208358_s_at	UGT8	UDP glycosyltransferase 8	0.021748	0.405484	-1.30228
202706_s_at	UMPS	uridine monophosphate synthetase	0.01467	1.51834	0.6025
201672_s_at	USP14	ubiquitin specific peptidase 14 (tRNA-guanine transglycosylase)	0.004357	0.727589	-0.45881
214843_s_at	USP33	ubiquitin specific peptidase 33	0.016893	0.375731	-1.41223
207365_x_at	USP34	ubiquitin specific peptidase 34	0.021448	0.615979	-0.69905

219675_s_at	UXS1	UDP-glucuronate decarboxylase 1	0.01912	0.592472	-0.75518
213326_at	VAMP1	vesicle-associated membrane protein 1 (synaptobrevin 1)	0.001951	0.611812	-0.70884
200931_s_at	VCL	vinculin	0.023477	1.34491	0.427512
214785_at	VPS13A	vacuolar protein sorting 13 homolog A (S. cerevisiae)	0.007982	0.616837	-0.69704
218171_at	VPS4B	vacuolar protein sorting 4 homolog B (S. cerevisiae)	0.046947	0.662496	-0.59402
203798_s_at	VSNL1	visinin-like 1	0.042839	0.351115	-1.50998
204165_at	WASF1	WAS protein family, member 1	0.005507	0.674362	-0.56841
217822_at	WBP11	WW domain binding protein 11	0.031173	0.725072	-0.4638
203855_at	WDR47	WD repeat domain 47	0.008539	0.648547	-0.62472
217734_s_at	WDR6	WD repeat domain 6	0.002135	1.43839	0.524457
202908_at	WFS1	Wolfram syndrome 1 (wolframin)	0.013003	1.34832	0.431168
203827_at	WIPI1	WD repeat domain, phosphoinositide interacting 1	0.04319	1.47991	0.565513
202749_at	WRB	tryptophan rich basic protein	0.002141	0.743966	-0.42669
213734_at	WSB2	WD repeat and SOCS box-containing 2	0.003905	0.459753	-1.12107
203137_at	WTAP	Wilms tumor 1 associated protein	0.021643	0.68893	-0.53757
212638_s_at	WW domain containing E3 ubiquitin protein ligase 1	chr8:87386269-87480588 (+) // 99.15 // q21.3	2.22E-05	0.579012	-0.78834
206698_at	XK	X-linked Kx blood group (McLeod syndrome)	0.030745	0.652415	-0.61614
212160_at	XPOT	exportin, tRNA (nuclear export receptor for tRNAs)	0.001303	0.553446	-0.85349
216338_s_at	Yip1 domain family, member 3	chr6:43479571-43484640 (-) // 81.6 // p21.1	0.045081	1.44531	0.531379
201352_at	YME1L1	YME1-like 1 (S. cerevisiae)	0.001058	0.681473	-0.55327
217812_at	YTHDF2	YTH domain family, member 2	0.018963	0.740784	-0.43288
217717_s_at	YWHAB	tyrosine 3-monooxygenase/tryptophan 5-monooxygenase activation protein, beta polypeptide	0.0106	0.555045	-0.84932
200638_s_at	YWHAZ	tyrosine 3-monooxygenase/tryptophan 5-monooxygenase activation protein, zeta polypeptide	0.037502	0.662651	-0.59368
200047_s_at	YY1	YY1 transcription factor	0.017039	0.669531	-0.57878
203043_at	ZBED1	zinc finger, BED-type containing 1	0.040164	1.38446	0.469328
204847_at	ZBTB11	zinc finger and BTB domain containing 11	0.00331	0.571986	-0.80595
201593_s_at	ZC3H15	zinc finger CCCH-type containing 15	0.013665	0.429963	-1.21772
213051_at	ZC3HAV1	zinc finger CCCH-type, antiviral 1	0.021273	1.53145	0.614897
218020_s_at	ZFAND3	zinc finger, AN1-type domain 3	0.000299	1.45618	0.542188
217367_s_at	ZHX3	zinc fingers and homeoboxes 3	0.045336	1.69458	0.760928
213064_at	zinc finger CCCH-type containing 14	chr14:89078158-89080003 (+) // 99.4 // q31.3	0.008432	0.654489	-0.61156
209049_s_at	ZMYND8	zinc finger, MYND-type containing 8	0.03535	0.615114	-0.70108
37254_at	ZNF133	zinc finger protein 133	0.005728	1.3545	0.437755
207394_at	ZNF137P	zinc finger protein 137, pseudogene	0.015221	0.571539	-0.80708
219854_at	ZNF14	zinc finger protein 14	0.004735	0.40352	-1.30929
212366_at	ZNF292	zinc finger protein 292	0.0164	0.646007	-0.63038
219376_at	ZNF322B	zinc finger protein 322B	0.025884	0.650483	-0.62042
50376_at	ZNF444	zinc finger protein 444	0.012653	1.47504	0.560753
217627_at	ZNF573	zinc finger protein 573	0.033009	0.729326	-0.45536
206188_at	ZNF623	zinc finger protein 623	0.018838	0.503347	-0.99038
220760_x_at	ZNF665	zinc finger protein 665	0.035014	0.496403	-1.01041
213659_at	ZNF75D	zinc finger protein 75D	0.006925	1.75187	0.808899
215758_x_at	ZNF93	zinc finger protein 93	0.045915	0.655958	-0.60833
213876_x_at	ZRSR2	zinc finger (CCCH type), RNA-binding motif and serine/arginine rich 2	0.008193	1.45754	0.543533
213605_s_at	---	chr5:69193430-69194769 (+) // 83.55 // q13.2 /// chr5:69786	0.031751	0.659424	-0.60072
210723_x_at	---	---	0.045732	1.54345	0.62616
222180_at	---	---	0.02739	1.46461	0.55052
213675_at	---	---	0.000889	1.3457	0.428358
213658_at	---	---	0.008717	1.32348	0.40434
221798_x_at	---	---	0.005893	1.30844	0.387851
214949_at	---	chr8:42984376-42985929 (+) // 3.44 // p11.21	0.045961	0.635888	-0.65315

215825_at	---	---	0.013044	1.49387	0.579054
222207_x_at	---	---	0.018152	0.681992	-0.55217
213048_s_at	---	---	0.024217	0.556025	-0.84678
213657_s_at	---	---	0.0081	1.31799	0.398339
222111_at	---	---	0.017143	0.428837	-1.2215
217679_x_at	---	---	0.011595	0.644435	-0.63389
200749_at	---	---	0.026783	0.698713	-0.51723
214848_at	---	---	0.037092	0.547238	-0.86976
216791_at	---	---	0.01268	2.24011	1.16357
213448_at	---	---	0.005158	1.30086	0.379463
217629_at	---	---	0.006546	1.40446	0.490014
215854_at	---	---	0.018221	1.47592	0.561615
91682_at	---	---	0.006028	1.91511	0.937427
207688_s_at	---	---	0.024443	0.445083	-1.16785
206936_x_at	---	---	0.015294	0.725584	-0.46279

STUDY3

Table 4: DE genes and probesets, occurring from comparison of SZ and control gene expression profiles. In the case that different probesets correspond to the same gene symbol, the one depicting the higher fold change is shown.

GeneID	Gene Symbol	Gene Title	p-value	Fold Change (natural) SZ vs CONTROL	Fold Change (log2) SZ vs CONTROL
203505_at	ABCA1	ATP-binding cassette, sub-family A (ABC1), member 1	0.046438	1.32561	0.406656
205013_s_at	ADORA2A/// SPECC1L	adenosine A2a receptor /// sperm antigen with calponin homology and coiled-coil domains 1-like	0.029586	1.48633	0.57175
204174_at	ALOX5AP	arachidonate 5-lipoxygenase-activating protein	0.014142	1.33505	0.416889
202203_s_at	AMFR	autocrine motility factor receptor	0.033985	0.754571	-0.40627
1569040_s_at	ANKRD36BP2	ankyrin repeat domain 36B pseudogene 2	0.016924	1.45042	0.536475
204671_s_at	ANKRD6	ankyrin repeat domain 6	0.00477	1.50589	0.590616
210067_at	AQP4	aquaporin 4	0.041896	1.36538	0.449303
241380_at	ARHGEF37	Rho guanine nucleotide exchange factor (GEF) 37	0.046491	0.697932	-0.51884
201954_at	ARPC1B	actin related protein 2/3 complex, subunit 1B, 41kDa	0.020764	1.31237	0.392173
217911_s_at	BAG3	BCL2-associated athanogene 3	0.022145	1.30221	0.380959
37547_at	BBS9	Bardet-Biedl syndrome 9	0.006067	0.707159	-0.49989
231960_at	BRWD1	bromodomain and WD repeat domain containing 1	0.036223	1.3346	0.41641
202953_at	C1QB	complement component 1, q subcomponent, B chain	0.023028	1.43282	0.518858
214428_x_at	C4A /// C4B /// LOC100509001	complement component 4A (Rodgers blood group) /// complement component 4B (Chido blood group) /// complement C4-B-like	0.016448	1.32022	0.400777
220770_s_at	C5orf54	chromosome 5 open reading frame 54	0.024377	1.33907	0.421231
230516_at	C7orf30	Chromosome 7 open reading frame 30	0.007903	1.67288	0.742334
218507_at	C7orf68	chromosome 7 open reading frame 68	0.020919	1.30907	0.388546
1559419_at	CACNB2	calcium channel, voltage-dependent, beta 2 subunit	0.03844	1.30372	0.382633
214475_x_at	CAPN3	calpain 3, (p94)	0.008507	0.759154	-0.39754
266_s_at	CD24	CD24 molecule	0.008027	1.37596	0.460437
217881_s_at	CDC27	cell division cycle 27 homolog (S. cerevisiae)	0.044428	1.34213	0.424523
203968_s_at	CDC6	cell division cycle 6 homolog (S. cerevisiae)	0.01659	0.695344	-0.5242

230364_at	CHPT1	choline phosphotransferase 1	0.037063	1.38457	0.46944
1555469_a_at	CLASP2	cytoplasmic linker associated protein 2	0.018268	0.581268	-0.78273
223699_at	CNDP1	carnosine dipeptidase 1 (metallopeptidase M20 family)	0.00055	0.729908	-0.45421
227253_at	CP	ceruloplasmin (ferroxidase)	0.01587	1.37804	0.462618
242539_at	DIS3L2	DIS3 mitotic control homolog (S. cerevisiae)-like 2	0.009376	1.31927	0.399739
200664_s_at	DNAJB1	DnaJ (Hsp40) homolog, subfamily B, member 1	0.021825	1.3568	0.440204
213092_x_at	DNAJC9	DnaJ (Hsp40) homolog, subfamily C, member 9	0.020375	1.36931	0.453451
1558501_at	DNM3	dynamin 3	0.038318	1.44463	0.5307
1565149_at	DYNC2H1	dynein, cytoplasmic 2, heavy chain 1	0.017371	1.65031	0.722738
203729_at	EMP3	epithelial membrane protein 3	0.021214	1.39706	0.482389
236314_at	EPM2AIP1	EPM2A (laforin) interacting protein 1	0.041875	0.676189	-0.5645
209527_at	EXOSC2	exosome component 2	0.000293	1.32097	0.401603
230067_at	FAM124A	Family with sequence similarity 124A	0.005991	0.704593	-0.50514
220170_at	FHL5	four and a half LIM domains 5	0.026865	1.33733	0.41936
1554966_a_at	FILIP1L	filamin A interacting protein 1-like	0.026255	1.53149	0.614937
217335_at	FLJ11292	hypothetical protein FLJ11292	0.021552	0.70947	-0.49519
227550_at	GFRA1	GDNF family receptor alpha 1	0.019344	1.34802	0.430844
33646_g_at	GM2A	GM2 ganglioside activator	0.008688	0.726064	-0.46183
224839_s_at	GPT2	glutamic pyruvate transaminase (alanine aminotransferase) 2	0.005131	1.31779	0.398119
218468_s_at	GREM1	gremlin 1	0.005135	0.694008	-0.52698
220491_at	HAMP	hepcidin antimicrobial peptide	0.041509	1.3128	0.39265
225297_at	HAUS1	HAUS augmin-like complex, subunit 1	0.010134	0.669255	-0.57937
211990_at	HLA-DPA1	major histocompatibility complex, class II, DP alpha 1	0.025666	1.44649	0.532555
208894_at	HLA-DRA	major histocompatibility complex, class II, DR alpha	0.042588	1.35743	0.440875
204934_s_at	HPN	hepsin	0.016931	0.67343	-0.5704
205404_at	HSD11B1	hydroxysteroid (11-beta) dehydrogenase 1	0.000279	0.751038	-0.41304
210095_s_at	IGFBP3	insulin-like growth factor binding protein 3	0.007872	1.81268	0.858127
216557_x_at	IGHA1 /// IGHD /// IGHG1 /// IGHG3 /// IGHM /// IGHV3-48 /// IGHV4-31 /// LOC100291917	immunoglobulin heavy constant alpha 1 /// immunoglobulin heavy constant delta /// immunoglobulin heavy constant gamma 1 (G1m marker) /// immunoglobulin heavy constant gamma 3 (G3m marker) /// immunoglobulin heavy constant mu /// immunoglobulin heavy varia	0.040034	0.650971	-0.61934
202803_s_at	ITGB2	integrin, beta 2 (complement component 3 receptor 3 and 4 subunit)	0.021425	1.37706	0.461587
226750_at	LARP1B	La ribonucleoprotein domain family, member 1B	0.010577	1.71959	0.782066
1556231_a_at	LOC100507060	hypothetical LOC100507060	0.024845	1.4866	0.57202
210386_s_at	LOC100510712 /// MTX1	metaxin-1-like /// metaxin 1	0.024875	0.753727	-0.40789
228601_at	LOC401022	hypothetical LOC401022	0.004694	0.548073	-0.86756
240246_at	LOC642236	Similar to FRG1 protein (FSHD region gene 1 protein)	0.001182	1.80325	0.850595
1569110_x_at	LOC728613	programmed cell death 6 pseudogene	0.002421	0.5937	-0.75219
215112_x_at	MCF2L2	MCF.2 cell line derived transforming sequence-like 2	0.014483	1.41695	0.502788
200796_s_at	MCL1	myeloid cell leukemia sequence 1 (BCL2-related)	0.009933	1.67857	0.74723
217546_at	MT1M	metallothionein 1M	0.026905	1.40886	0.494527

212338_at	MYO1D	myosin ID	0.005009	0.525553	-0.92809
231996_at	N4BP2	NEDD4 binding protein 2	0.018845	1.33954	0.421736
228278_at	NFIX	nuclear factor I/X (CCAAT-binding transcription factor)	0.008153	0.638077	-0.6482
230883_at	NXPH2	neurexophilin 2	0.031585	0.739992	-0.43442
216422_at	PA2G4 /// PA2G4P4	proliferation-associated 2G4, 38kDa /// proliferation-associated 2G4 pseudogene 4	0.04901	0.748989	-0.41698
200906_s_at	PALLD	palladin, cytoskeletal associated protein	0.048564	1.42385	0.509794
239067_s_at	PANX2	pannexin 2	0.012904	0.678616	-0.55933
216804_s_at	PDLIM5	PDZ and LIM domain 5	0.04582	1.50406	0.588864
232288_at	PDXDC1 /// PDXDC2P	pyridoxal-dependent decarboxylase domain containing 1 /// pyridoxal-dependent decarboxylase domain containing 2, pseudogene	0.029763	1.36145	0.445141
209803_s_at	PHLDA2	pleckstrin homology-like domain, family A, member 2	0.001955	0.730672	-0.4527
202430_s_at	PLSCR1	phospholipid scramblase 1	0.009012	1.70094	0.766332
218476_at	POMT1	protein-O-mannosyltransferase 1	0.003807	1.49779	0.582835
244515_at	PSMD7	Proteasome (prosome, macropain) 26S subunit, non-ATPase, 7	0.031136	1.34352	0.426018
244050_at	PTPLAD2	protein tyrosine phosphatase-like A domain containing 2	0.002532	1.31495	0.39501
205336_at	PVALB	parvalbumin	0.018269	0.756129	-0.4033
234314_at	RALGAPA2	Ral GTPase activating protein, alpha subunit 2 (catalytic)	0.005291	1.31357	0.393489
209637_s_at	RGS12	regulator of G-protein signaling 12	0.037364	1.38891	0.473956
213397_x_at	RNASE4	ribonuclease, RNase A family, 4	0.002409	1.44737	0.533436
212589_at	RRAS2	related RAS viral (r-ras) oncogene homolog 2	0.002159	1.7084	0.772649
219037_at	RRP15	ribosomal RNA processing 15 homolog (S. cerevisiae)	0.012897	1.62123	0.697085
1552365_at	SCIN	scinderin	0.026615	1.59613	0.674578
211429_s_at	SERPINA1	serpin peptidase inhibitor, clade A (alpha-1 antiproteinase, antitrypsin), member 1	0.006314	1.40949	0.495177
227210_at	SFMBT2	Scm-like with four mbt domains 2	0.008617	1.44995	0.536
222217_s_at	SLC27A3	solute carrier family 27 (fatty acid transporter), member 3	0.034512	1.47697	0.562643
204430_s_at	SLC2A5	solute carrier family 2 (facilitated glucose/fructose transporter), member 5	0.015157	1.30985	0.389404
207069_s_at	SMAD6	SMAD family member 6	0.026703	1.76036	0.815874
244461_at	SPECC1	sperm antigen with calponin homology and coiled-coil domains 1	0.030942	1.33135	0.412893
201858_s_at	SRGN	serglycin	0.041818	1.33934	0.42152
1553037_a_at	SYN2	synapsin II	0.038717	1.67133	0.740998
209153_s_at	TCF3	transcription factor 3 (E2A immunoglobulin enhancer binding factors E12/E47)	0.010762	1.71131	0.775101
213191_at	TICAM1	toll-like receptor adaptor molecule 1	0.046878	0.734043	-0.44606
1569316_at	TRIM24	tripartite motif-containing 24	0.023069	1.35273	0.435877
218838_s_at	TTC31	tetratricopeptide repeat domain 31	0.033756	1.49216	0.577404
208998_at	UCP2	uncoupling protein 2 (mitochondrial, proton carrier)	0.027246	1.59182	0.670673
232180_at	UGP2	UDP-glucose pyrophosphorylase 2	0.03898	1.30346	0.382345
202133_at	WWTR1	WW domain containing transcription regulator 1	0.019296	1.47906	0.564683
214218_s_at	XIST	X (inactive)-specific transcript (non-protein coding)	0.011641	0.386219	-1.37251
223506_at	ZC3H8	zinc finger CCCH-type containing 8	0.034084	1.44332	0.529386
225634_at	ZC3HAV1	zinc finger CCCH-type, antiviral 1	0.036853	1.4974	0.582458
223137_at	ZDHHC4	zinc finger, DHHC-type containing 4	0.023248	1.37134	0.455581
232408_at	ZFYVE28	zinc finger, FYVE domain containing 28	0.03992	1.55457	0.636512
214781_at	---	---	0.041556	1.33403	0.415787
210723_x_at	---	---	0.042829	0.73294	-0.44823

215791_at	---	---	0.040533	1.34224	0.424648
216259_at	---	---	0.013293	0.66026	-0.59889
236922_at	---	---	0.000974	0.639084	-0.64592
239845_at	---	---	0.045335	1.41071	0.496424
239992_at	---	---	0.017378	1.44084	0.526913
241913_at	---	---	0.015095	1.48593	0.571362
241930_x_at	---	---	0.035463	0.736906	-0.44045
242747_at	---	---	0.029888	1.42356	0.509507
243037_at	---	---	0.04732	1.37027	0.454458
1565880_at	---	---	0.027776	0.707755	-0.49868
1563130_a_at	---	---	0.010532	1.63666	0.710755
1559455_at	---	---	0.019164	1.45553	0.541547
224051_at	---	---	0.001518	1.61025	0.687284
232962_x_at	---	---	0.038855	1.40444	0.489998
231484_at	---	---	0.012778	0.655443	-0.60946
229635_at	---	---	0.021841	1.3028	0.381621
229255_x_at	---	---	0.000771	1.40906	0.494731
228866_at	---	---	0.007028	1.38687	0.471834
231218_at	---	---	0.022915	1.74296	0.801538

STUDY4

Table 5: DE genes and probesets, occurring from comparison of SZ and control gene expression profiles. In the case that different probesets correspond to the same gene symbol, the one depicting the higher fold change is shown.

GeneID	Gene Symbol	Gene Title	p-value	Fold Change (natural) SZ vs CONTROL	Fold Change (log2) SZ vs CONTROL
AFFX-M27830_5_at	---	---	0.0178138	1.31166	0.391396
207622_s_at	ABCF2	ATP-binding cassette, sub-family F (GCN20), member 2	0.0435542	0.718633	-0.476673
209735_at	ABCG2	ATP-binding cassette, sub-family G (WHITE), member 2	4.2329E-05	0.662714	-0.593542
209600_s_at	ACOX1	acyl-CoA oxidase 1, palmitoyl	0.0113646	1.38483	0.469711
212984_at	activating transcription factor 2	chr2:175936986-175938895 (-) // 90.99 // q31.1	0.0331926	1.35107	0.434099
201401_s_at	ADRBK1	adrenergic, beta, receptor kinase 1	0.00742266	0.471594	-1.08438
221008_s_at	AGXT2L1	alanine-glyoxylate aminotransferase 2-like 1	0.00280399	1.76169	0.816962
205208_at	ALDH1L1	aldehyde dehydrogenase 1 family, member L1	0.0193707	2.08248	1.0583
205390_s_at	ANK1	ankyrin 1, erythrocytic	0.0249898	0.733004	-0.448106
219069_at	ANKRD49	ankyrin repeat domain 49	0.00395673	1.34758	0.430372
208103_s_at	ANP32E	acidic (leucine-rich) nuclear phosphoprotein 32 family, member E	0.0342486	1.37058	0.454788
205196_s_at	AP1S1	adaptor-related protein complex 1, sigma 1 subunit	0.00846132	0.578556	-0.789472
213419_at	APBB2	amyloid beta (A4) precursor protein-binding, family B, member 2	0.0477672	0.514435	-0.95894
201284_s_at	APEH	N-acylaminoacyl-peptide hydrolase	0.0496828	0.701103	-0.512301
207542_s_at	AQP1	aquaporin 1 (Colton blood group)	0.0210557	1.37953	0.464174
210906_x_at	AQP4	aquaporin 4	0.00374129	1.64099	0.714565
217936_at	ARHGAP5	Rho GTPase activating protein 5	0.0167656	1.30677	0.386009
202914_s_at	ARHGEF11	Rho guanine nucleotide exchange factor (GEF) 11	0.0220595	0.67541	-0.566165
201954_at	ARPC1B	actin related protein 2/3 complex, subunit 1B, 41kDa	0.0115091	0.660978	-0.597327
213387_at	ATAD2B	ATPase family, AAA domain	0.00914256	0.516972	-0.951843

		containing 2B			
202672_s_at	ATF3	activating transcription factor 3	0.0403793	0.724056	-0.465826
211586_s_at	ATP2B2	ATPase, Ca++ transporting, plasma membrane 2	0.0430141	0.480545	-1.05726
212136_at	ATP2B4	ATPase, Ca++ transporting, plasma membrane 4	0.0100493	1.31571	0.395846
214594_x_at	ATP8B1	ATPase, aminophospholipid transporter, class I, type 8B, member 1	0.00778418	1.36034	0.443965
209902_at	ATR	ataxia telangiectasia and Rad3 related	0.0408364	0.55509	-0.849207
205363_at	BBOX1	butyrobetaine (gamma), 2-oxoglutarate dioxygenase (gamma-butyrobetaine hydroxylase) 1	0.0444572	1.69053	0.757477
201169_s_at	BHLHE40	basic helix-loop-helix family, member e40	0.00603234	0.765696	-0.385156
204493_at	BID	BH3 interacting domain death agonist	0.0226159	0.738604	-0.437128
221599_at	C11orf67	chromosome 11 open reading frame 67	0.0332622	1.76816	0.822253
218721_s_at	C1orf27	chromosome 1 open reading frame 27	0.0169665	1.31849	0.398885
206339_at	CARTPT	CART prepropeptide	0.0453778	1.33974	0.421948
209970_x_at	CASP1	caspase 1, apoptosis-related cysteine peptidase (interleukin 1, beta, convertase)	0.0485653	0.758442	-0.398889
221912_s_at	CCDC28B	coiled-coil domain containing 28B	0.011268	1.33696	0.418952
209619_at	CD74	CD74 molecule, major histocompatibility complex, class II invariant chain	0.0347059	0.698578	-0.517507
208727_s_at	CDC42	cell division cycle 42 (GTP binding protein, 25kDa)	0.0259325	0.529528	-0.91722
219036_at	CEP70	centrosomal protein 70kDa	0.039069	1.3149	0.394948
211419_s_at	CHN2	chimerin (chimaerin) 2	0.0465038	0.666224	-0.585921
201897_s_at	CKS1B	CDC28 protein kinase regulatory subunit 1B	0.0411054	0.682999	-0.550044
204375_at	CLSTN3	calsyntenin 3	0.00997281	0.743571	-0.427458
213190_at	COG7	component of oligomeric golgi complex 7	0.0302755	0.517827	-0.949458
201942_s_at	CPD	carboxypeptidase D	0.018973	0.519213	-0.945603
205630_at	CRH	corticotropin releasing hormone	0.0439668	0.684393	-0.547104
212073_at	CSNK2A1	casein kinase 2, alpha 1 polypeptide	0.048401	0.640211	-0.64338
201219_at	CTBP2	C-terminal binding protein 2	0.0056003	1.46239	0.548332
203687_at	CX3CL1	chemokine (C-X3-C motif) ligand 1	3.5982E-05	0.723676	-0.466584
209687_at	CXCL12	chemokine (C-X-C motif) ligand 12	0.00229564	0.696202	-0.522422
221903_s_at	CYLD	cylindromatosis (turban tumor syndrome)	0.0128513	0.501916	-0.994483
221780_s_at	DDX27	DEAD (Asp-Glu-Ala-Asp) box polypeptide 27	0.0384823	0.665565	-0.587349
219402_s_at	DERL1	Der1-like domain family, member 1	0.0368488	0.52785	-0.9218
208086_s_at	DMD	dystrophin	0.0117295	0.622642	-0.683524
216652_s_at	down-regulator of transcription 1, TBP-binding (negative cofactor 2)	chr1:93820531-93826490 (+) // 75.32 // p22.1	0.010861	0.747733	-0.419405
201041_s_at	DUSP1	dual specificity phosphatase 1	0.0103403	0.716549	-0.480862
204557_s_at	DZIP1	DAZ interacting protein 1	0.00110895	1.50476	0.589536
203013_at	ECD	ecdysoneless homolog (Drosophila)	0.00756231	0.706998	-0.500221
201842_s_at	EFEMP1	EGF-containing fibulin-like extracellular matrix protein 1	0.041014	1.40376	0.489295
211698_at	EID1	EP300 interacting inhibitor of differentiation 1	0.046818	1.36153	0.445225
220363_s_at	ELMO2	engulfment and cell motility 2	0.0280491	0.550481	-0.861236
221950_at	EMX2	empty spiracles homeobox 2	0.00899125	1.96605	0.975302
201340_s_at	ENC1	ectodermal-neural cortex 1 (with	0.0126503	1.31772	0.398044

		BTB-like domain)			
32042_at	ENOX2	ecto-NOX disulfide-thiol exchanger 2	0.0271917	1.33897	0.421123
221487_s_at	ENSA	endosulfine alpha	0.0400026	0.740706	-0.433027
208958_at	ERP44	endoplasmic reticulum protein 44	0.00938532	1.41304	0.498802
218748_s_at	EXOC5	exocyst complex component 5	0.0152712	0.70053	-0.513482
222291_at	FAM149A	family with sequence similarity 149, member A	0.0437825	1.30405	0.382998
209943_at	FBXL4	F-box and leucine-rich repeat protein 4	0.0365036	1.64248	0.715873
209189_at	FOS	FBJ murine osteosarcoma viral oncogene homolog	0.012841	0.675751	-0.565437
219170_at	FSD1	fibronectin type III and SPRY domain containing 1	0.00738277	0.750903	-0.413302
221245_s_at	FZD5	frizzled homolog 5 (Drosophila)	0.0360109	1.5897	0.668751
208457_at	GABRD	gamma-aminobutyric acid (GABA) A receptor, delta	0.0319481	0.753625	-0.408081
204224_s_at	GCH1	GTP cyclohydrolase 1	0.0218528	0.717285	-0.479383
201667_at	GJA1	gap junction protein, alpha 1, 43kDa	0.0110327	1.61868	0.694821
200648_s_at	GLUL	glutamate-ammonia ligase	0.0217707	1.33672	0.418695
207174_at	GPC5	glypican 5	0.0283118	1.70841	0.772653
207183_at	GPR19	G protein-coupled receptor 19	0.0366277	1.37394	0.458321
218706_s_at	GRAMD3	GRAM domain containing 3	0.0291115	1.40703	0.492651
205862_at	GREB1	growth regulation by estrogen in breast cancer 1	0.00210548	1.54071	0.623598
214217_at	GRM5	glutamate receptor, metabotropic 5	0.038466	1.37079	0.455011
212294_at	guanine nucleotide binding protein (G protein), gamma 12	chr1:68167158-68170044 (-) // 92.84 // p31.3	0.0174544	1.33815	0.420235
215695_s_at	GYG2	glycogenin 2	0.0265914	1.60986	0.686934
206106_at	HDAC10 /// LOC100509694 /// MAPK12	histone deacetylase 10 /// mitogen-activated protein kinase 12-like /// mitogen-activated protein kinase 12	0.0104988	0.562225	-0.83078
203395_s_at	HES1	hairy and enhancer of split 1, (Drosophila)	0.0237492	1.30445	0.383441
208729_x_at	HLA-B	major histocompatibility complex, class I, B	0.00572352	0.721389	-0.471151
215193_x_at	HLA-DRB1 /// HLA-DRB3 /// HLA-DRB4	major histocompatibility complex, class II, DR beta 1 /// major histocompatibility complex, class II, DR beta 3 /// major histocompatibility complex, class II, DR beta 4	0.0112083	0.72872	-0.456564
209312_x_at	HLA-DRB1 /// HLA-DRB4 /// HLA-DRB5	major histocompatibility complex, class II, DR beta 1 /// major histocompatibility complex, class II, DR beta 4 /// major histocompatibility complex, class II, DR beta 5	0.00334864	0.750401	-0.414267
217456_x_at	HLA-E	major histocompatibility complex, class I, E	0.0408373	0.758461	-0.398853
208808_s_at	HMGB2	high-mobility group box 2	0.0256216	1.30103	0.379651
205600_x_at	HOXB5	homeobox B5	0.0130494	1.354	0.437232
205404_at	HSD11B1	hydroxysteroid (11-beta) dehydrogenase 1	0.00059786	0.688451	-0.538573
209292_at	ID4	Inhibitor of DNA binding 4, dominant negative helix-loop-helix protein	0.0462184	1.50285	0.587701
201163_s_at	IGFBP7	insulin-like growth factor binding protein 7	0.0429326	1.32484	0.40582
219255_x_at	IL17RB	interleukin 17 receptor B	0.0105717	1.32874	0.410056
218637_at	IMPACT	Impact homolog (mouse)	0.00830989	1.42354	0.509484
207191_s_at	ISLR	immunoglobulin superfamily containing leucine-rich repeat	0.0237879	0.720219	-0.473493
202660_at	ITPR2	inositol 1,4,5-triphosphate receptor, type 2	0.0397942	1.38485	0.469727

212396_s_at	KIAA0090	KIAA0090	0.00478249	0.376926	-1.40765
218486_at	KLF11	Kruppel-like factor 11	0.0330145	1.33757	0.419612
219371_s_at	KLF2	Kruppel-like factor 2 (lung)	0.0239724	1.33938	0.421567
222006_at	LETM1	leucine zipper-EF-hand containing transmembrane protein 1	0.00335286	1.47581	0.561503
205876_at	LIFR	leukemia inhibitory factor receptor alpha	0.00185112	1.92379	0.943954
46142_at	LMF1	lipase maturation factor 1	0.0405631	1.47895	0.564575
215009_s_at	LOC100499177	hypothetical LOC100499177	0.039042	1.46079	0.546753
221589_s_at	LOC100506517	hypothetical LOC100506517	0.0121813	1.36456	0.448435
218816_at	LRRC1	leucine rich repeat containing 1	0.00526008	1.84093	0.880436
220219_s_at	LRRC37A /// LRRC37A2 /// LRRC37A3 /// LRRC37A4	leucine rich repeat containing 37A /// leucine rich repeat containing 37, member A2 /// leucine rich repeat containing 37, member A3 /// leucine rich repeat containing 37, member A4 (pseudogene)	0.00843067	1.87072	0.903594
220143_x_at	LUC7L	LUC7-like (S. cerevisiae)	0.00625192	0.621447	-0.686298
218559_s_at	MAFB	v-maf musculoaponeurotic fibrosarcoma oncogene homolog B (avian)	0.00531972	0.529326	-0.917771
210075_at	MARCH2	membrane-associated ring finger (C3HC4) 2	0.00354162	1.34031	0.422567
211042_x_at	MCAM	melanoma cell adhesion molecule	0.00383797	1.49244	0.577677
212732_at	MEG3	maternally expressed 3 (non-protein coding)	0.00413837	1.32827	0.409548
207761_s_at	METTL7A	methyltransferase like 7A	0.00598114	1.44834	0.534404
203419_at	MLL4	myeloid/lymphoid or mixed-lineage leukemia 4	0.042659	1.54994	0.632208
215692_s_at	MPPED2	metallophosphoesterase domain containing 2	0.00981702	0.485496	-1.04247
218202_x_at	MRPL44	mitochondrial ribosomal protein L44	0.0155783	1.30924	0.388734
206461_x_at	MT1H	metallothionein 1H	0.0337458	1.39216	0.477326
204326_x_at	MT1X	metallothionein 1X	0.011423	1.51626	0.60052
202960_s_at	MUT	methylmalonyl CoA mutase	0.0198148	1.38761	0.472607
214087_s_at	MYBPC1	myosin binding protein C, slow type	0.00467201	1.37658	0.461093
214156_at	myosin VIIA and Rab interacting protein	chr3:40285936-40301809 (+) // 91.64 // p22.1	0.0152871	1.38116	0.465884
221899_at	N4BP2L2	NEDD4 binding protein 2-like 2	0.0424094	1.3009	0.379509
202906_s_at	NBN	nibrin	0.0487702	0.762628	-0.390948
209557_s_at	NCDN	neurochondrin	0.0465545	0.619773	-0.690187
219006_at	NDUFAF4	NADH dehydrogenase (ubiquinone) 1 alpha subcomplex, assembly factor 4	0.0194614	0.739466	-0.435443
204412_s_at	NEFH	neurofilament, heavy polypeptide	0.00641151	0.762079	-0.391988
219396_s_at	NEIL1	nei endonuclease VIII-like 1 (E. coli)	0.00314111	0.688559	-0.538348
206089_at	NELL1	NEL-like 1 (chicken)	0.0204342	0.715015	-0.483954
202443_x_at	NOTCH2	notch 2	0.0275804	1.32135	0.402017
214722_at	NOTCH2NL	notch 2 N-terminal like	0.0480192	1.30053	0.379101
206001_at	NPY	neuropeptide Y	0.0129114	0.754586	-0.406243
31637_s_at	NR1D1 /// THRA	nuclear receptor subfamily 1, group D, member 1 /// thyroid hormone receptor, alpha (erythroblastic leukemia viral (v-erb-a) oncogene homolog, avian)	0.0129273	1.32168	0.402375
204622_x_at	NR4A2	nuclear receptor subfamily 4, group A, member 2	0.00101227	0.682989	-0.550066
209731_at	NTHL1	nth endonuclease III-like 1 (E. coli)	0.00712026	0.752557	-0.410126
221796_at	NTRK2	neurotrophic tyrosine kinase, receptor, type 2	0.0422784	1.36833	0.452416
37079_at	NUS1P3	nuclear undecaprenyl pyrophosphate synthase 1 homolog (S. cerevisiae) pseudogene 3	0.00636826	0.603435	-0.728729
206498_at	OCA2	oculocutaneous albinism II	0.00541132	0.465556	-1.10297

219523_s_at	ODZ3	odz, odd Oz/ten-m homolog 3 (Drosophila)	0.0478907	1.41402	0.499798
210004_at	OLR1	oxidized low density lipoprotein (lectin-like) receptor 1	0.00789631	0.730393	-0.453255
201246_s_at	OTUB1	OTU domain, ubiquitin aldehyde binding 1	0.0195231	0.740683	-0.433071
200897_s_at	PALLD	palladin, cytoskeletal associated protein	0.023671	1.32063	0.401231
204564_at	PCGF3	polycomb group ring finger 3	0.0335803	0.521576	-0.939052
212094_at	PEG10	paternally expressed 10	0.00601114	1.35328	0.436459
215354_s_at	PELP1	proline, glutamate and leucine rich protein 1	0.0019141	1.54392	0.626596
213791_at	PENK	proenkephalin	0.00046519	0.701744	-0.510984
33760_at	PEX14	peroxisomal biogenesis factor 14	0.0328712	1.34259	0.425018
221788_at	PGM3	phosphoglucomutase 3	0.0267882	1.81632	0.861019
209803_s_at	PHLDA2	pleckstrin homology-like domain, family A, member 2	0.0140824	0.729218	-0.455578
217863_at	PIAS1	protein inhibitor of activated STAT, 1	0.010409	1.35049	0.433485
51146_at	PIGV	phosphatidylinositol glycan anchor biosynthesis, class V	0.00598913	1.62031	0.696268
202620_s_at	PLOD2	procollagen-lysine, 2-oxoglutarate 5-dioxygenase 2	0.00787825	1.37988	0.464543
218901_at	PLSCR4	phospholipid scramblase 4	0.0398071	1.37019	0.454373
202725_at	POLR2A	polymerase (RNA) II (DNA directed) polypeptide A, 220kDa	0.0265608	0.522785	-0.93571
209355_s_at	PPAP2B	phosphatidic acid phosphatase type 2B	0.0140932	1.5336	0.616923
210236_at	PPFIA1	protein tyrosine phosphatase, receptor type, f polypeptide (PTPRF), interacting protein (liprin), alpha 1	0.00781085	1.41527	0.501074
205053_at	PRIM1	primase, DNA, polypeptide 1 (49kDa)	0.0142047	1.47504	0.560751
214203_s_at	PRODH	proline dehydrogenase (oxidase) 1	0.040777	1.92796	0.947077
202126_at	PRPF4B	PRP4 pre-mRNA processing factor 4 homolog B (yeast)	0.044345	1.33409	0.415853
219392_x_at	PRR11	proline rich 11	0.0363957	1.39364	0.478861
219742_at	PRR7	proline rich 7 (synaptic)	0.0151191	0.684193	-0.547526
202659_at	PSMB10	proteasome (prosome, macropain) subunit, beta type, 10	0.025084	0.723487	-0.46696
201252_at	PSMC4	proteasome (prosome, macropain) 26S subunit, ATPase, 4	0.0220381	0.532887	-0.9081
212015_x_at	PTBP1	polypyrimidine tract binding protein 1	0.0260542	0.721665	-0.4706
204469_at	PTPRZ1	protein tyrosine phosphatase, receptor-type, Z polypeptide 1	0.0228827	1.31035	0.389957
205336_at	PVALB	parvalbumin	0.00042678	0.741216	-0.432033
200608_s_at	RAD21	RAD21 homolog (S. pombe)	0.028006	1.30458	0.383585
212170_at	RBM12	RNA binding motif protein 12	0.0236813	1.43816	0.524224
222026_at	RBM3	RNA binding motif (RNP1, RRM) protein 3	0.00766368	0.761091	-0.39386
202975_s_at	RHOBTB3	Rho-related BTB domain containing 3	0.0168848	1.32375	0.404632
216348_at	RPS17	ribosomal protein S17	0.0399442	1.44542	0.531491
208902_s_at	RPS28	ribosomal protein S28	0.0125817	0.544478	-0.877054
206306_at	RYR3	ryanodine receptor 3	0.00286034	1.32822	0.409489
201747_s_at	SAFB	scaffold attachment factor B	0.0191546	0.74927	-0.416443
202071_at	SDC4	syndecan 4	0.0288003	1.31442	0.394429
214790_at	SENP6	SUMO1/sentrin specific peptidase 6	0.033831	1.43504	0.521095
214096_s_at	SHMT2	serine hydroxymethyltransferase 2 (mitochondrial)	0.0247588	0.703273	-0.507843
208078_s_at	SIK1	salt-inducible kinase 1	0.0126581	0.701997	-0.510464
202800_at	SLC1A3	solute carrier family 1 (glial high affinity glutamate transporter), member 3	0.0136633	1.4554	0.541416
222217_s_at	SLC27A3	solute carrier family 27 (fatty acid	0.00238017	1.38829	0.473311

		transporter), member 3			
201801_s_at	SLC29A1	solute carrier family 29 (nucleoside transporters), member 1	0.00510447	0.680353	-0.555645
202497_x_at	SLC2A3	solute carrier family 2 (facilitated glucose transporter), member 3	0.00105463	0.52489	-0.929913
202667_s_at	SLC39A4 /// SLC39A7	solute carrier family 39 (zinc transporter), member 4 /// solute carrier family 39 (zinc transporter), member 7	0.00152731	0.728542	-0.456916
205918_at	SLC4A3	solute carrier family 4, anion exchanger, member 3	0.0253509	0.655255	-0.609871
203908_at	SLC4A4	solute carrier family 4, sodium bicarbonate cotransporter, member 4	0.0356655	1.41652	0.502353
207604_s_at	SLC4A7	solute carrier family 4, sodium bicarbonate cotransporter, member 7	0.0426864	1.30055	0.379124
217678_at	SLC7A11	solute carrier family 7, (cationic amino acid transporter, y+ system) member 11	0.0149492	1.4252	0.511164
220460_at	SLCO1C1	solute carrier organic anion transporter family, member 1C1	0.0271979	1.52503	0.608836
202936_s_at	SOX9	SRY (sex determining region Y)-box 9	0.0120076	1.48046	0.566044
200671_s_at	SPTBN1	spectrin, beta, non-erythrocytic 1	0.0112115	0.694135	-0.526712
200685_at	SRSF11	serine/arginine-rich splicing factor 11	0.00357544	1.42682	0.5128
200890_s_at	SSR1	signal sequence receptor, alpha	0.0124481	0.575031	-0.798288
213921_at	SST	somatostatin	0.0267975	0.677511	-0.561683
215772_x_at	succinate-CoA ligase, GDP-forming, beta subunit	chr12:94942021-94944325 (+) // 91.81 // q22 /// chr3:67425144-67705002 (-) // 96.33 // p14.1	0.0177898	1.31482	0.394868
203901_at	TAB1	TGF-beta activated kinase 1/MAP3K7 binding protein 1	0.0479787	0.590319	-0.760433
221508_at	TAOK3	TAO kinase 3	0.0215631	0.757374	-0.400923
216037_x_at	TCF7L2	transcription factor 7-like 2 (T-cell specific, HMG-box)	0.00990122	1.34442	0.42698
218872_at	TESC	tescalcin	0.00367411	0.684829	-0.546183
203093_s_at	TIMM44	translocase of inner mitochondrial membrane 44 homolog (yeast)	0.00253893	0.483117	-1.04955
220021_at	TMC7	transmembrane channel-like 7	0.00933412	0.698104	-0.518487
213678_at	TMEM151B	transmembrane protein 151B	0.00343226	0.685739	-0.544268
64900_at	TMEM231	transmembrane protein 231	0.0435827	1.5606	0.642099
217930_s_at	TOLLIP	toll interacting protein	0.00026357	0.61333	-0.705264
204530_s_at	TOX	thymocyte selection-associated high mobility group box	0.0043719	1.35137	0.434418
205150_s_at	TRIL	TLR4 interactor with leucine-rich repeats	0.0481635	1.32451	0.405464
210541_s_at	TRIM27	tripartite motif-containing 27	0.00300909	0.698063	-0.518571
212435_at	tripartite motif-containing 33	chr1:114935402-114940298 (-) // 97.14 // p13.2	0.00276744	1.30684	0.386078
221493_at	TSPY-like 1	chr6:116597888-116601090 (-) // 86.22 // q22.1	0.0106159	1.31356	0.393485
208664_s_at	TTC3	tetratricopeptide repeat domain 3	0.0102987	0.668504	-0.580991
214023_x_at	TUBB2B	tubulin, beta 2B	0.0354049	1.37996	0.46463
211431_s_at	TYRO3	TYRO3 protein tyrosine kinase	0.0427561	0.533541	-0.906328
207350_s_at	VAMP4	vesicle-associated membrane protein 4	0.00212426	0.583101	-0.778183
218055_s_at	WDR41	WD repeat domain 41	0.0371753	0.677639	-0.561412
204712_at	WIF1	WNT inhibitory factor 1	0.0497501	1.36856	0.452659
217785_s_at	YKT6	YKT6 v-SNARE homolog (S. cerevisiae)	0.0249743	0.652018	-0.617016
202456_s_at	ZER1	zer-1 homolog (C. elegans)	0.0140587	0.700452	-0.513643
219877_at	ZMAT4	zinc finger, matrin-type 4	0.0188928	0.428297	-1.22332
214715_x_at	ZNF160	zinc finger protein 160	0.0125981	1.30328	0.382148
215887_at	ZNF277	zinc finger protein 277	0.046441	0.695599	-0.523671
200808_s_at	ZYX	zyxin	0.00324369	0.679095	-0.558316

Table 6: DE genes and probesets, occurring from comparison of BD and control gene expression profiles. In the case that different probesets correspond to the same gene symbol, the one depicting the higher fold change is shown.

GeneID	Gene Symbol	Gene Title	p-value	Fold Change (natural) BD vs CONTROL	Fold Change (log2) BD vs CONTROL
204719_at	ABCA8	ATP-binding cassette, sub-family A (ABC1), member 8	0.004851	0.756649	-0.4023
201715_s_at	ACIN1	apoptotic chromatin condensation inducer 1	0.038094	0.734335	-0.44549
215728_s_at	ACOT7	acyl-CoA thioesterase 7	0.005577	0.617306	-0.69594
203861_s_at	ACTN2	actinin, alpha 2	0.032205	0.718211	-0.47752
221008_s_at	AGXT2L1	alanine-glyoxylate aminotransferase 2-like 1	0.01808	1.29761	0.375859
203722_at	aldehyde dehydrogenase 4 family, member A1	chr1:19197925-19229047 (-) // 99.36 // p36.13	0.02417	1.46749	0.553347
205208_at	ALDH1L1	aldehyde dehydrogenase 1 family, member L1	0.015998	1.51508	0.599394
208103_s_at	ANP32E	acidic (leucine-rich) nuclear phosphoprotein 32 family, member E	0.016181	1.24643	0.317804
211404_s_at	APLP2	amyloid beta (A4) precursor-like protein 2	0.036655	0.826408	-0.27508
203381_s_at	APOE	apolipoprotein E	0.048067	1.229	0.297487
221031_s_at	APOLD1	apolipoprotein L domain containing 1	0.00874	1.45581	0.541821
219637_at	ARMC9	armadillo repeat containing 9	0.004718	0.689254	-0.53689
208679_s_at	ARPC2	actin related protein 2/3 complex, subunit 2, 34kDa	0.002285	0.828	-0.2723
212819_at	ASB1	ankyrin repeat and SOCS box-containing 1	0.012731	1.29835	0.37668
206030_at	ASPA	aspartoacylase	0.010966	0.793034	-0.33454
203295_s_at	ATP1A2	ATPase, Na+/K+ transporting, alpha 2 polypeptide	0.012266	1.21885	0.285522
1861_at	BAD	BCL2-associated agonist of cell death	0.030163	0.809937	-0.30412
204493_at	BID	BH3 interacting domain death agonist	0.029767	0.82017	-0.28601
204820_s_at	BTN3A2 /// BTN3A3	butyrophilin, subfamily 3, member A2 /// butyrophilin, subfamily 3, member A3	0.027109	1.32367	0.404543
203571_s_at	C10orf116	chromosome 10 open reading frame 116	0.009695	1.3643	0.448164
204073_s_at	C11orf9	chromosome 11 open reading frame 9	0.024898	0.828615	-0.27123
214668_at	C13orf1	chromosome 13 open reading frame 1	0.000888	1.24065	0.311096
213237_at	C16orf88	chromosome 16 open reading frame 88	0.020462	0.797319	-0.32677
217767_at	C3	complement component 3	0.049549	0.819585	-0.28704
220889_s_at	CA10	carbonic anhydrase X	0.046167	0.81427	-0.29642
208853_s_at	CANX	calnexin	0.047452	0.80559	-0.31188
200722_s_at	CAPRIN1	cell cycle associated protein 1	0.034145	0.814042	-0.29683
37425_g_at	CCHCR1	coiled-coil alpha-helical rod protein 1	0.002323	1.55724	0.638988
209619_at	CD74	CD74 molecule, major histocompatibility complex, class II invariant chain	0.027624	0.805756	-0.31158
203198_at	CDK9	cyclin-dependent kinase 9	0.027733	1.21045	0.275547
203493_s_at	CEP57	centrosomal protein 57kDa	0.041412	1.24367	0.314607
204577_s_at	CLUAP1	clusterin associated protein 1	0.03654	0.802817	-0.31686

209083_at	CORO1A	coronin, actin binding protein, 1A	0.016829	0.660818	-0.59767
202226_s_at	CRK	v-crk sarcoma virus CT10 oncogene homolog (avian)	0.031376	0.733777	-0.44659
211038_s_at	CROCCP2	ciliary rootlet coiled-coil, rootletin pseudogene 2	0.043509	0.814655	-0.29574
209687_at	CXCL12	chemokine (C-X-C motif) ligand 12	0.009763	0.825038	-0.27747
213533_at	D4S234E	DNA segment on chromosome 4 (unique) 234 expressed sequence	0.021883	0.774364	-0.36892
221780_s_at	DDX27	DEAD (Asp-Glu-Ala-Asp) box polypeptide 27	0.017723	0.682639	-0.55081
219111_s_at	DDX54	DEAD (Asp-Glu-Ala-Asp) box polypeptide 54	0.018883	0.756546	-0.4025
219402_s_at	DERL1	Der1-like domain family, member 1	0.022666	0.663066	-0.59278
207831_x_at	DHPS	deoxyhypusine synthase	0.044431	0.704991	-0.50432
204800_s_at	DHRS12	dehydrogenase/reductase (SDR family) member 12	0.004042	0.819981	-0.28634
208891_at	DUSP6	dual specificity phosphatase 6	0.045503	0.792562	-0.3354
213787_s_at	EBP	emopamil binding protein (sterol isomerase)	0.00293	0.793647	-0.33343
201749_at	ECE1	endothelin converting enzyme 1	0.048058	1.36727	0.451293
204400_at	EFS	embryonal Fyn-associated substrate	0.048111	0.812969	-0.29873
207768_at	EGR4	early growth response 4	0.020514	0.656182	-0.60783
208670_s_at	EID1	EP300 interacting inhibitor of differentiation 1	0.036347	0.731686	-0.4507
208290_s_at	EIF5	eukaryotic translation initiation factor 5	0.015381	0.810333	-0.30341
221950_at	EMX2	empty spiracles homeobox 2	0.016708	1.45961	0.545582
204824_at	ENDOG	endonuclease G	0.019759	0.688111	-0.53929
210839_s_at	ENPP2	ectonucleotide pyrophosphatase/phosphodiesterase 2	0.008147	0.815256	-0.29467
204160_s_at	ENPP4	ectonucleotide pyrophosphatase/phosphodiesterase 4 (putative)	0.038139	0.773741	-0.37008
203348_s_at	ETV5	ets variant 5	0.003747	0.776717	-0.36454
91684_g_at	EXOSC4	exosome component 4	0.031379	0.785463	-0.34838
219429_at	FA2H	fatty acid 2-hydroxylase	0.007881	0.759853	-0.39621
221687_s_at	FAM125B	family with sequence similarity 125, member B	0.009599	0.785617	-0.3481
219872_at	FAM198B	family with sequence similarity 198, member B	0.031572	1.22216	0.289428
38043_at	FAM3A	family with sequence similarity 3, member A	0.035018	0.767432	-0.38189
213669_at	FCHO1	FCH domain only 1	0.018335	0.647971	-0.626
203647_s_at	FDX1	ferredoxin 1	0.01619	0.730749	-0.45255
213746_s_at	FLNA	filamin A, alpha	0.041347	1.23409	0.303444
217487_x_at	FOLH1	folate hydrolase (prostate-specific membrane antigen) 1	0.0199	0.564668	-0.82453
210933_s_at	FSCN1	fascin homolog 1, actin-bundling protein (Strongylocentrotus purpuratus)	0.038277	0.803168	-0.31623
219170_at	FSD1	fibronectin type III and SPRY domain containing 1	0.04121	0.760078	-0.39578
208457_at	GABRD	gamma-aminobutyric acid (GABA) A receptor, delta	0.030417	0.82924	-0.27014
206849_at	GABRG2	gamma-aminobutyric acid (GABA) A receptor, gamma 2	0.030142	0.764995	-0.38648
206102_at	GINS1	GINS complex subunit 1 (Psf1 homolog)	0.018185	1.33766	0.419709
201667_at	GJA1	gap junction protein, alpha 1, 43kDa	0.042197	1.25903	0.332313
218361_at	GOLPH3L	golgi phosphoprotein 3-like	0.021897	1.23315	0.302352
207174_at	GPC5	glypican 5	0.006726	1.50681	0.591493
218151_x_at	GPR172A	G protein-coupled receptor 172A	0.008721	1.60463	0.682236
209631_s_at	GPR37	G protein-coupled receptor 37	0.028863	0.823597	-0.27999

		(endothelin receptor type B-like)			
205862_at	GREB1	growth regulation by estrogen in breast cancer 1	0.000974	1.33666	0.418634
206534_at	GRIN2A	glutamate receptor, ionotropic, N-methyl D-aspartate 2A	0.002639	0.832798	-0.26396
201415_at	GSS	glutathione synthetase	0.018319	0.677015	-0.56274
218238_at	GTPBP4	GTP binding protein 4	0.04576	0.798671	-0.32433
203395_s_at	HES1	hairy and enhancer of split 1, (Drosophila)	0.004336	1.25639	0.32929
201137_s_at	HLA-DPB1	major histocompatibility complex, class II, DP beta 1	0.003405	0.779282	-0.35978
215193_x_at	HLA-DRB1 /// HLA-DRB3 /// HLA-DRB4	major histocompatibility complex, class II, DR beta 1 /// major histocompatibility complex, class II, DR beta 3 /// major histocompatibility complex, class II, DR beta 4	0.001276	0.775956	-0.36595
205404_at	HSD11B1	hydroxysteroid (11-beta) dehydrogenase 1	0.002971	0.828391	-0.27162
203089_s_at	HTRA2	HtrA serine peptidase 2	0.032988	0.690132	-0.53506
209291_at	ID4	inhibitor of DNA binding 4, dominant negative helix-loop-helix protein	0.034141	1.21178	0.277129
35776_at	ITSN1	intersectin 1 (SH3 domain protein)	0.038205	1.21887	0.285548
204678_s_at	KCNK1	potassium channel, subfamily K, member 1	0.008797	0.823443	-0.28026
211713_x_at	KIAA0101	KIAA0101	0.000467	1.26638	0.340708
201552_at	LAMP1	lysosomal-associated membrane protein 1	0.036831	0.802575	-0.31729
222006_at	LETM1	leucine zipper-EF-hand containing transmembrane protein 1	0.009685	1.21157	0.276883
216336_x_at	LOC100505584 /// MT1E	hypothetical protein LOC100505584 /// metallothionein 1E	0.010381	1.21669	0.282957
210386_s_at	LOC100510712 /// MTX1	metaxin-1-like /// metaxin 1	0.014992	0.615717	-0.69966
213502_x_at	LOC91316	glucuronidase, beta/immunoglobulin lambda-like polypeptide 1 pseudogene	0.042932	1.42478	0.51074
218589_at	LPAR6	lysophosphatidic acid receptor 6	0.003847	0.830529	-0.2679
205710_at	LRP2	low density lipoprotein receptor-related protein 2	0.049857	0.751102	-0.41292
205105_at	MAN2A1	mannosidase, alpha, class 2A, member 1	0.008533	0.799171	-0.32342
213014_at	MAPK8IP1	mitogen-activated protein kinase 8 interacting protein 1	0.007764	0.653433	-0.61389
208603_s_at	MAPK8IP2	mitogen-activated protein kinase 8 interacting protein 2	0.017584	0.640414	-0.64292
203841_x_at	MAPRE3	microtubule-associated protein, RP/EB family, member 3	0.011897	0.561211	-0.83339
210136_at	MBP	myelin basic protein	0.009354	0.79412	-0.33257
209087_x_at	MCAM	melanoma cell adhesion molecule	0.022238	0.831754	-0.26577
207761_s_at	METTL7A	methyltransferase like 7A	0.024195	1.21601	0.282159
203637_s_at	MID1	midline 1 (Opitz/BBB syndrome)	0.019488	1.21794	0.284441
211836_s_at	MOG	myelin oligodendrocyte glycoprotein	0.007261	0.704146	-0.50605
211595_s_at	MRPS11	mitochondrial ribosomal protein S11	0.007826	0.74519	-0.42432
218654_s_at	MRPS33	mitochondrial ribosomal protein S33	0.02492	0.823151	-0.28077
206461_x_at	MT1H	metallothionein 1H	0.019344	1.25656	0.329479
217546_at	MT1M	metallothionein 1M	0.031053	1.46286	0.548788
204326_x_at	MT1X	metallothionein 1X	0.015936	1.27812	0.35402
214961_at	MTUS2	microtubule associated tumor suppressor candidate 2	0.016268	1.24718	0.318668
204382_at	NAT9	N-acetyltransferase 9 (GCN5-	0.039665	1.33042	0.411877

		related, putative)			
206089_at	NELL1	NEL-like 1 (chicken)	0.004604	0.711131	-0.49181
220045_at	NEUROD6	neurogenic differentiation 6	0.009878	0.830608	-0.26776
211797_s_at	NFYC	nuclear transcription factor Y, gamma	0.029711	0.681674	-0.55285
218133_s_at	NIF3L1	NIF3 NGG1 interacting factor 3-like 1 (S. pombe)	0.037249	0.713047	-0.48793
206477_s_at	NOVA2	neuro-oncological ventral antigen 2	0.041299	1.45118	0.53723
210672_s_at	NPRL3	nitrogen permease regulator-like 3 (S. cerevisiae)	0.036324	1.22364	0.291177
213479_at	NPTX2	neuronal pentraxin II	0.034804	0.767388	-0.38197
203814_s_at	NQO2	NAD(P)H dehydrogenase, quinone 2	0.048308	0.758538	-0.39871
221795_at	NTRK2	neurotrophic tyrosine kinase, receptor, type 2	0.029822	1.20713	0.271585
202093_s_at	PAF1	Paf1, RNA polymerase II associated factor, homolog (S. cerevisiae)	0.023749	0.705359	-0.50357
212259_s_at	PBXIP1	pre-B-cell leukemia homeobox interacting protein 1	0.013699	1.61362	0.690303
207414_s_at	PCSK6	proprotein convertase subtilisin/kexin type 6	0.047484	0.737027	-0.44021
208911_s_at	PDHB	pyruvate dehydrogenase (lipoamide) beta	0.017786	0.813961	-0.29697
202671_s_at	PDXK	pyridoxal (pyridoxine, vitamin B6) kinase	0.036436	0.721585	-0.47076
217356_s_at	PGK1	phosphoglycerate kinase 1	0.030122	0.796531	-0.3282
213638_at	PHACTR1	phosphatase and actin regulator 1	0.012965	1.35921	0.442765
215236_s_at	PICALM	phosphatidylinositol binding clathrin assembly protein	0.036853	0.659515	-0.60052
204297_at	PIK3C3	phosphoinositide-3-kinase, class 3	0.032366	0.829734	-0.26928
205273_s_at	PITRM1	pitrilysin metallopeptidase 1	0.014346	0.680886	-0.55452
209581_at	PLA2G16	phospholipase A2, group XVI	0.041212	0.820882	-0.28475
216218_s_at	PLCL2	phospholipase C-like 2	0.018971	0.718588	-0.47676
219317_at	POLI	polymerase (DNA directed) iota	0.033108	0.801458	-0.3193
210573_s_at	POLR3C	polymerase (RNA) III (DNA directed) polypeptide C (62kD)	0.015843	0.792717	-0.33512
216330_s_at	POU6F1	POU class 6 homeobox 1	0.013123	0.655964	-0.60831
204284_at	PPP1R3C	protein phosphatase 1, regulatory (inhibitor) subunit 3C	0.022815	1.26473	0.338831
214545_s_at	PROSC	proline synthetase co-transcribed homolog (bacterial)	0.001882	0.60831	-0.71712
221547_at	PRPF18	PRP18 pre-mRNA processing factor 18 homolog (S. cerevisiae)	0.036391	0.766657	-0.38335
201274_at	PSMA5	proteasome (prosome, macropain) subunit, alpha type, 5	0.008087	0.766625	-0.38341
208827_at	PSMB6	proteasome (prosome, macropain) subunit, beta type, 6	0.019238	0.8036	-0.31545
200820_at	PSMD8	proteasome (prosome, macropain) 26S subunit, non-ATPase, 8	0.032963	0.825474	-0.27671
218670_at	PUS1	pseudouridylate synthase 1	0.019397	1.3093	0.38879
221810_at	RAB15	RAB15, member RAS onocogene family	0.018905	0.827305	-0.27351
212125_at	RANGAP1	Ran GTPase activating protein 1	0.023323	0.772386	-0.37261
208534_s_at	RASA4 /// RASA4P	RAS p21 protein activator 4 /// RAS p21 protein activator 4 pseudogene	0.038668	1.36487	0.448763
219286_s_at	RBM15	RNA binding motif protein 15	0.005123	1.22	0.286887
212430_at	RBM38	RNA binding motif protein 38	0.032135	1.43204	0.518068
209085_x_at	RFC1	replication factor C (activator 1) 1, 145kDa	0.029969	0.82743	-0.27329
1053_at	RFC2	replication factor C (activator 1) 2, 40kDa	0.044652	1.23752	0.307449
209441_at	RHOBTB2	Rho-related BTB domain containing 2	0.030693	0.788873	-0.34213

201785_at	RNASE1	ribonuclease, RNase A family, 1 (pancreatic)	0.009604	0.813051	-0.29858
206050_s_at	RNH1	ribonuclease/angiogenin inhibitor 1	0.023257	0.778592	-0.36106
221989_at	RPL10	ribosomal protein L10	0.02786	0.80176	-0.31876
204245_s_at	RPP14	ribonuclease P/MRP 14kDa subunit	0.023557	0.748967	-0.41703
216348_at	RPS17	ribosomal protein S17	0.020905	1.23115	0.300002
203379_at	RPS6KA1	ribosomal protein S6 kinase, 90kDa, polypeptide 1	0.015939	1.38287	0.467662
212589_at	RRAS2	related RAS viral (r-ras) oncogene homolog 2	0.000468	1.37056	0.454764
210251_s_at	RUFY3	RUN and FYVE domain containing 3	0.031737	0.818004	-0.28982
201825_s_at	SCCPDH	saccharopine dehydrogenase (putative)	0.021277	0.698449	-0.51777
218793_s_at	SCML1	sex comb on midleg-like 1 (Drosophila)	0.000448	1.34736	0.430132
41329_at	SCYL3	SCY1-like 3 (S. cerevisiae)	0.002799	0.769369	-0.37825
202071_at	SDC4	syndecan 4	0.00535	1.25178	0.323976
202375_at	SEC24D	SEC24 family, member D (S. cerevisiae)	0.00835	0.782287	-0.35423
216457_s_at	SF3A1	splicing factor 3a, subunit 1, 120kDa	0.00891	0.781139	-0.35635
204362_at	SKAP2	src kinase associated phosphoprotein 2	0.045844	0.776347	-0.36523
202800_at	SLC1A3	solute carrier family 1 (glial high affinity glutamate transporter), member 3	0.030534	1.23727	0.307166
205716_at	SLC25A40	solute carrier family 25, member 40	0.000748	0.796912	-0.32751
207604_s_at	SLC4A7	solute carrier family 4, sodium bicarbonate cotransporter, member 7	0.024398	1.20496	0.268983
212927_at	SMC5	structural maintenance of chromosomes 5	0.006881	1.2782	0.354117
202936_s_at	SOX9	SRY (sex determining region Y)-box 9	0.040487	1.22215	0.289424
202104_s_at	SPG7	spastic paraplegia 7 (pure and complicated autosomal recessive)	0.047275	0.828251	-0.27186
206433_s_at	SPOCK3	sparc/osteonectin, cwcv and kazal-like domains proteoglycan (testican) 3	0.025914	0.760642	-0.39471
209875_s_at	SPP1	secreted phosphoprotein 1	0.014141	0.798289	-0.32502
208803_s_at	SRP72	signal recognition particle 72kDa	0.038472	0.751951	-0.41129
200890_s_at	SSR1	signal sequence receptor, alpha	0.048338	0.749794	-0.41544
213921_at	SST	somatostatin	0.001702	0.718511	-0.47692
205339_at	STIL	SCL/TAL1 interrupting locus	0.017132	1.22966	0.298265
214512_s_at	SUB1	SUB1 homolog (S. cerevisiae)	0.00936	0.803939	-0.31484
210315_at	SYN2	synapsin II	0.030372	0.657532	-0.60487
205547_s_at	TAGLN	transgelin	0.044557	1.29768	0.375934
201263_at	TARS	threonyl-tRNA synthetase	0.004802	0.796626	-0.32803
218466_at	TBC1D17	TBC1 domain family, member 17	0.010291	0.820784	-0.28493
201759_at	TBCD	tubulin folding cofactor D	0.016336	1.252	0.324235
218872_at	TESC	tescalcin	0.031345	0.776737	-0.3645
203400_s_at	TF	transferrin	0.016893	0.799928	-0.32206
219663_s_at	TMEM121	transmembrane protein 121	0.041169	0.740816	-0.43281
213678_at	TMEM151B	transmembrane protein 151B	0.022543	0.795765	-0.32959
48531_at	TNIP2	TNFAIP3 interacting protein 2	0.01197	1.28769	0.364782
217960_s_at	TOMM22	translocase of outer mitochondrial membrane 22 homolog (yeast)	0.033805	0.664029	-0.59068
217959_s_at	TRAPPC4	trafficking protein particle complex 4	0.02211	0.831336	-0.2665
205151_s_at	TRIL	TLR4 interactor with leucine-rich repeats	0.001532	1.26873	0.343382
210541_s_at	TRIM27	tripartite motif-containing 27	0.039016	0.7621	-0.39195

201546_at	TRIP12	thyroid hormone receptor interactor 12	0.03419	0.775116	-0.36752
218838_s_at	TTC31	tetratricopeptide repeat domain 31	0.015266	0.709986	-0.49414
211177_s_at	TXNRD2	thioredoxin reductase 2	0.000233	2.02248	1.01612
208358_s_at	UGT8	UDP glycosyltransferase 8	0.023308	0.763029	-0.39019
217140_s_at	VDAC1	voltage-dependent anion channel 1	0.049351	0.82965	-0.26943
212156_at	VPS39	vacuolar protein sorting 39 homolog (S. cerevisiae)	0.049419	0.667981	-0.58212
218055_s_at	WDR41	WD repeat domain 41	0.009402	0.721452	-0.47103
204712_at	WIF1	WNT inhibitory factor 1	0.037843	1.22094	0.287994
213155_at	WSCD1	WSC domain containing 1	0.008364	1.33987	0.422092
210285_x_at	WTAP	Wilms tumor 1 associated protein	0.026322	0.732428	-0.44924
202932_at	YES1	v-yes-1 Yamaguchi sarcoma viral oncogene homolog 1	0.021645	1.26958	0.344348
205181_at	ZNF193	zinc finger protein 193	0.012483	0.776985	-0.36404
219379_x_at	ZNF358	zinc finger protein 358	0.02734	1.31811	0.398469
220215_at	ZNF669	zinc finger protein 669	0.00151	1.21875	0.285404
200808_s_at	ZYX	zyxin	0.031174	0.741283	-0.4319

Supplementary tables 7-12: The results of GO-analysis for each dataset are presented in supplementary tables 7-12. GOT p-value represents the hypergeometric test p-value score for each GO term. Enrichment represents the ratio of the number of times a GO term occurs in the examined DE gene list to the number of times this GO term exists in the list of the entire microarray (for each study, its respective Affymetrix platform). GO terms presented in italics, are the ones considered most interesting in our study.

STUDY 1

Table 7. Overrrepresented GO terms extracted from study 1 DE list, where SZ and control samples are compared.

GO Annotation	GOT p-value	Enrichment
translational elongation	5.91E-07	8/93
structural constituent of ribosome	1.10E-06	8/100
rRNA processing	0.000336181	4/957
potassium ion binding	0.000361033	5/989
nuclear mRNA splicing, via spliceosome	0.001812279	5/121
potassium ion transport	0.003079654	8/93
positive regulation of apoptosis	0.00373742	3/60
ubiquitin-protein ligase activity	0.005173701	4/105
RNA binding	0.007274219	10/441
ubiquitin-dependent protein catabolic process	0.009006948	4/120

Table 8. Overrepresented GO terms extracted from study 1 DE list, where BD and control samples are compared.

GO Annotation	GOT p-value	Enrichment
lipid catabolic process	0.002175245	4/59
membrane fraction	0.002327861	14/420
regulation of translation	0.002492239	3/37
synaptic transmission	0.003243421	7/158
nucleic acid binding	0.00364051	6/127
locomotory behavior	0.004332448	3/43
manganese ion binding	0.004690567	6/133
postsynaptic density	0.00552607	3/46
G-protein coupled receptor activity	0.01174764	8/237
transferase activity	0.017388799	24/1033
protein tyrosine kinase activity	0.018274083	3/65
ubiquitin thiolesterase activity	0.019439309	2/37
phosphoinositide binding	0.019439309	2/37
ER-Golgi intermediate compartment	0.019439309	2/37
nucleotide-excision repair, DNA damage removal	0.019439309	2/37
RNA polymerase II transcription factor activity, enhancer binding	0.020869644	2/38
Rho protein signal transduction	0.020869644	2/38
response to oxidative stress	0.029142436	3/75
apoptosis	0.032637231	8/283
GTPase activator activity	0.034505937	4/116
lamellipodium	0.038258712	2/48
nervous system development	0.038646235	8/292
G-protein coupled receptor protein signaling pathway	0.038646235	8/292
calcium ion transport	0.043155349	3/85
copper ion binding	0.049016005	2/53

STUDY 2

Table 9: Overrepresented GO terms extracted from study 2 DE list, where SZ and control samples are compared.

GO Annotation	GOT p-value	Enrichment
general RNA polymerase II transcription factor activity	9.83E-05	7/30
protein import into nucleus	0.000252	6/26
copper ion binding	0.000583	11/78
protein serine/threonine phosphatase activity	0.002441	7/47
magnesium ion binding	0.002551	37/459
spliceosome assembly	0.003664	5/30
protein targeting	0.003938	11/97
small GTPase mediated signal transduction	0.004303	25/291
translational initiation	0.004419	6/41
chromatin assembly or disassembly	0.006598	7/55
chloride channel activity	0.012043	5/38
galactosyltransferase activity	0.012825	4/28
protein ubiquitination	0.014684	7/63
vesicle-mediated transport	0.018626	10/105
postsynaptic membrane	0.026916	10/111
inositol or phosphatidylinositol phosphatase activity	0.028246	4/34
chloride transport	0.03851	6/62

STUDY 3

Table 10. Overrepresented GO terms extracted from study 3 DE list, where SZ and control samples are compared.

GO Annotation	GOT p-value	Enrichment
response to virus	0.000381	3/151
lipid metabolic process	0.000538	5/449
Centrosome	0.002317	4/409
Cilium	0.005629	2/160
mitochondrial outer membrane	0.006439	2/168
immune response	0.006525	5/741
Apoptosis	0.007902	5/772
inflammatory response	0.009786	3/371
transcription regulator activity	0.01035	2/200
regulation of Rho protein signal transduction	0.01288	2/217
transcription factor complex	0.017004	2/241
cellular component movement	0.017755	2/245
endoplasmic reticulum membrane	0.018524	2/249
actin binding	0.019807	4/692
cell division	0.023376	3/483
ATPase activity	0.027464	2/290

STUDY 4

Table 11. Overrepresented GO terms extracted from study 4 DE list, where SZ and control samples are compared.

GO Annotation	GOT p-value	Enrichment
cysteine-type peptidase activity	0.002970511	4/96
regulation of cell growth	0.00380474	5/146
calcium ion transport	0.005576709	5/158
copper ion binding	0.00818557	3/78
cellular calcium ion homeostasis	0.00932632	3/81
integral to plasma membrane	0.009585372	27/1731
defense response	0.009610784	4/127
nervous system development	0.011280274	10/487
transcription corepressor activity	0.01165986	5/185
transcription factor binding	0.01192839	4/134
transport	0.01622565	13/724
ATPase activity	0.017644823	4/148
immune response	0.019237735	12/668
synaptic transmission	0.019901413	6/268
nucleosome assembly	0.020232712	3/102
nucleic acid binding	0.023476607	13/760
cell adhesion	0.025131046	13/767
steroid hormone receptor activity	0.02895657	3/114
structural constituent of cytoskeleton	0.03184569	4/173
Synapse	0.035838499	3/122
zinc ion binding	0.035897075	32/2361
transporter activity	0.039610743	7/378
antigen processing and presentation	0.042167598	2/76
sequence-specific DNA binding	0.042235477	10/598
metabolic process	0.042836062	11/674
symporter activity	0.044617128	3/131
membrane	0.049976737	60/4943

Table 12. Overrepresented GO terms extracted from study 4 DE list, where BD and control samples are compared.

GO Annotation	GOT p-value	Enrichment
lipid catabolic process	0.002175245	4/59
membrane fraction	0.002327861	14/420
regulation of translation	0.002492239	3/37
synaptic transmission	0.003243421	7/158
nucleic acid binding	0.00364051	6/127
locomotory behavior	0.004332448	3/43
manganese ion binding	0.004690567	6/133
postsynaptic density	0.00552607	3/46
G-protein coupled receptor activity	0.01174764	8/237
response to wounding	0.016747269	2/35
transferase activity	0.017388799	24/1033
protein tyrosine kinase activity	0.018274083	3/65
ubiquitin thiolesterase activity	0.019439309	2/37
phosphoinositide binding	0.019439309	2/37
ER-Golgi intermediate compartment	0.019439309	2/37
nucleotide-excision repair, DNA damage removal	0.019439309	2/37
RNA polymerase II transcription factor activity, enhancer binding	0.020869644	2/38
Rho protein signal transduction	0.020869644	2/38
response to oxidative stress	0.029142436	3/75
apoptosis	0.032637231	8/283
GTPase activator activity	0.034505937	4/116
lamellipodium	0.038258712	2/48
nervous system development	0.038646235	8/292
G-protein coupled receptor protein signaling pathway	0.038646235	8/292
calcium ion transport	0.043155349	3/85
copper ion binding	0.049016005	2/53

Supplementary table 13: Kegg pathways based analysis. The lists of significantly altered genes from each study were submitted to StRAnGER analysis, elucidating over-represented Kegg terms.

Table 13. Statistical significant KEGG pathways in each study.

Study case	Overrepresented KEGG pathway
STUDY 1 SZ vs CONTROL	Alzheimer's disease - Homo sapiens (human)
	Neurotrophin signaling pathway - Homo sapiens (human)
STUDY1 BD vs CONTROL	Neurotrophin signaling pathway - Homo sapiens (human)
	Regulation of actin cytoskeleton - Homo sapiens (human)
	Cell adhesion molecules (CAMs) - Homo sapiens (human)
STUDY 2	Long-term potentiation - Homo sapiens (human)
STUDY 3	Regulation of actin cytoskeleton - Homo sapiens (human)
STUDY 4 BD vs CONTROL	MAPK signaling pathway - Homo sapiens (human)
STUDY 4 SZ vs CONTROL	MAPK signaling pathway - Homo sapiens (human)
